# Strong Anionic/Charge-Neutral Block Copolymers from Cu(0)-Mediated Reversible Deactivation Radical Polymerization

**DOI:** 10.1021/acs.macromol.2c01487

**Published:** 2022-09-26

**Authors:** Théophile Pelras, Anton H. Hofman, Lieke M. H. Germain, Anna M. C. Maan, Katja Loos, Marleen Kamperman

**Affiliations:** † Macromolecular Chemistry and New Polymeric Materials, Zernike Institute for Advanced Materials, University of Groningen, Nijenborgh 4, 9747 AG Groningen, The Netherlands; ‡ Polymer Science, Zernike Institute for Advanced Materials, University of Groningen, Nijenborgh 4, 9747 AG Groningen, The Netherlands

## Abstract

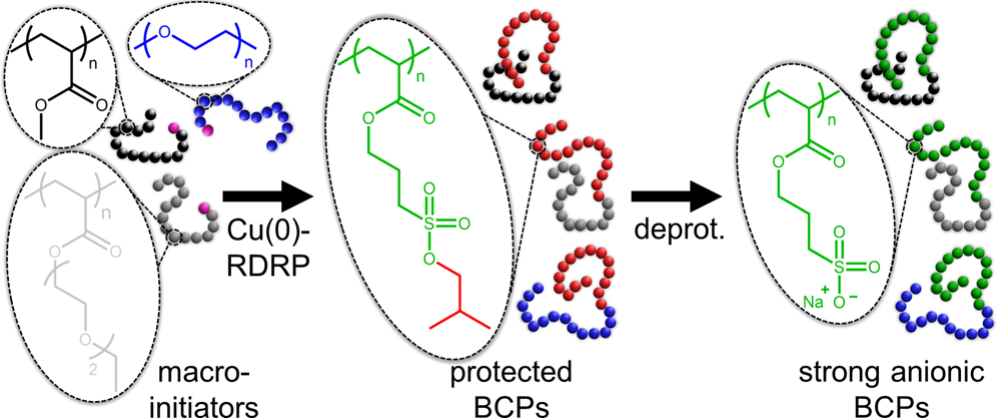

Despite recent developments in controlled polymerization techniques, the straightforward synthesis of block copolymers that feature both strong anionic and charge-neutral segments remains a difficult endeavor. In particular, solubility issues may arise during the direct synthesis of strong amphiphiles and typical postpolymerization deprotection often requires harsh conditions. To overcome these challenges, we employed Cu(0)-mediated reversible deactivation radical polymerization (Cu(0)-RDRP) on a hydrophobic isobutoxy-protected 3-sulfopropyl acrylate. Cu(0)-RDRP enables the rapid synthesis of the polymer, reaching high conversions and low dispersities while using a single solvent system and low amounts of copper species. These macromolecules are straightforward to characterize and can subsequently be deprotected in a mild yet highly efficient fashion to expose their strongly charged nature. Furthermore, a protected sulfonate segment could be grown from a variety of charge-neutral macroinitiators to produce, after the use of the same deprotection chemistry, a library of amphiphilic, double-hydrophilic as well as thermoresponsive block copolymers (BCPs). The ability of these various BCPs to self-assemble in aqueous media was further studied by dynamic light scattering, ζ-potential measurements as well as atomic force and electron microscopy.

## Introduction

Many systems in nature feature chemistries that remain difficult to straightforwardly mimic in a laboratory, most noticeably the presence of charges along polymer chains. A plethora of biomacromolecules, such as sodium alginate^1^ or heparan sulfate,^2^ rely on their anionic character to fulfill their respective roles within biological systems. The recent developments in controlled polymerization techniques have, however, vastly facilitated the production of synthetic precision-focused polyanions. The presence of charges amidst these macromolecules can be taken advantage of not only to produce synthetic counterparts of natural polymers but also to enable the build-up of soft matter into complex structures.^3^ Polyanions are capable of intermolecular^4−7^ or intramolecular^8,9^ interactions, which opens up to numerous applications including the chelation of underwater adhesives,^10,11^ the stabilization of inorganic nanoparticles,^12^ as well as the attachment of macrocyclic compounds,^13^ genetic materials,^14,15^ or therapeutics.^16,17^ Therefore, anionic/charge-neutral diblock, terblock, or star copolymers^18^ remain of high interest in soft matter science and have already demonstrated great capability as lubricants,^19^ surfactants for emulsion polymerization,^20,21^ surface modification agents,^22−24^ or for the fabrication of proton-exchange membrane fuel cells.^25^ However, the vast majority of these systems rely on the use of weak polyelectrolytes, which can drastically limit their range of applications, while strong polyelectrolytes suffer from limited solubility and remain far more challenging to produce and characterize.

Consequently, the installation of charges postpolymerization had remained the preferred methodology to produce polyanions. Thiol–ene coupling of sodium sulfate onto a polyallyl chain^26^ or the sulfonation of polystyrene^27,28^ have been reported but often requires harsh conditions and toxic reagents^27,29^ that may lead to various side reactions.^28^ Alternatively, protective groups,^30^ such as alkyl^31,32^ and fluorinated,^33^ can be installed onto styrene sulfonate prior to polymerization, but low monomer reactivity and difficulty to achieve high deprotection yields have hindered this route.

Several controlled polymerization techniques, including the ring-opening polymerization of aziridines,^34,35^ the nitroxide-mediated polymerization of sodium styrene sulfonate,^36,37^ or the reversible addition–fragmentation chain-transfer (RAFT) polymerization of (meth)acrylates,^38^ now permit the synthesis of anionic and even anionic/charge-neutral macromolecules, although monomer neutralization might be required to facilitate the synthesis.^39^ However, in spite of the recent developments in copper-catalyzed polymerization methods,^40^ the direct synthesis of strong polyanions *via* these techniques remains highly challenging. While surface-initiated atom transfer radical polymerization can be used to grow homopolymer chains from modified gold or silicon in water/methanol mixtures,^41^ difficulties persist for the production of macromolecules with more complex compositions, such as block and random copolymers. Amphiphilic block copolymers (BCPs) can be produced from unprotected potassium 3-sulfopropyl methacrylate^42^ or sodium styrene sulfonate,^43,44^ but the strong incompatibility between the two polymer domains requires the use of aqueous/organic solvent mixtures as well as high copper concentrations. Additionally, the lack of a common solvent for the synthesis of sulfonate-containing polymers further hinders proper characterization and limits the degree of chemical complexity the macromolecules can feature. A stabilizing agent, such as crown ether 18-crown-6, permits the rapid synthesis of strong polyanions in less stringent conditions.^45^ Although proven efficient, it is unclear if this method could be expanded to a larger range of comonomers. Consequently, postpolymerization deprotection of organosoluble precursors in mild conditions might remain a more efficient strategy for the synthesis of strong polyanions *via* copper-catalyzed polymerization techniques.

Amidst the array of user-friendly controlled polymerization techniques, copper(0)-reversible deactivation radical polymerization (Cu(0)-RDRP) has gained tremendous interest over the past few years since its first report.^46^ Short reaction times, low temperatures and amounts of catalyst, as well as preservation of end groups and low dispersity, even at high conversion, have pushed it to the forefront of user-friendly controlled polymerization techniques.^47,48^ Furthermore, this technique is highly versatile, permitting the tailoring of the polymers’ molecular weight distribution^49^ and is compatible with a wide range of monomers,^50^ most of which can be reacted in water or nontoxic dimethyl sulfoxide (DMSO). Despite the ever-growing catalogue of Cu(0)-RDRP-made polymers, there have been—to the best of our knowledge—no reports on the synthesis of sulfonate-containing BCPs with this technique.

Herein, we report the efficient synthesis of homo- and block copolymers featuring sodium sulfonate groups *via* Cu(0)-RDRP. This methodology is later applied to the production of a range of amphiphilic and double-hydrophilic anionic/charge-neutral diblock copolymers, which were later used for the formation of micelles in aqueous media.

## Results and Discussion

### Synthesis of Protected 3-Sulfopropyl Acrylate

**Figure 1:**
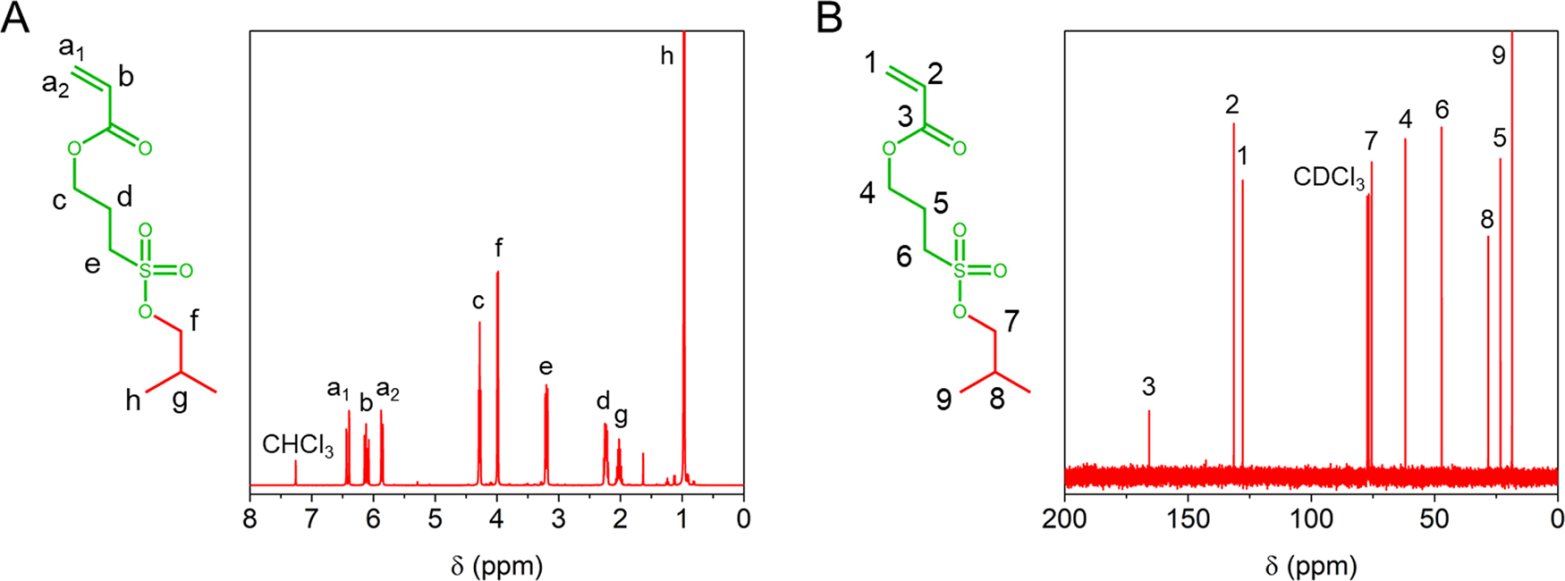
Nuclear magnetic resonance spectroscopy analyses of the BSPA monomer used in this study. (A) ^1^H NMR and (B) ^13^C NMR spectra recorded in CDCl_3_.

The potassium salt of 3-sulfopropyl acrylate was protected in a one-pot, two-step reaction, adapted from our previously reported procedure.^51^ The unprotected sulfonate monomer was first dispersed in *N*,*N*-dimethylformamide (DMF), before a small excess of oxalyl chloride dissolved in dichloromethane was added dropwise over the course of an hour under an inert atmosphere. This step yielded 3-chlorosulfopropyl acrylate, a highly reactive intermediate that was not isolated. The monomer precursor solution was then added dropwise under an inert atmosphere into a solution of isobutanol and triethylamine in dichloromethane to complete the esterification reaction. After stirring overnight, the crude product was extracted with diethyl ether and further purified by liquid–liquid extraction and flash chromatography to yield 3-isobutoxysulfopropyl acrylate (BSPA). The protected sulfonyl acrylate was obtained in high yield (∼80 mol %) and high purity, as confirmed by proton and carbon nuclear magnetic resonance spectroscopies (^1^H NMR and ^13^C NMR, respectively; Figure 1) as well as by heteronuclear single quantum coherence spectroscopy (Figure S1).

### Cu(0)-RDRP of Protected 3-Sulfopropyl Acrylate

**Figure 2:**
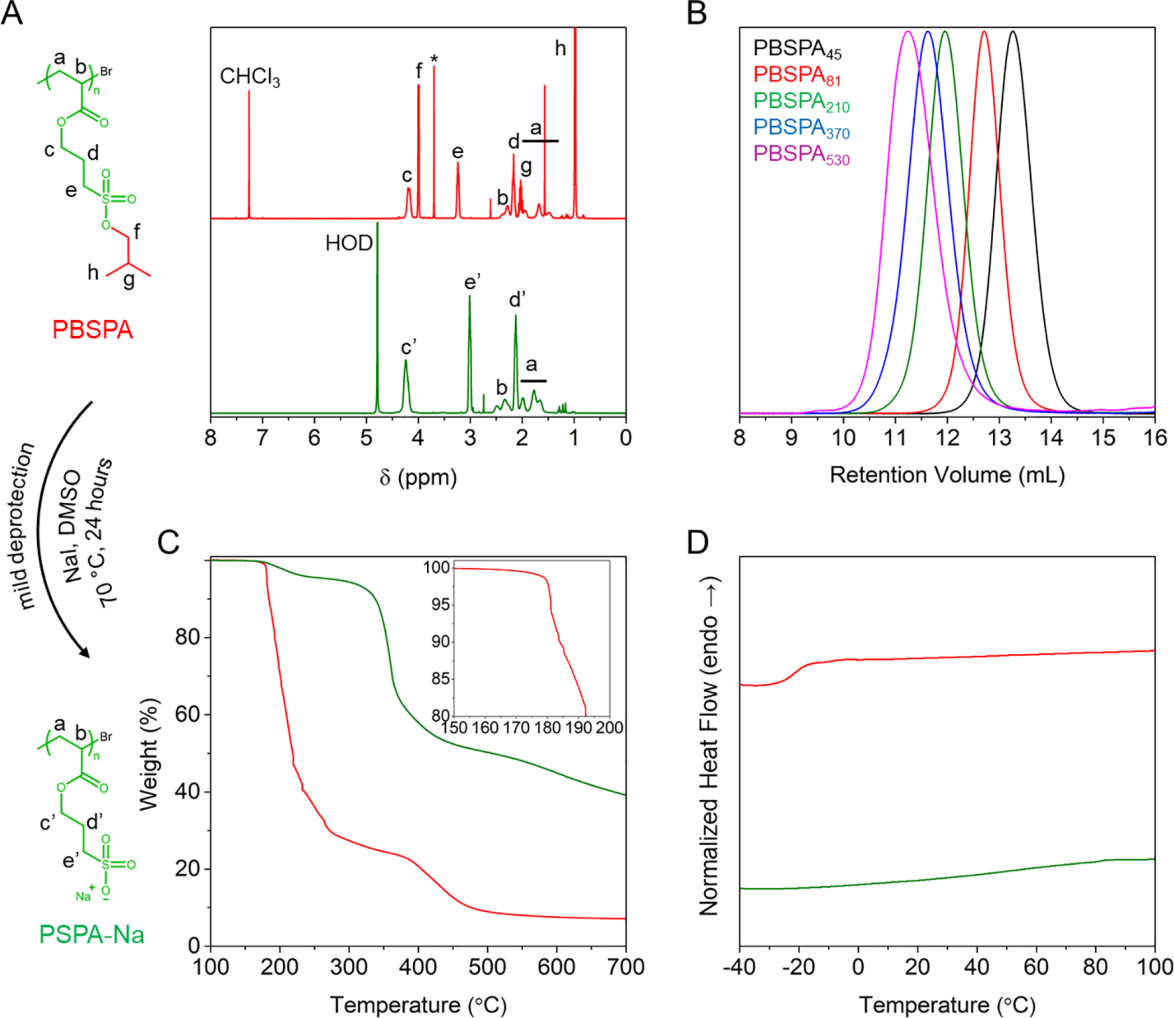
Characterization of poly(3-isobutoxysulfopropyl acrylate) homopolymers produced by Cu(0)-RDRP and their polyanionic counterparts obtained after deprotection with NaI in DMSO. (A) ^1^H NMR spectra of PBSPA_81_ (red, CDCl_3_, * = residual 1,4-dioxane) and corresponding PSPA_81_ (green, D_2_O). (B) SEC elugrams of PBSPA homopolymers of various molecular weights. (C) Thermogravimetric and (D) differential scanning calorimetry analyses on PBSPA_81_ (red) and corresponding PSPA-Na_81_ (green).

**Scheme 1:**
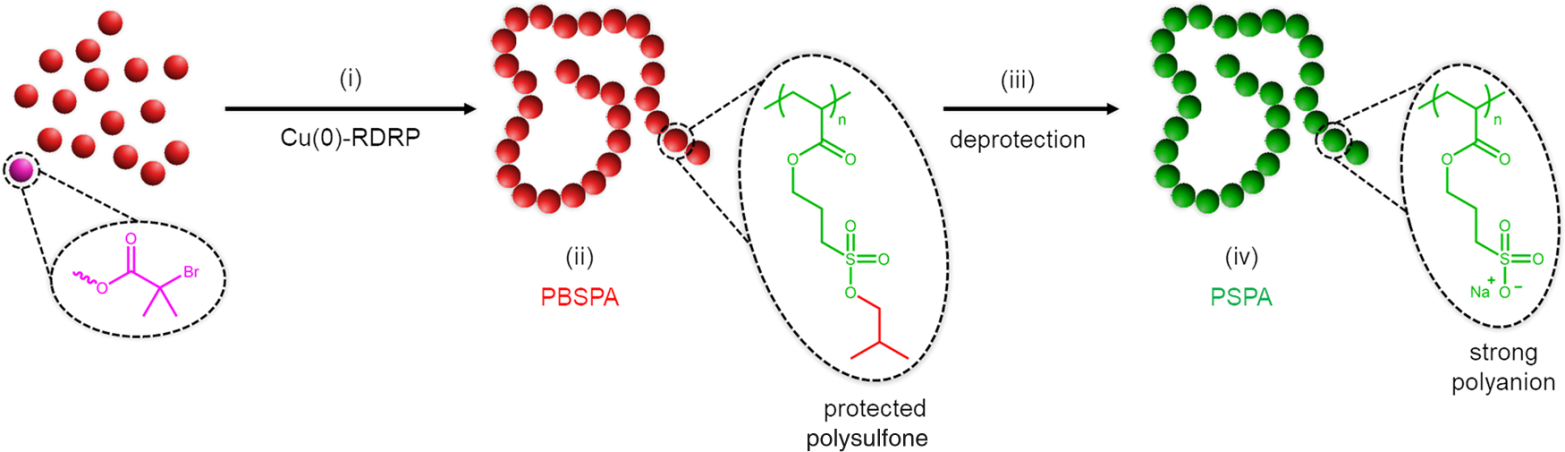
Schematic Depiction of the Synthesis of Strong Polyanions by Cu(0)-RDRP (i) Polymerization of 3-isobutoxysulfopropyl acrylate permits facile formation of (ii) protected homopolymers of various lengths and molecular weights. (iii) Subsequent nucleophilic deprotection under mild conditions enables efficient removal of the isobutoxy protective groups to yield (iv) strong polyanions.

The synthesis of strong polyanions necessitates a two-step procedure (Scheme 1): (i) the synthesis of isobutoxy-protected homopolymers by Cu(0)-RDRP of 3-isobutoxysulfopropyl acrylate and (ii) their subsequent deprotection using a strong nucleophile. We chose Cu(0)-RDRP because it enables the rapid polymerization of acrylates at room temperature and to high conversions while maintaining good control. In a first attempt, ethyl α-bromoisobutyrate (*i.e.*, the initiator), ∼50 equiv of BSPA, tris[2-(dimethylamino)ethyl]amine (Me_6_-TREN, *i.e.*, the ligand), and copper(II) bromide (CuBr_2_, *i.e.*, the deactivator) were dissolved in dimethyl sulfoxide (DMSO). We deliberately introduced low amounts of CuBr_2_ and Me_6_-TREN (respectively, 0.01 and 0.09 mol % to the initiator), as recent studies have demonstrated this to result in a more efficient polymerization^49^ and simultaneous improvement of the livingness and a higher chance of chain extension^52^ for large macromolecules at such ratios. After deoxygenation, a stirring bar wrapped with a freshly etched 4 cm copper wire was dropped into the mixture to start the polymerization. A high conversion (∼93%) was obtained within 4 h at room temperature, which enabled the facile and rapid synthesis of an isobutoxy-protected homopolymer (DP_PBSPA_ = 45, *M*_n,PBSPA45_ = 11.4 kDa), while keeping a low dispersity (*Đ*_PBSPA45_ = 1.12). Importantly, the protective groups remained untouched during the polymerization, as evidenced by ^1^H NMR from the matching ratios between proton signals of the isobutoxy and the alkyl spacer (Figure 2A). To test the robustness of Cu(0)-RDRP, we produced four other PBSPA homopolymers of various molecular weights, ranging up to 133.7 kDa (degree of polymerization (DP) = 530). Excellent control was observed for all homopolymers in size-exclusion chromatography (SEC; Figure 2B), with low dispersities and absence of tailing or chain–chain termination, even for the high-molecular-weight polymers. A noteworthy observation is that the final conversion typically diminishes with the increase of the target DP (Table 1), which could be explained by the large increase in viscosity of the reaction mixture over the course of the polymerization, eventually hampering full speed rotation of the stirring bar.

**Table 1:** Characteristics of the Protected Homopolymers Obtained through Cu(0)-RDRP of 3-Isobutoxysulfopropyl Acrylate

sample	[EB*i*B]/[BSPA]	conversion^a^ (%)	*M*_n NMR_^a^ (Da)	*M*_n SEC_^b^ (kDa)	*Đ*^b^	*T*_g_^c^ (°C)
PBSPA_45_	1:48	93	11.4	15.9	1.12	–27.9
PBSPA_81_	1:91	87	20.4	26.7	1.11	–21.4
PBSPA_210_	1:250	84	52.7	56.8	1.18	–25.2
PBSPA_370_	1:500	74	92.7	77.6	1.30	–13.9
PBSPA_530_	1:980	54	132.7	107.4	1.36	–13.1

a Determined from ^1^H NMR.

b Determined from SEC data in DMF using 0.01 M LiBr and calibrated against near-monodisperse poly(methyl methacrylate) (PMMA) standards.

c Determined from differential scanning calorimetry (DSC) using a 10 °C min^–1^ heating rate.

Next, a kinetic study of the BSPA homopolymerization under the same conditions but with ∼100 equiv of BSPA to initiator (*i.e.*, chosen to facilitate the isolation of early time points prior SEC analysis) was performed. Conversion reached ∼60% within the first 30 min, and almost all monomers were consumed within 2 h (∼91% conversion). A noteworthy feature is the deviation from the typical linear trend in the plot of ln([M]_0_/[M]) *vs* reaction time (Figure S2) after 1 h, which we attribute to the large increase in viscosity of the mixture and little amounts of remaining monomer.

Removal of the isobutoxy protective groups of the PBSPA homopolymers was first undertaken following a reported procedure,^51^*i.e.*, using 3 molar equiv of sodium iodide (NaI) in DMSO at 70 °C for 24 h. While this methodology needs a slightly elevated temperature, it remains far lower than that required for the thermal removal of similar protective groups on poly(styrene sulfonate).^31,32^^1^H NMR confirmed the quantitative removal of the isobutoxy protective groups and the production of strong polyanions, namely, poly(3-sulfopropyl acrylate) sodium salt (PSPA-Na; Figures 2A and S3-1). To expand the scope of this study, another iodide-based nucleophile, namely, 1-ethyl-3-methylimidazolium iodide (EMIMI), was used *in lieu* of NaI to produce strong polyanions (PSPA-EMIM) bearing larger and organic counterions (Figure S3-2). While the sodium salt of polyanions can be dissolved in a limited number of solvents (*e.g.*, water and DMSO; Figure S3-3), the presence of a methylimidazolium-based counterion slightly alters the polymer’s solubility, enabling its dissolution into a wider range of organics (*e.g.*, DMSO, methanol, and ethanol; Figure S3-4).

Next, thermal characterizations were performed on the PBSPA homopolymers and their deprotected counterparts, which further confirmed the successful removal of the isobutoxy protective groups, starting with thermogravimetric analyses (TGA; Figures 2C and S4-1 and Table S5). The protected homopolymers are stable up to 150 °C but start degrading around 160–180 °C with an initial loss of mass of ∼10–15% (kick visible in the inset of Figure 2C). This corresponds to the loss of the isobutoxy protective groups, which exposes the sulfonic acid moieties. The macromolecules further degrade at 194 ± 2 °C with a large weight loss of 72.4 ± 2.3%, attributed to the acid-catalyzed hydrolysis of the acrylic ester,^31^ before the final degradation of the backbone at 419 ± 9 °C, with a weight loss of 17.0 ± 1.2%. The early loss of mass is absent from the deprotected polymers, which are stable up to 250 °C (with a minor early weight loss attributed to traces of solvent) before a large weight loss of 73.6% occurs, attributed to the degradation of the sulfonyl groups. The PSPA-Na homopolymers further degrade at 419 °C with a final loss of 16.4% of their original weight. Differential scanning calorimetry (DSC; Figure 2D) performed on the PBSPA_81_ homopolymer revealed a low glass-transition temperature (*T*_g PBSPA81_ = −27.9 °C; Table 1). This low value compared to that of other protected sulfonyl polymers^53^ originates from the acrylic backbone of our system, with polyacrylates generally having a lower *T*_g_ than their methacrylic counterparts.^54^ The polymer chain length was observed to have a marginal influence on the observed *T*_g_ (Table 1 and Figure S4-2), with a maximum value at *T*_g PBSPA530_ = −13.1 °C for the homopolymer with the highest molecular weight. Further analyses performed on the deprotected PSPA-Na homopolymers did not reveal any glass-transition temperatures within the analysis range (Figure 2D), which corroborates their brittle and glassy nature compared to the very rubbery aspect of their protected counterparts. Interestingly, a glass-transition temperature was observed for EMIMI-deprotected homopolymers at around −20 °C (Figure S4-3), which marginally increases with the increase of polymer chain length. Thus, a change of nucleophile for the deprotection step enables the tailoring of not only the resulting polyanions’ solubility but also their thermal behaviors (Figure S4-4 and Table S6), a relationship that deserves further investigations in the future.

### Synthesis of Block Copolymers

**Figure 3:**
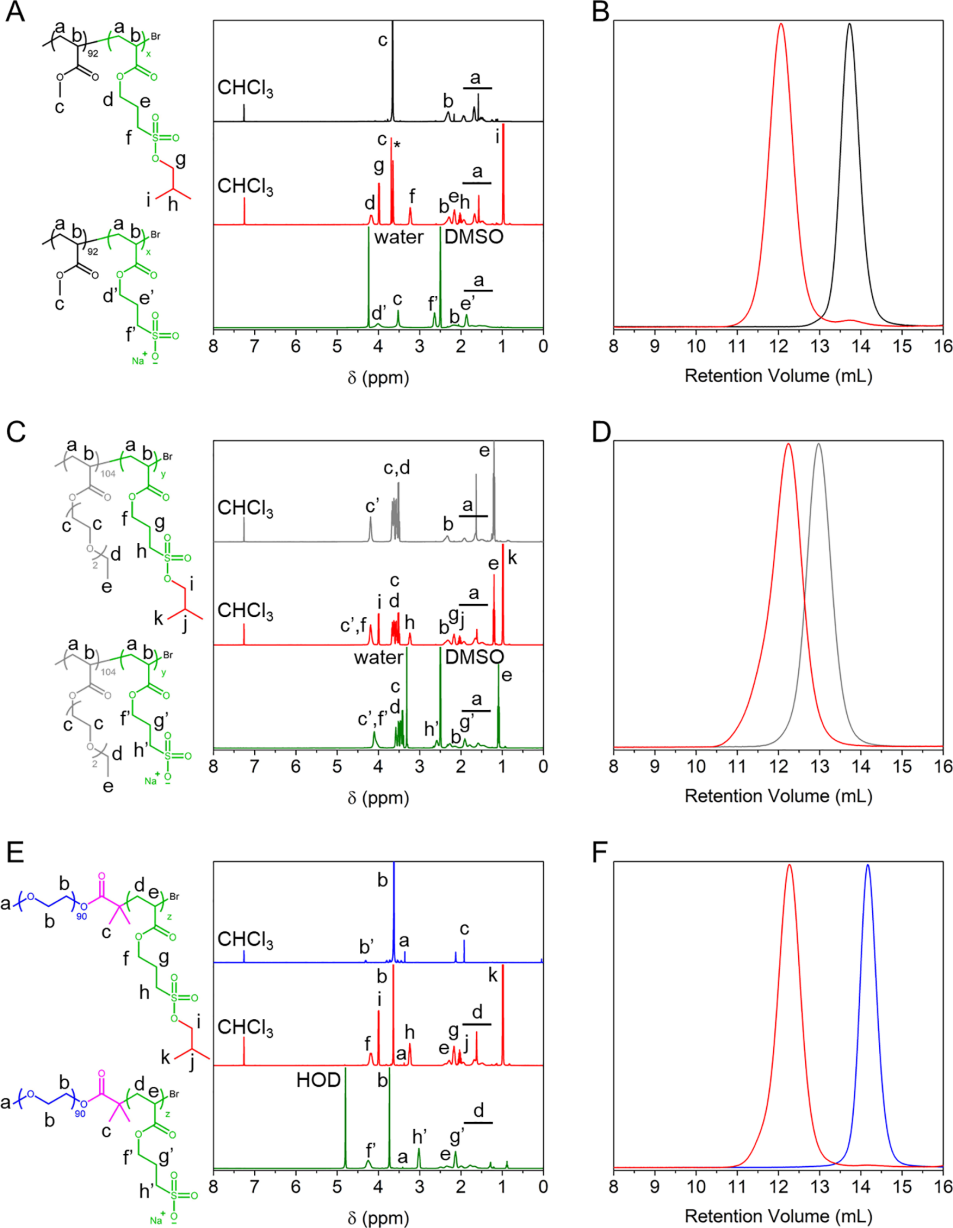
Characterization of various block copolymers produced in this study. ^1^H NMR spectra of (A) PMA-based block copolymers (PMA_92_, PMA_92_-*b*-PBSPA_103_, and PMA_92_-*b*-PSPA_103_), (C) poly(di[ethylene glycol] ethyl ether acrylate) (PDEGA)-based block copolymers (PDEGA_104_, PDEGA_104_-*b*-PBSPA_94_, and PDEGA_104_-*b*-PSPA-Na_94_), and (E) PEO-based block copolymers (PEO_90_-Br, PEO_90_-*b*-PBSPA_110_, and PEO_90_-*b*-PSPA_110_). SEC elugrams of the corresponding macroinitiators and precursor block copolymers, including (B) PMA_92_ and PMA_92_-*b*-PBSPA_103_, (D) PDEGA_104_ and PDEGA_104_-*b*-PBSPA_94_, as well as (F) PEO_90_ and PEO_90_-*b*-PBSPA_110_.

**Scheme 2:**
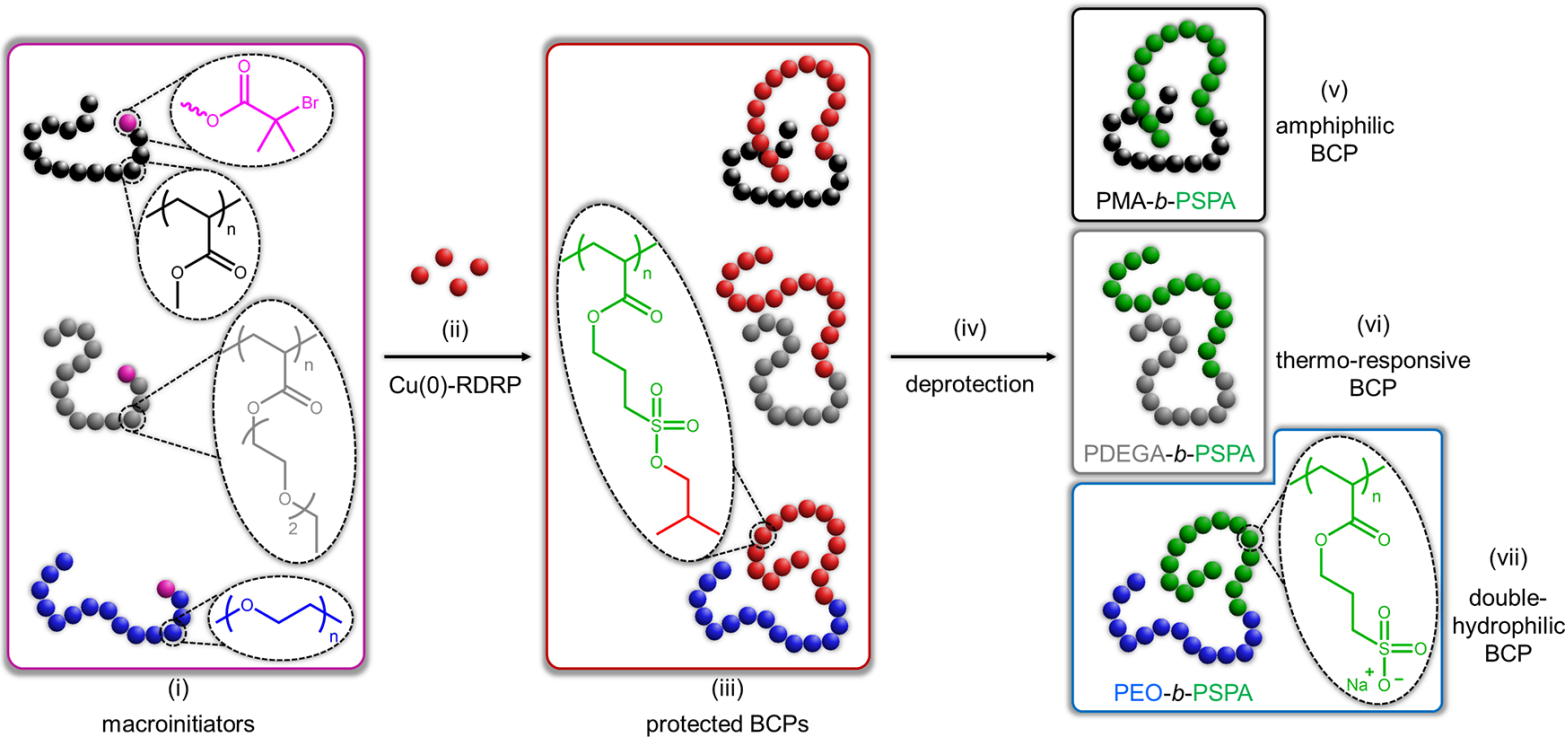
Schematic Illustration of the Synthesis of Block Copolymers Featuring a Strong Polyanionic Segment (i) Various macroinitiators, either produced by Cu(0)-RDRP or modified postpolymerization, can be (ii) chain-extended using BSPA monomer to yield (iii) a range of protected block copolymers. (iv) Their subsequent deprotection using NaI permits the removal of the isobutoxy groups and enables the formation of (v) amphiphilic, (vi) thermoresponsive, or (vii) double-hydrophilic block copolymers bearing a strong polyanionic segment.

To further demonstrate the versatility of Cu(0)-RDRP on our isobutoxy-protected monomer, we sought to produce a variety of BCPs (Scheme 2) by chain extension of macroinitiators. First, we produced a poly(methyl acrylate) (PMA) *via* Cu(0)-RDRP using low CuBr_2_ and Me_6_-TREN concentrations (respectively, 0.01 and 0.09 mol % to the initiator). Note that the reaction mixture was deoxygenated by three freeze–pump–thaw cycles instead of bubbling with inert gas due to the evaporation of the monomer at room temperature (Figure S5). We used ^1^H NMR conversion data to determine the polymer chain length and its corresponding molecular weight (DP = 92, *M*_n NMR_ = 8100 Da) and SEC to verify its narrow molecular weight distribution (*M*_n SEC_ = 10 700 Da, *Đ* = 1.07). This macroinitiator was then chain-extended with BSPA by Cu(0)-RDRP in DMSO at room temperature to yield two different BCPs: PMA_92_-*b*-PBSPA_103_ (*M*_n NMR_ = 30 100 Da, *Đ* = 1.13) and PMA_92_-*b*-PBSPA_231_ (*M*_n NMR_ = 33 900 Da, *Đ* = 1.24). ^1^H NMR on the purified polymers confirmed the successful addition of BSPA units onto the PMA_92_ macroinitiator (Figures 3A and S6-1) and enabled an accurate calculation of the overall chain lengths by comparison of the PBSPA isobutoxy CH_3_ signals (6H per BSPA, 1.0 ppm) to the PMA methyl signal (3H per MA, 3.6 ppm). Note that the comparison between the PBSPA CH_2_ signals (2H, 4.2 ppm and 2H, 3.2 ppm) and the PMA methyl signals provided the exact same results, which confirms that the isobutoxy protective groups are preserved during chain extension. Additionally, SEC (Figure 3B) confirmed the quantitative chain extension as well as the absence of chain–chain termination.

Next, we chose poly(di[ethylene glycol] ethyl ether acrylate) (PDEGA) as a macroinitiator for the chain extension with the BSPA monomer. PDEGA was produced in conditions identical to that of PMA, but the polymerization had to be stopped at ∼50% conversion to minimize chain–chain termination. This likely occurs because of the presence of a minor fraction of dimers.^55^^1^H NMR conversion data enabled the determination of the polymer chain length and its corresponding molecular weight (DP = 104, *M*_n NMR_ = 19 700 Da) and SEC to verify its narrow molecular weight distribution (*M*_n SEC_ = 22 000 Da, *Đ* = 1.11). Unlike PMA, which remains hydrophobic, PDEGA exhibits a reversible thermoresponsive character with a lower critical solution temperature (LCST_PDEGA104_ = 14 °C), as determined by dynamic light scattering and UV–vis spectroscopy (Figure S7), similarly to its more extensively studied methacrylic analogue.^56^ This property permits the production of block copolymers that can reversibly assemble upon change of the solution temperature. Chain extension with BSPA was performed by Cu(0)-RDRP to yield two different BCPs: PDEGA_104_-*b*-PBSPA_94_ (*M*_n NMR_ = 43 200 Da, *Đ* = 1.25) and PDEGA_104_-*b*-PBSPA_228_ (*M*_n NMR_ = 76 700 Da, *Đ* = 1.37). ^1^H NMR was invoked to confirm the presence of the protected sulfonyl block by comparison of the PBSPA CH_3_ and CH_2_ signals (6H at 1.0 ppm and 2H at 3.2 ppm, respectively) to the PDEGA methyl signal (3H, 1.2 ppm), while SEC was used to verify the homogeneous chain extension (Figures 3C,D and S6-2).

Finally, we sought to produce double-hydrophilic BCPs by chain extension of an ω-functionalized poly(ethylene oxide) (PEO_90_-Br) with BSPA. The macroinitiator was obtained through esterification of commercially available monohydroxy-terminated poly(ethylene oxide) (PEO_90_-OH) with α-bromoisobutyryl bromide in the presence of triethylamine. Successful esterification was confirmed by ^1^H NMR, while SEC showed no significant change in either average molecular weight or dispersity and absence of side reactions (Figure S6-3). Chain extension with BSPA *via* Cu(0)-RDRP enabled the production of two BCPs, PEO_90_*-b*-PBSPA_100_ (*M*_n NMR_ = 29 100 Da, *Đ* = 1.13) and PEO_90_-*b*-PBSPA_219_ (*M*_n NMR_ = 58 900 Da, *Đ* = 1.21). Again, a clear shift of the polymer signal was observed in SEC and the presence of the PBSPA signals was confirmed by ^1^H NMR, also enabling the calculation of the DP of PBSPA segments by comparison of the PEO CH_2_ signals (4H, 3.6 ppm) to the PBSPA CH_2_ signals (2H, 4.2 ppm) (Figures 3E,F and S6-4). Again, no reduction in the signal ratio of the isobutoxy protective group was observed upon chain extension. The characteristics of all block copolymers and their corresponding macroinitiators can be found in Table S4 in the Supporting Information.

Before undertaking the removal of isobutoxy groups from the BCPs, we performed negative control “deprotection” reactions using the same conditions as on the PBSPA homopolymers, to ensure that none of the PMA, PDEGA, or PEO segments would be affected. Both ^1^H NMR and SEC (Figure S8) analyses performed on the NaI-treated macroinitiators displayed peak signals, average molecular weights, and dispersities unchanged from that of the pristine macromolecules. This confirms the selective nature of the treatment by the nucleophile. Removal of the isobutoxy protective groups using NaI as a nucleophile was then conducted on all six BCPs, which enabled the production of two amphiphilic PMA_92_-*b*-PSPA-Na*_x_* (*x* = 103 or 231), two thermoresponsive PDEGA_104_-*b*-PSPA-Na*_y_* (*y* = 94 or 228) as well as two double-hydrophilic PEO_90_-*b*-PSPA-Na*_z_* (*z* = 110 or 237) block copolymers. ^1^H NMR confirmed the successful deprotection of the sulfonate groups in all systems (Figure 3). As a proof-of-concept, the removal of the isobutoxy protective groups was also performed using EMIMI *in lieu* of NaI as a nucleophile and confirmed by ^1^H NMR (Figure S9).

**Figure 4:**
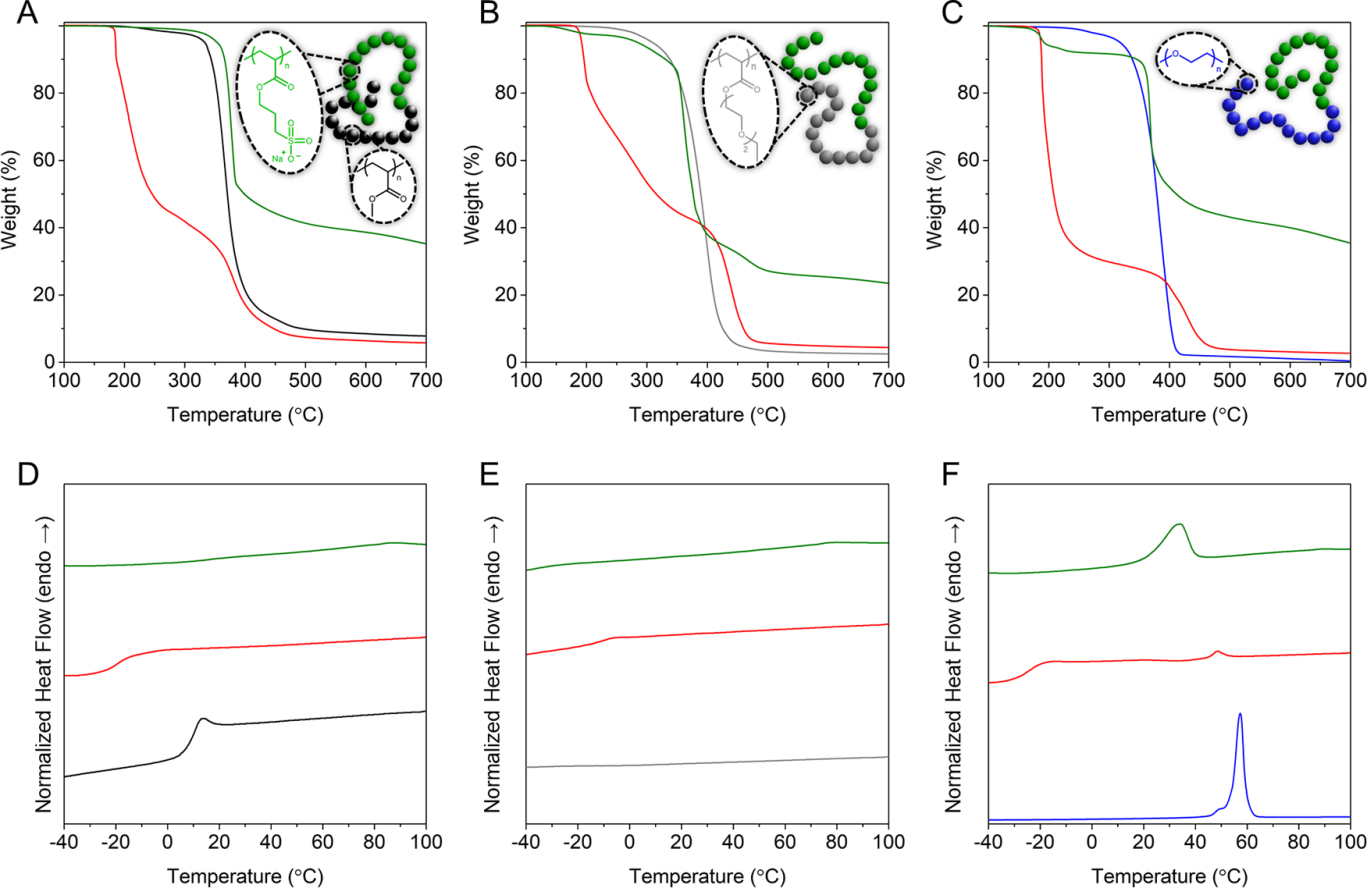
Thermogravimetric and differential scanning calorimetry analyses of the macroinitiators and their respective protected and NaI-deprotected block copolymers. (A) TGA and (D) DSC of PMA_92_ (black), PMA_92_-*b*-PBSPA_103_ (red), and PMA_92_-*b*-PSPA-Na_103_ (green). (B) TGA and (E) DSC of PDEGA_104_ (gray), PDEGA_104_-*b*-PBSPA_94_ (red), and PDEGA_104_-*b*-PSPA-Na_94_ (green). (C) TGA and DSC (F) of PEO_90_ (blue), PEO_90_-*b*-PBSPA_110_ (red), and PEO_90_-*b*-PSPA-Na_110_ (green).

Further thermal analyses were performed on the various protected and NaI-deprotected block copolymers (Figure 4). While all macroinitiators are stable up to ∼250 °C, the addition of PBSPA segments introduced the typical degradation pattern with a first weight loss at ∼190 °C due to the deterioration of the protective group and the subsequent thermolysis of the acrylic ester, while the remainder of the backbone decomposes at ∼400 °C. Once deprotected, the block copolymers are typically more thermally stable, and approximately 30–40% of the samples’ original weight remained at the end of the ramp. Note that early losses of mass are observed in deprotected BCPs due to the absorption of water that is difficult to fully remove, due to the highly hydroscopic nature of PSPA-Na as well as PDEGA and PEO blocks. Furthermore, characteristic temperatures (*e.g.*, *T*_g_ and *T*_m_) can be retrieved through DSC measurements on the block copolymers and their respective precursors. While both PBSPA_81_ and PMA_92_ homopolymers possess distinct glass-transition temperatures (*T*_g_ = −21.4 and 8.3 °C, respectively), PMA_92_-*b*-PBSPA_103_ exhibits a single one (*T*_g_ = −20.3 °C; Figure 4D). While this could indicate a single mixed phase,^57^ the value is much closer to the *T*_g_ of PBSPA homopolymer, in spite of an equimolar fraction of both blocks. More surprisingly, the amphiphilic BCP did not show any *T*_g_, although the PMA segment is left untouched by the nucleophilic deprotection. PDEGA_104_ on the contrary does not possess a detectable glass-transition temperature; therefore, only that of the PBSPA segment is visible in the block copolymers (*T*_g_ = −11.3 °C; Figure 4E), albeit slightly higher than that of PBSPA homopolymer. PEO_90_ is a semicrystalline polymer as evidenced by its melting temperature (*T*_m_ = 45.0 °C), which is also featured although shifted within the thermogram of PEO_92_-*b*-PBSPA_110_, along with the characteristic *T*_g_ of the PBSPA segment (Figure 4F). Upon removal of the isobutoxy groups, only the *T*_m_ remains, yet broadened and at a lower temperature. All characteristic temperatures and additional DSC and TGA thermograms of the EMIMI-deprotected block copolymers can be found in the Supporting Information (Figure S10).

### Block Copolymer Self-Assembly

**Figure 5:**
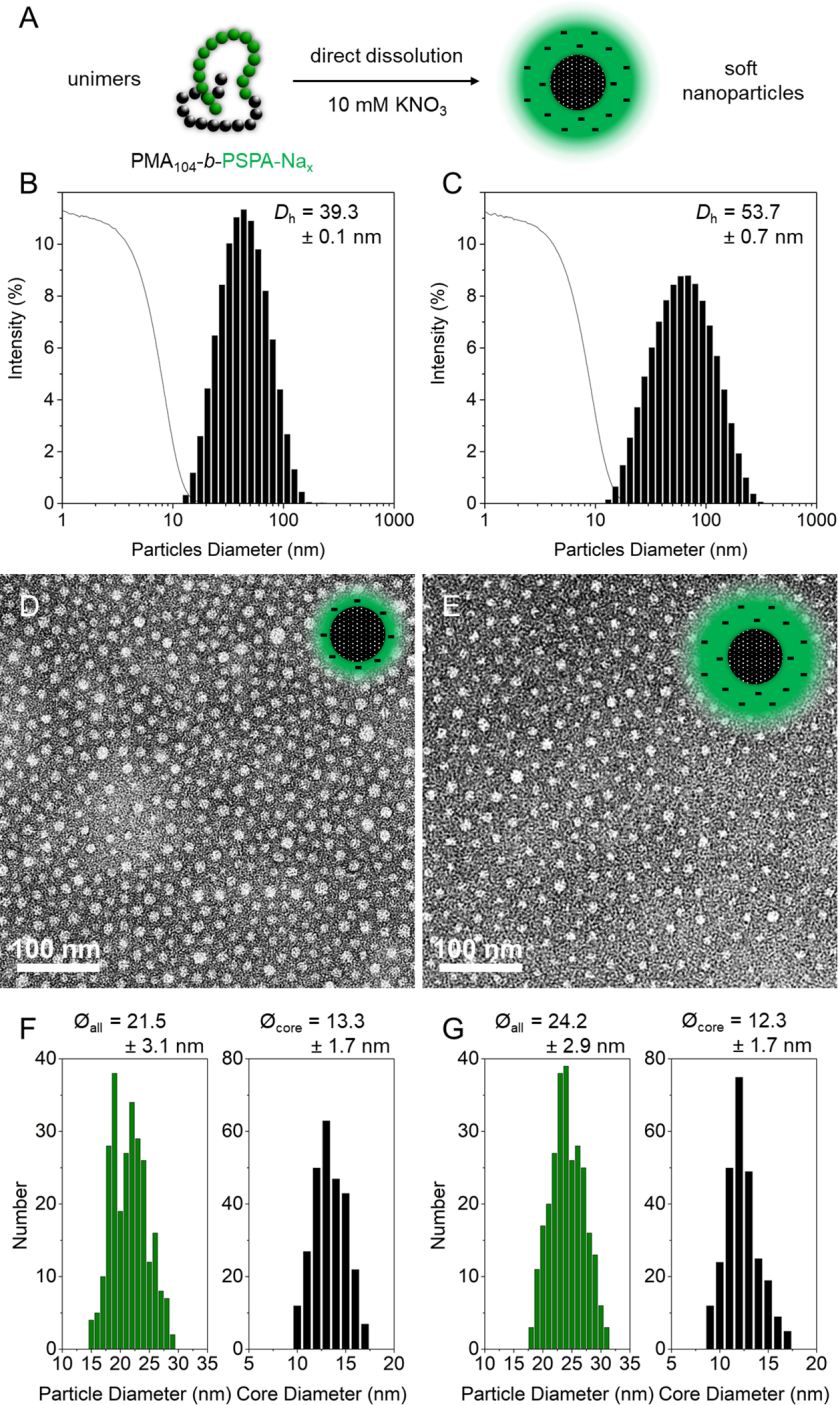
Solution self-assembly of PMA-based amphiphilic block copolymers. (A) The strong amphiphilic character of the PMA_104_-*b*-PSPA-Na*_x_* BCPs permits the formation of micelles through direct dissolution in aqueous medium. Dynamic light scattering intensity plots (bars) and corresponding correlogram functions (solid lines) of micelles achieved from (B) PMA_92_-*b*-PSPA-Na_103_ and (C) PMA_92_-*b*-PSPA-Na_231_. TEM images of uranyl acetate-stained (D) PMA_92_-*b*-PSPA-Na_103_- and (E) PMA_92_-*b*-PSPA-Na_231_-based micelles and (F, G) respective statistical analyses of particles.

With a range of block copolymers featuring various functionalities at hand, we explored the feasibility of their solution self-assembly. PMA-based block copolymers, with their amphiphilic character, were investigated first (Figure 5A). Solutions of each macromolecule were prepared at concentrations of *c* ∼ 1 g L^–1^ in 10 mM KNO_3_ through the “direct dissolution” method, *i.e.*, the aqueous solution was directly added into a vial containing an appropriate weight of the freeze-dried block copolymer. After prolonged stirring for a few days and gentle heating, the solutions were analyzed by dynamic light scattering (DLS; Figure 5B,C and Table 2). This brought valuable insights into the micelles’ size, with micelles produced from the self-assembly of the shorter BCP possessing a hydrodynamic diameter of *D*_h_ = 39.3 nm, while the larger BCP induced the formation of larger micelles with *D*_h_ = 53.7 nm. Both systems featured reasonably low polydispersity indices (PDIs) and both were established to have negative ζ-potential values, further confirming the charged nature of their shell. Due to the very soft nature of these acrylic micelles, atomic force microscopy (AFM) did not enable their visualization; hence, we invoked transmission electron microscopy (TEM; Figures 5D,E and S11). Samples were negatively stained with a 2 wt % uranyl acetate solution prior to imaging. As a result, the particles are closely packed, with their PMA cores left pristine and therefore less electron dense, while the charged PSPA-Na shells absorb the uranyl cations, strongly increasing their contrast. Noticeably, spherical micelles were observed for both samples, in spite of their different SPA-Na molar fractions (*x*_SPA-Na_ = 0.53 or 0.71), a phenomenon that has already been observed in the past.^51^ Statistical analyses on several TEM images (Figure 5F,G) revealed similar mean core diameters for both polymer systems (13.3 ± 1.7 *vs* 12.3 ± 1.7 nm), while micelles constructed from the larger PSPA-Na block possess larger overall (*i.e.*, core + shell) dimensions (24.2 ± 2.9 *vs* 21.5 ± 3.1 nm for the short ones). Note that the dimensions measured in TEM are lower than that measured in DLS, which can be attributed to drying and/or staining effects, with PSPA-Na chains being stretched in solution while partially collapsed on the TEM grid.

**Table 2:** Properties of the Micelles Achieved from the Self-Assembly of Amphiphilic PMA_92_-*b*-PSPA-Na*_x_*, Thermoresponsive PDEGA_104_-*b*-PSPA-Na*_y_* as well as Double-Hydrophilic PEO_90_-*b*-PSPA-Na*_z_* Block Copolymers

sample	*x*__SPA-Na__^a^	*D*__h__^b^ (nm)	PDI^b^	ζ (mV)
PMA__92__-*b*-PSPA-Na__103__	0.53	39.3 ± 0.1	0.225 ± 0.010	–24.5
PMA__92__-*b*-PSPA-Na__231__	0.71	53.7 ± 0.7	0.273 ± 0.010	–42.5
PDEGA__104__-*b*-PSPA-Na__94__	0.48	161.1 ± 3.0	0.270 ± 0.005	–46.9
PDEGA__104__-*b*-PSPA-Na__228__	0.69	198.5 ± 6.4	0.296 ± 0.016	–42.5
PEO__90__-*b*-PSPA-Na__110__ + P4VPq_119_	0.48	70.1 ± 0.3	0.082 ± 0.028	0.0
PEO__90__-*b*-PSPA-Na__237__ + P4VPq_119_	0.69	85.0 ± 0.6	0.037 ± 0.011	–6.6

a Determined by ^1^H NMR analysis.

b Determined by DLS in triplicate measurements.

**Figure 6:**
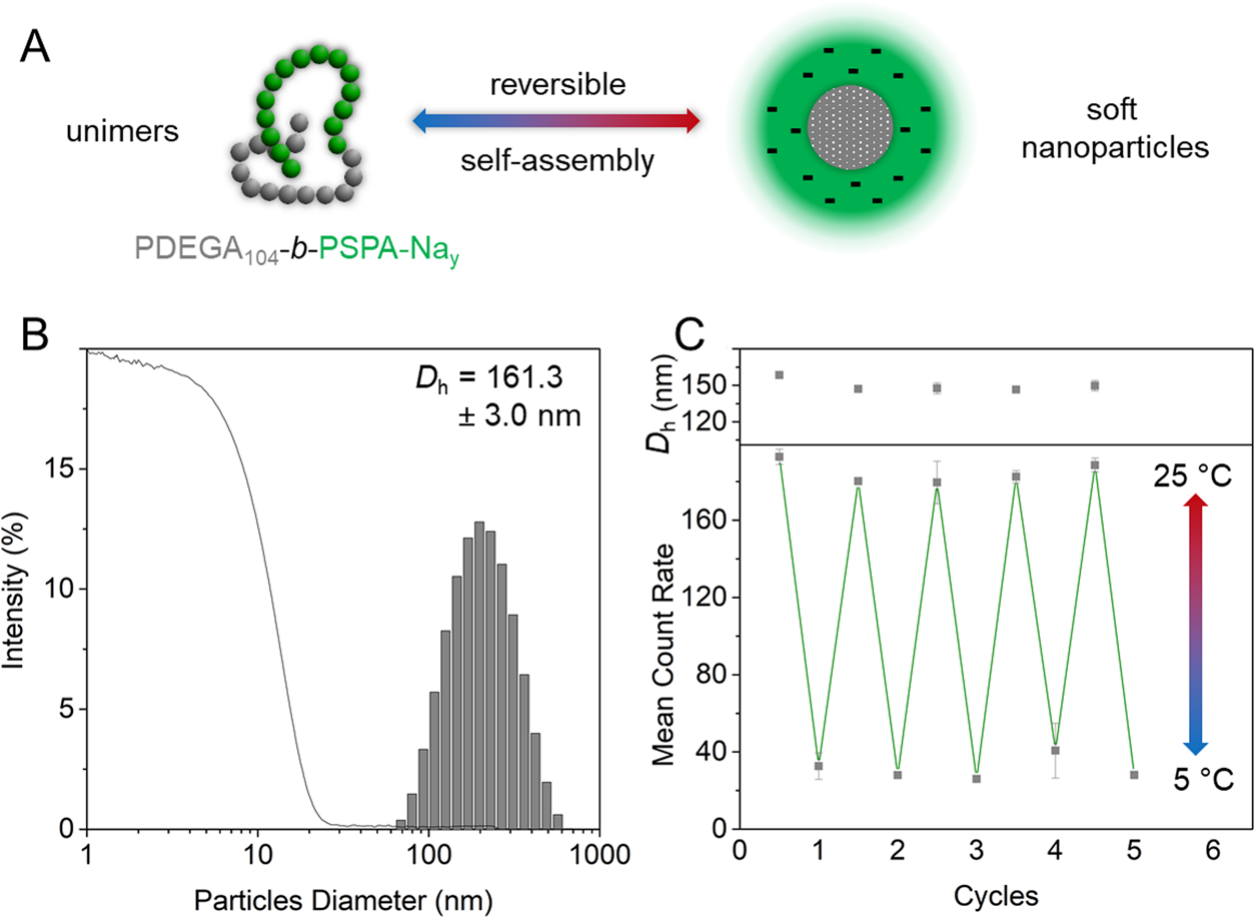
Reversible solution self-assembly of a thermoresponsive PDEGA-based block copolymer. (A) The PDEGA segment switches from hydrophilic to hydrophobic upon changing the temperature. (B) DLS intensity plot of micelles achieved from the direct dissolution of PDEGA_104_-*b*-PSPA-Na_94_ in 10 mM KNO_3_. (C) Cycling ability of the micelle formation upon heating and breakdown back into unimers upon cooling while achieving identical sizes upon reassembly.

While PMA remains water-insoluble under all conditions, the LCST of PDEGA enabled its reversible transition from hydrophilic (*T* < LCST) to hydrophobic (*T* > LCST; Figure 6A). This property was exploited to trigger the formation of soft micelles at temperatures above the LCST of their PDEGA block and their reversible disassembly upon cooling. DLS was used to measure the size of the resulting micelles, which, albeit larger than their PMA-based analogues (*D*_h_ = 161.3 and 198.5 nm for the short and long PSPA-Na segments, respectively; Figures 6B and S12-1), retained low polydispersity indices. This difference in size, while keeping a similar overall DP, could be explained by a rather swollen core in spite of a temperature above the LCST. While the LCST was first determined for a PDEGA homopolymer, it can also easily be obtained through DLS for the diblock (Figure S12-2), with a large drop of the mean count rate when cooling the polymer solution (*i.e.*, vast reduction of the number of micelles detected). Multiple cycles of heating and cooling (Figure 6C) evidenced the reversible character of the self-assembly mechanism, with similar mean count rates and average particle diameters obtained throughout the analysis.

**Figure 7:**
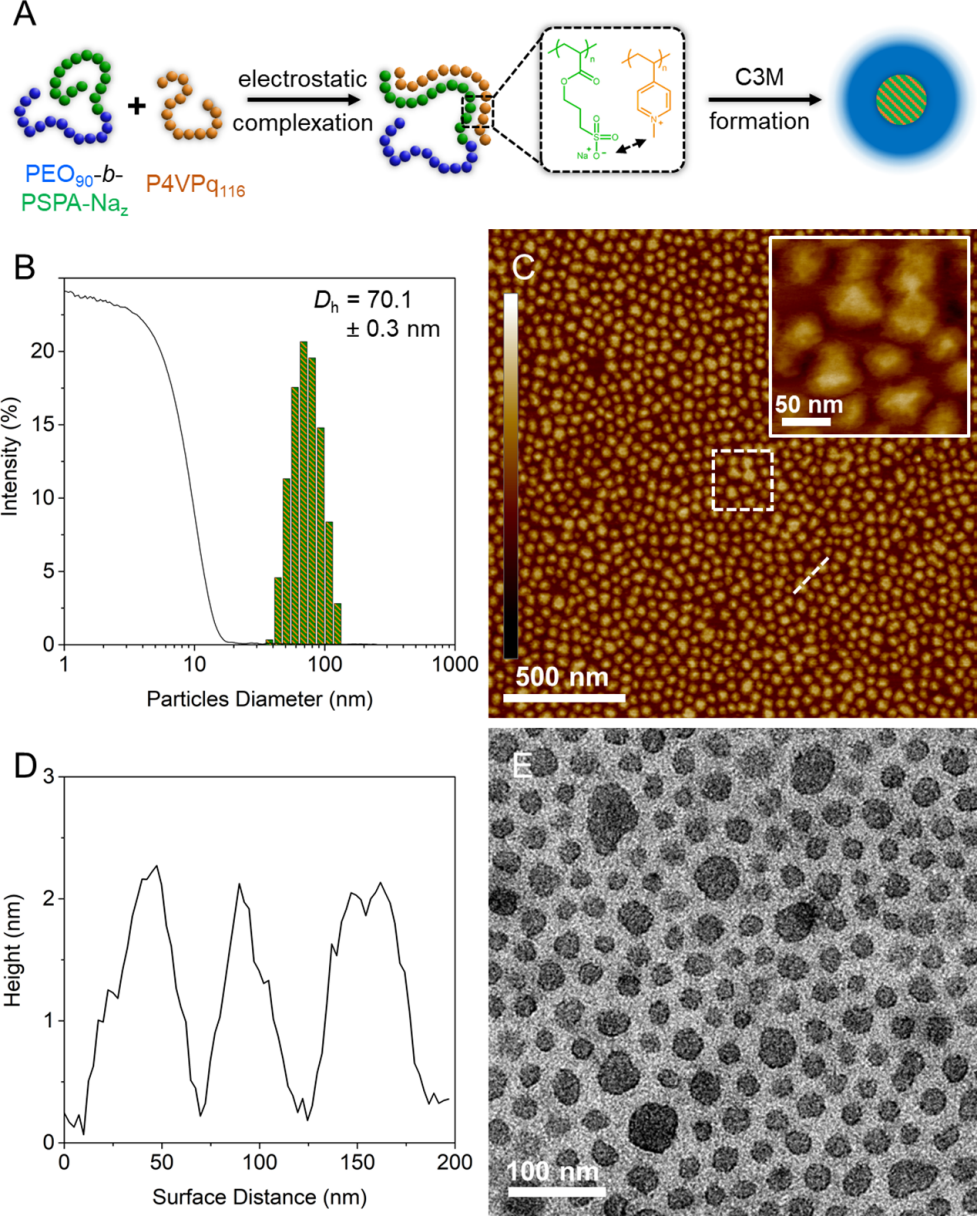
Formation of C3Ms using a PEO-based block copolymer. (A) The electrostatic interaction between a quaternized P4VP and the SPA-Na units of the BCP induces the formation of complex coacervate core micelles with a pseudo-hydrophobic domain stabilized by a charge-neutral PEO corona. (B) DLS intensity plot of micelles achieved from the complexation of PEO_90_-*b*-PSPA-Na_110_ to P4VPq_119_ in a 10 mM KNO_3_ solution. (C) AFM height images of the micelles deposited onto a freshly cleaved mica disk and (D) cross-sectional analysis across several particles. (E) TEM image of uranyl acetate-stained C3M. AFM *z*-scale is ± 2.5 nm.

Lastly, due to their double-hydrophilic nature, PEO_90_-*b*-PSPA-Na*_z_* block copolymers are incapable of spontaneous self-assembly in aqueous media. Nonetheless, the solubility of the charge-neutral PEO block enables their use for the formation and stabilization of complex coacervate core micelles (C3Ms; Figure 7A). Copolymers featuring a poly(ethylene glycol)-based segment and another charged moiety have been widely studied for such purposes and have already permitted the formation of multicompartment micelles^58,59^ and polymer nanowires.^6,7^ Herein, we utilize a poly(4-vinylpyridine) (P4VP) homopolymer produced through RAFT polymerization following an earlier reported procedure.^60^ The polymer was quaternized using iodomethane^61^ (P4VPq_119_) to introduce permanent charges and ensure pH-independent solubility. Detailed syntheses and characterizations can be found in the Supporting Information and Figure S13. Adequate volumes of PEO_92_-*b*-PSPA-Na_110_ or PEO_92_-*b*-PSPA-Na_237_ solutions at 1 g L^–1^ in 10 mM KNO_3_ were added to a 1 g L^–1^ solution of P4VPq_119_ and left to gently stir for a few minutes before analysis. We complexed the two components using nominally stoichiometric ratios of the negative and positive segments to achieve full charge compensation. DLS results (Figures 7B and S14 and Table 2) evidenced the formation of very narrowly dispersed micelles with mean diameters of *D*_h_ = 70.1 and 85.0 nm for the short and long PSPA-Na segments, respectively. The larger sizes compared to PMA-based analogues likely originate from the introduction of polymer material (*i.e.*, the P4VPq_119_ homopolymer) into the core of the micelles. Thanks to their more rigid core, these C3Ms could be deposited onto freshly cleaved mica disks and imaged by AFM (Figure 7C). Noticeably, the C3Ms obtained through the complexation of the larger PEO_92_-*b*-PSPA-Na_237_ possess larger dimensions compared to the shorter BCP (*h* ≈ 7.5 *vs* 2.3 nm and Ø ≈ 250 *vs* 50 nm; Figures 7D and S15), yet remained spherical in shape. While the corona-forming PEO segment remains identical, about a single P4VPq_116_ chain is required to compensate for the short PSPA-Na_110_ block but two are needed for the long PSPA-Na_237_ segment. This, in turn, strongly increases the overall size of the PEO_92_-*b*-PSPA-Na_237_-based C3Ms. Additional size artifacts, such as flattening of the soft particles onto the mica surface as well as tip deconvolution effects, might be at play. Lastly, TEM images (Figures 7E and S16) were recorded on the PEO-based micelles. Unlike previous samples, the uranyl acetate penetrated into the C3Ms and stained the particle cores, resulting in an inverted contrast (*i.e.*, “positive staining”). The particles are again closely packed, but their cores are noticeably much larger than that of similar PMA-based BCPs (*e.g.*, 13.3 ± 1.7 nm for the PMA__92__-*b*-PSPA-Na_103_*vs* 23.7 ± 3.7 nm for the PEO_92_-*b*-PSPA-Na_110_), a testimony of the presence of additional material (*i.e.*, P4VPq_116_). TEM analyses also confirmed the larger dimensions of C3Ms built from PEO_92_-*b*-PSPA-Na_237_ compared to its shorter analogue, with the requirement of twice as many P4VPq_116_ chains per PSPA-Na segment.

## Conclusions and Future Direction

Herein, we developed a facile route for the formation of sulfonate-containing strong polyanions *via* Cu(0)-RDRP. This technique permits the production of isobutoxy-protected sulfonate-based macromolecules under mild conditions (*i.e.*, at room temperature and using low concentrations of copper) and enables a high monomer conversion while keeping a low dispersity. The removal of the protective groups with a nucleophile, which can be chosen to tailor the properties of the resulting macromolecules, can be performed under mild conditions and enabled the facile production of strong polyanions. After the synthesis of several homopolymers with various degrees of polymerization (up to DP = 530, *M*_n NMR_ = 132 700 Da), a range of block copolymers (BCPs)—including one amphiphilic, one thermoresponsive, and one double-hydrophilic—were successfully produced through Cu(0)-RDRP of the isobutoxy-protected monomer from various macroinitiators. While the amphiphilic poly(methyl acrylate)-based BCPs can spontaneously self-assemble into spherical micelles in aqueous solution, the poly(di[ethylene glycol] ethyl ether acrylate)-based macromolecules are able to reversibly form micelles depending on the temperature of the medium. The last BCP type, based on poly(ethylene oxide), is double-hydrophilic and incapable of spontaneous self-assembly, yet can be employed for the formation and stabilization of narrowly dispersed complex coacervate core micelles upon complexation with an oppositely charged polymer (*i.e.*, polycation).

Our strategy for the production of BCPs with strong anionic segments is facile and efficient, uses mild conditions, and is compatible with a large variety of initiating systems. To improve upon our methodology even further, we have envisioned the *in situ* removal of the isobutoxy protective groups (*i.e.*, within the reaction vessel without prior purification; Figure S17), opening up an avenue toward the facile one-pot synthesis of strong amphiphiles.

## Supporting Information Available

Materials and methods, as well as additional ^1^H NMR spectra, SEC elugrams, DSC and TGA thermograms, DLS and UV–vis measurements, and AFM and TEM images (PDF)
