# Supplementary material for: Strong Anionic/Charge-Neutral Block Copolymers from Cu(0)-Mediated Reversible Deactivation Radical Polymerization
Source: Macromolecules. 2022 Sep 26;55(19):8795–807. doi: 10.1021/acs.macromol.2c01487 (PMC9558488; doi:10.1021/acs.macromol.2c01487)
Supplement: Supplementary file 1 — ma2c01487_si_001.pdf [file ma2c01487_si_001.pdf]

Strong Anionic/Charge-Neutral Block Copolymers from Cu(0)-Mediated Reversible Deactivation Radical Polymerization

Théophile Pelras*[a], Anton H. Hofman*[b], Lieke M. H. Germain[b], Anna M. C. Maan[b], Katja Loos*[a] and Marleen Kamperman[b]

[a]Macromolecular Chemistry and New Polymeric Materials, Zernike Institute for Advanced Materials, University of Groningen, Nijenborgh 4, 9747 AG, Groningen, The Netherlands.

[b]Polymer Science, Zernike Institute for Advanced Materials, University of Groningen, Nijenborgh 4, 9747 AG, Groningen, The Netherlands.

Supplementary Information

Corresponding authors:

theophile.pelras@rug.nl

a.h.hofman@rug.nl

k.u.loos@rug.nl

Table of Contents

Materials.......... 3

Characterisation Techniques.......... 4

Methods.......... 7

Supplementary Figures.......... 29

S1: NMR spectroscopy of the BSPA monomer.......... 29

S2: Kinetic analysis of the PBSPA homopolymerization.......... 30

S3: ^{1}H-NMR analysis on deprotected homopolymers.......... 31

S4: Thermal analyses on the homopolymers.......... 33

S5: Evaporation tests on MA and BSPA monomers.......... 36

S6: ^{1}H-NMR and SEC analyses of block copolymers.......... 37

S7: Determination of the LCST of PDEGA homopolymer.......... 39

S8: 'Deprotection' of macroinitiators (*i.e.* negative control).......... 40

S9: ^{1}H-NMR analysis on EMIMI-deprotected block copolymers.......... 42

S10: Thermal analyses on the EMIMI-deprotected block copolymers.......... 44

S1: Electron microscopy on the self-assembled PMA-based nanoparticles.......... 47

S12: Dynamic light scattering analysis on the PDEGA-based nanoparticles.......... 48

S13: Characterisation of the pristine and quaternised poly(4-vinylpyridine).......... 49

S14: Dynamic light scattering analysis on the long PEO-based C3Ms.......... 50

S15: Atomic force microscopy on the PEO-based C3Ms.......... 51

S16: Electron microscopy on the PEO-based C3Ms.......... 52

S17: One-pot synthesis of poly(3-sulfopropyl acrylate) sodium salt.......... 54

Supporting References.......... 55

Materials

Azobisisobutyronitrile (AIBN, 98 %), aluminium oxide (AlOx, basic, activated), benzoic acid (≥ 99.5 %) benzyl alcohol (BnOH, anhydrous 99.8 %), α-bromoisobutyryl bromide (B*i*BB, 98 %), copper(0) wire (1.0 mm, ≥ 99.9 %), copper(II) bromide ($CuBr_2$, 99%), *N,N*-dimethylformamide (DMF, 99+ anhydrous), 1,4-dioxane (99.8 %), ethyl α-bromoisobutyrate (EB*i*B. 98 %), iodomethane (MeI, ≥ 99.0 %), *L*-lactide (*L*-LA, 98 %), methyl acrylate (MA, 99 %), 3-sulfopropyl acrylate potassium salt (KSPA, %), triethylamine (TEA, ≥ 99.5 %), tris[2-(dimethylamino)ethyl]amine (Me_6-TREN, 97 %) and 4-vinylpyridine (4VP, 95 %) were purchased from Sigma-Aldrich. Dichloromethane (DCM, HPLC grade), diethyl ether (Et_2O, HPLC grade), ethyl acetate (EtOAc, HPLC grade), *n*-hexane (HPLC grade) and methanol (HPLC grade) were obtained from Macron Fine Chemicals. Hydrochloric acid solution (HCl, 37 %) and magnesium sulfate ($MgSO_4$, dried, extra pure) were sourced from Boom. Absolute ethanol (99.9 %) and dimethyl sulfoxide (DMSO, 99.9 %) were purchased from J.T. Baker. Di[ethylene glycol] ethyl ether acrylate (DEGA, > 98 %), poly(ethylene oxide) (PEO-OH, M_n = 4 000 Da) and oxalyl chloride ($(COCl)_2$, ≥ 98.0 %) were obtained from TCI. Silica gel for flash chromatography (40-63 µm) was sourced from Silicycle. Isobutanol (*i*BuOH, 99+ %), lithium bromide (LiBr, 99+ %, anhydrous) and sodium iodide (NaI, ACS reagent grade) were purchased from Acros Organics. Dry DCM was obtained through a MB-SPS 800 purification machine from MBraun, equipped with HPLC grade DCM from Ossum Chemicals. 2-Cyanopropan-2-yl propyl trithiocarbonate (CPP-TTC) was produced as reported elsewhere.[1]

AIBN was recrystallized twice from methanol. Commercially-available monomers were passed through a short AlOx column to remove inhibitors prior to polymerizations. All other chemicals were used as received.

Stirring bars used for SET-LRP were winded with copper wire (4 cm) and etched in HCl solution for 30 minutes followed by extensively washed with deionized water, ethanol and acetone prior to polymerizations.

Me_6-TREN/$CuBr_2$ stock solutions were prepared freshly prior polymerization by introducing 1 eq $CuBr_2$, 9 eq Me_6-TREN and DMSO into a glass vial and thorough mixing. A calculated volume of the stock solution was introduced into the reaction mixture so to obtain 0.01 eq $CuBr_2$ and 0.09 eq Me_6-TREN respective to the initiator or macroinitiator. An example of stock solution is as following: $CuBr_2$ (1 eq, 2.45 mg, 11.0 µmol), Me_6-TREN (9 eq, 22.5 mg, 97.8 µmol) and DMSO (10.14 mL).

Characterisation Techniques

Proton and carbon nuclear magnetic resonance (^{1}H-NMR and ^{13}C-NMR) spectra were recorded on an Agilent 400-MR 400 MHz spectrometer at 298 K. Deuterated chloroform ($CDCl_3$, 99.8 %), deuterated dimethyl sulfoxide (DMSO-d_6, 99.9 %), deuterated ethanol (ethanol-d_6, 99.5 %), deuterated methanol (methanol-d_4, 99.8 %) and deuterium oxide (D_2O, 99.9 %) were purchased from Sigma-Aldrich. Samples were dissolved in an appropriate solvent (≈ 5 g mL-1) and analyzed with a pulse width of 45 μs, spectral width of 12/-2 ppm, recycle delay of 1 s and either 32 or 128 scans (conversion or purified samples respectively). Spectra were analyzed with MestreNova software version 14.1.

Size-exclusion chromatography (SEC) was performed on a GPCMax system from Viscotek equipped with 302 TDA detectors array and two columns in series (PolarGel L and M, both 8 μm 30 cm) from Agilent Technologies. The columns and detectors were maintained at a temperature of 50 °C. DMF (≥ 99.9 %, Sigma-Aldrich) containing 0.01 M LiBr was used as eluent at a flow rate of 1 mL min^{-1}. Near monodisperse poly(methyl methacrylate) standards from Polymer Standard Services were used for the construction of a calibration curve. Samples were dissolved in the eluent at a concentration of ≈ 3 g L^{-1} and passed through a 0.45 μm PTFE filter prior to injection. Data acquisition and calculations were performed using Viscotek Omnisec software version 5.0.

Differential scanning calorimetry (DSC) measurements were recorded on a TA instruments DSCQ1000. The samples (~ 5 mg) were subjected to the following method: (i) equilibration at -80 °C, (ii) 5 min isotherm, (iii) ramp to 100 °C at 10 °C min^{-1}, (iv) 5 min isotherm, (v) ramp to -80 °C at 10 °C min^{-1}, (vi) 5 min isotherm and (vii) ramp to 100 °C at 10 °C min^{-1}. Data analysis was performed on the second heating cycle using TA Instruments TRIOS software.

Thermogravimetric analysis (TGA) measurements were recorded on a TA instruments TGA5500 analyzer. The samples (~ 5 mg) were subjected to a 20 min isotherm at 130 °C to remove traces of solvent and moisture before returning to room temperature and zeroing the balance. Then, the samples were heated from 30 °C to 700 °C at a rate of 10 °C min^{-1} under a continuous nitrogen flow. The data acquisition and analysis was done using TA Instruments TRIOS software.

Dynamic light scattering (DLS) measurements were performed on a Malvern Panalytical Zetasizer Ultra system, equipped with a helium-neon laser (λ = 633 nm) and an Avalanche Photodiode detector. Samples were prepared at a concentration of 1 g L^{-1} in triple-filtered (0.2

µm cellulose acetate) 10 mM KNO_3 solution. The nanoparticle solutions were measured at 25 °C in back scattering mode after 120 s equilibration time and using 30 cumulative recordings. The LCST of the homopolymer was determined by measurements at a controlled temperature between 5 and 25 °C in back scattering mode, after 120 s equilibration time, using 30 cumulative recordings and at a fixed attenuator value (determined from a preliminary scan at 25 °C). Samples were recorded in triplicates. Results were analyzed with ZS Xplorer software.

ζ-potential measurements were performed on a Malvern Panalytical Zetasizer ULtra system, equipped with a helium-neon laser (λ = 633 nm) and an Avalanche Photodiode detector. The measurements were taken at 25 °C while the acquisition times were determined automatically. Samples were recorded in triplicates.

UV-Vis spectroscopy measurements were performed on a Jasco V-650 spectrophotometer equipped with a PAC-743 Peltier cell. Polymer solutions were prepared at a concentration of 1 g L^{-1} in triple-filtered (0.2 µm cellulose acetate) 10 mM KNO_3 solution and cooled or heated by the Peltier cell per 1.0 ± 0.1 °C increments while being stirred at 200 rpm. Short transmittance spectra were recorded between 600 and 605 nm with 0.5 nm intervals and the value in that range was averaged. Samples were recorded in triplicates.

Transmission electron microscopy (TEM) imaging was performed on a Philips CM120 transmission electron microscope equipped with a tungsten filament and operated at an accelerating voltage of 120 kV. Images were acquired using a Gatan slow-scan CCD camera. Negatively stained specimen were prepared by deposition of 5 µL of the nanoparticle dispersion (c ~ 1 g L^{-1}) onto a glow-discharged (15 s at 50 mA and 300 V) 400-mesh copper grid with carbon support film and adsorption for 1 min before blotting. Before the specimen was fully dried, 5 µL of 2 wt.% uranyl acetate staining solution was deposited onto the grid, immediately blotted and a new 5 µL drop of staining solution was deposited and left to adsorb for 1 min before blotting. TEM images were analyzed using Image J software, using the software brightness and contrast correction tools to enhance the general quality of the snapshots and the software-imbedded measurement tool was utilized to determine the dimensions of the nanoparticles. The particles' diameter was measured center-to-center, *i.e.* from the center of one particle to the center of the neighboring one.

Atomic force microscopy (AFM) imaging was performed in standard tapping mode in air using a Bruker Dimension 3100 system, equipped with VTESPA-300 tapping mode cantilevers from Bruker. Freshly-prepared C3M samples (1 g L^{-1} in 10 mM KNO_3) were spin-coated (4 000 rpm, 60 s) onto a freshly-cleaved mica disc (Ø = 9.5 mm, muscovite mica grade V-1,

Proscitech) and measured on the same day. Images were processed with Bruker NanoScope software.

The formation of complex coacervate core micelles (C3M) was done just before analysis. Each polymer was dissolved in triple-filtered (0.2 µm cellulose acetate) 10 mM KNO_3 buffer at a concentration of 1 g L^{-1}. The 4VPq units account for 100 wt.% of the quaternised homopolymer (neglecting end groups), while the SPA-Na units account for 85 or 93 wt.% of the PEO_{90}-*b*-$PSPA\text{-}Na_{110}$ or PEO_{90}-*b*-$PSPA\text{-}Na_{237}$ block copolymers respectively. The volumes of block copolymer solutions was calculated to provide a 1:1 ratio between the 4VPq and the SPA-Na units to achieve full charge compensation. Therefore, a volume of 300 µL of P4VPq solution (*i.e*. 1.22 µmol 4VPq, 300 µg 4VP) was introduced in a dust-free vial equipped with a stirring bar, before 309 µL of PEO_{90}-*b*-$PSPA\text{-}Na_{110}$ (*i.e*. 309 µg BCP, 263 µg SPA-Na, 1.22 µmol SPA-Na) or 282 µL of PEO_{90}-*b*-$PSPA\text{-}Na_{237}$ (*i.e*. 282 µg BCP, 263 µg SPA-Na, 1.22 µmol SPA-Na) was added upon stirring (250 rpm). The solutions were left to stir for 10 min before analysis.

Methods

Synthesis of 3-isobutoxysulfopropyl acrylate (BSPA)

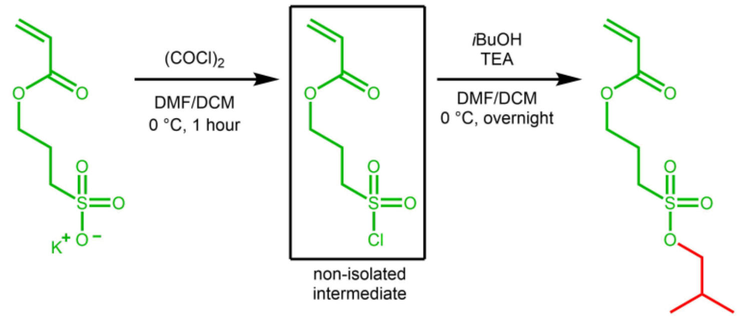

The synthesis of the monomer was adapted from a previously reported procedure.[1] KSPA (1 eq, 12.3 g, 52.9 mmol) and a stirring egg were charged in a 3-neck round bottom flask and subjected to several high vacuum / argon cycles to remove moisture before anhydrous DMF (40 mL) was added. The suspension was cooled over an ice bath before dropwise addition of $(COCl)_2$ (1.1 eq, 7.42 g, 58.5 mmol) in DCM (20 mL) under argon protection. In a separate round bottom flask, TEA (1.5 eq, 10.7 g, 106 mmol) and *i*BuOH (1.2 eq, 4.73 g, 63.8 mmol were dissolved in DCM (40 mL) and cooled over an ice bath. After an hour, the monomer/DMF/DCM solution was added to the second flask under argon protection using a dropping funnel, warmed-up to room temperature and left to stir overnight. Then, DI water (300 mL) was added and the reaction product was extracted with Et_2O (3x 200 mL). The organic phases were combined, concentrated in *vacuo* and washed with DI water (200 mL) and dried over $MgSO_4$. The monomer was further purified by flash column chromatography using *n*-hexane:EtOAc 4:1 as eluent ($R_f \approx 0.44$) to yield a light yellow liquid. Yield $_{\text{batch 1}}$: 10.1 g, 76 %. Yield $_{\text{batch 2}}$: 10.4 g, 80 %. ^{1}H-NMR (CDCl$_3$): (ppm) = 0.97 (d, 2 CH_3), 2.02 (m, CH), 2.23 (m, CH_2), 3.20 (t, CH_2), 3.99 (d, CH_2), 4.28 (t, CH2), 5.86 (d, CH), 6.11 (q, CH), 6.42 (d, CH). ^{13}C-NMR: (CDCl$_3$): (ppm) = 18.8, 23.5, 28.4, 47.3, 62.0, 75.7, 128.0, 131.6, 165.9.

Cu(0)-RDRP of poly(3-isobutoxysulfopropyl acrylate) homopolymers ($PBSPA_x$, x = 45, 81, 210, 370 or 530)

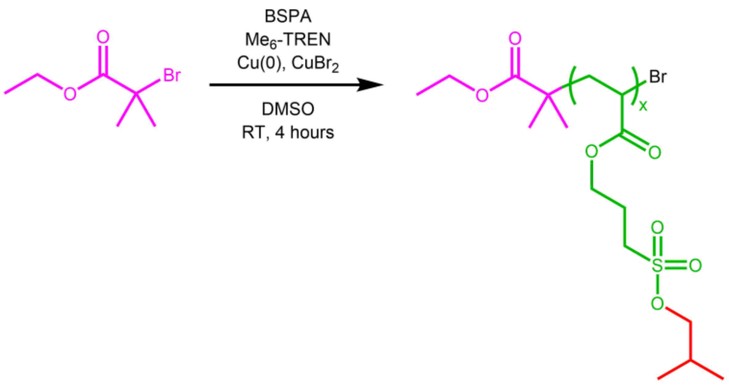

A typical Cu(0)-RDRP of 3-isobutoxysulfopropyl acrylate (polymerization of the protected monomer) is as following (here depicted for the synthesis of $PBSPA_{45}$): EB*i*B (1 eq, 10.5 mg, 53.9 µmol, 100 µL of stock solution of 321 mg EB*i*B in 3.07 mL DMSO), BSPA (48 eq, 648 mg, 2.59 mmol), Me_6-TREN (0.09 eq, 1.09 mg, 4.74 µmol, 262 µL of Me_6-TREN/$CuBr_2$ stock solution), $CuBr_2$ (0.01 eq, 0.117 mg, 0.525 µmol, 262 µL of Me_6-TREN/$CuBr_2$ stock solution), and DMSO (6 µL, total solvent volume: 200 eq, 363 µL) were charged in a Schlenk tube. A freshly-etched copper wire-winded stirring bar was introduced into the tube and kept above the liquid with a strong magnet before the tube was sealed with a rubber septum. The reaction mixture was deoxygenated with argon for 10 min before an aliquot was withdrawn under argon protection for ^{1}H-NMR sampling. The tube was left under a slight positive pressure and the stirring bar was dropped into the solution to start the polymerization. After 4 hours at room temperature, the copper wire-winded stirring bar was lifted above the liquid and the tube opened to air. After withdrawal of an aliquot for ^{1}H-NMR analysis, the reaction mixture was precipitated in cold 6:1 *n*-hexane:ethanol. The polymer was redissolved in excess THF, passed through a short AlOx column to remove excess copper, concentrated in vacuo and precipitated once more in cold 6:1 *n*-hexane:ethanol. The polymer was redissolved in minimal 1,4-dioxane and freeze-dried overnight to yield a transparent highly viscous liquid. Yield: 433 mg. ^{1}H-NMR: conversion = 93 %, DP_{NMR} = 45, $M_{n\ NMR}$ = 11 400 Da. SEC: $M_{n\ SEC}$ = 15 900 Da, Đ = 1.12.

All other Cu(0)-RDRP of BSPA were produced in a similar fashion, using the same amounts of BSPA and DMSO but lowering the amounts of EB*i*B, Me_6-TREN and $CuBr_2$ accordingly. Detailed compositions of the reactions are summarized in Table S1.

Table S1: Compositions of the reaction mixtures used for the Cu(0)-RDRP of BSPA.

Polymer	[EB*i*B]:[BSPA]:[Me_6-TREN]:[$CuBr_2$]	EB*i*B (μmol)	BSPA (mmol)	Me_6-TREN (μmol)	$CuBr_2$ (nmol)	DMSO (total, μL)	Yield (mg)
$PBSPA_{45}$	1:48:0.09:0.01	53.9	2.59	4.74	525	363	547
$PBSPA_{81}$	1:91:0.09:0.01	27.5	2.56	2.35	264	363	433
$PBSPA_{210}$	1:250:0.09:0.01	10.4	2.60	0.974	108	363	468
$PBSPA_{370}$	1:500:0.09:0.01	5.23	2.62	0.509	56.1	363	394
$PBSPA_{530}$	1:980:0.09:0.01	2.72	2.66	0.255	28.0	363	309

Deprotection of $PBSPA_x$ using NaI to produce poly(3-sulfopropyl acrylate) sodium salt ($PSPA\text{-}Na_x$, x = 45, 81, 210, 370 or 530)

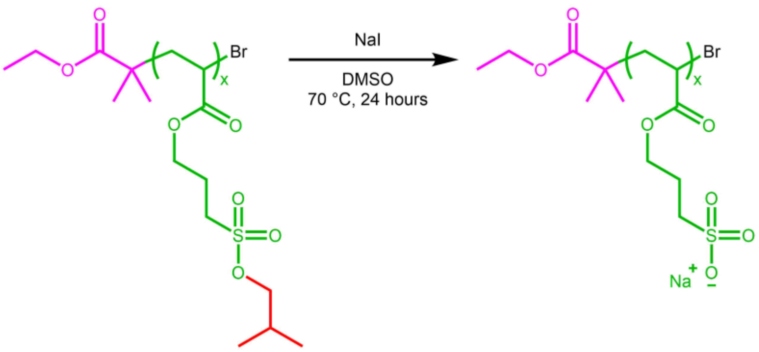

The deprotection of poly(3-isobutoxysulfopropyl acrylate) was performed as reported before[1] and a typical procedure is as following (here depicted for the synthesis of $PSPA\text{-}Na_{45}$): $PBSPA_{45}$ (1 eq, 108 mg, 432 µmol BSPA) and NaI (3 eq per BSPA unit, 212 mg, 1.42 mmol) were dissolved in DMSO (5 mL, 20 mg per mL) and charged into a glass vial equipped with a stirring bar. The reaction mixture was left to stir at 70 °C for 24 hours. The resulting dark brown solution was precipitated once in *n*-hexane:ethanol 1:2, redissolved in minimal DMSO and precipitated once more in *n*-hexane:ethanol 1:1 before a final wash with pure *n*-hexane. The polymer was redissolved in minimal DI water and freeze-dried overnight to yield an off-white brittle solid. Yield: 88 mg. ^{1}H-NMR: deprotection ≈ 100 %, $M_{n\ NMR}$ = 9 900 Da.

Table S2: Compositions of the reaction mixtures used for the nucleophilic deprotection of PBSPA homopolymers using NaI.

Polymer	PBSPA (mg)	BSPA units (µmol)	NaI (mmol)	Yield (mg)	$M_{n\ NMR}$ (Da)
$PSPA\text{-}Na_{45}$	108	432	1.42	89	9 900
$PSPA\text{-}Na_{81}$	103	412	1.24	88	17 700
$PSPA\text{-}Na_{210}$	104	416	1.39	88	45 600
$PSPA\text{-}Na_{370}$	74	296	1.15	62	80 100
$PSPA\text{-}Na_{530}$	60	240	0.967	59	114 700

Deprotection of PBSPA$_x$ with EMIMI to produce poly(3-sulfopropyl acrylate) 1-ethyl-3-methylimidazolium salt (PSPA-EMIM$_x$, x = 45, 81, 210, 370 or 530)

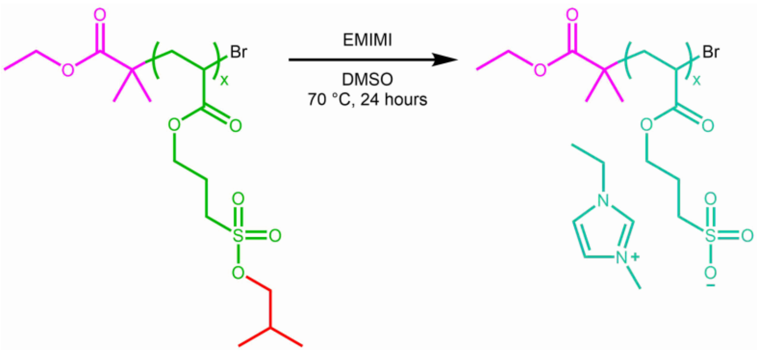

The deprotection of PBSPA$_x$ using EMIMI as nucleophile was performed similarly to that of NaI and a typical procedure is as following (here depicted for the synthesis of PSPA-EMIM$_{45}$): PBSPA$_{45}$ (1 eq, 108 mg, 432 µmol BSPA) and EMIMI (3 eq per BSPA unit, 301 mg, 1.26 mmol) were dissolved in DMSO (5 mL, 20 mg per mL) and charged into a glass vial equipped with a stirring bar. The reaction mixture was left to stir at 70 °C for 24 hours. The resulting dark brown solution was precipitated once in *n*-hexane:ethanol 6:1 and washed 4 times with *n*-hexane:ethanol 6:1 before a final wash with pure *n*-hexane. The polymer was redissolved in minimal DI water and freeze-dried overnight to yield a yellow and very gooey solid. Yield: 110 mg. ^{1}H-NMR: deprotection ≈ 100 %, $M_{n\ NMR}$ = 13 900 Da.

Table S3: Compositions of the reaction mixtures used for the nucleophilic deprotection of PBSPA homopolymers using EMIMI.

Polymer	PBSPA (mg)	BSPA units (µmol)	EMIMI (mmol)	Yield (mg)	$M_{n\ NMR}$ (Da)
PSPA-EMIM$_{45}$	108	432	1.27	110	13 900
PSPA- EMIM$_{81}$	103	412	1.29	98	24 800
PSPA- EMIM$_{210}$	102	408	1.27	79	64 000
PSPA- EMIM$_{370}$	80.1	320	1.09	57	112 700
PSPA- EMIM$_{530}$	72.2	288	1.00	65	161 300

Kinetic study on the Cu(0)-RDRP of 3-isobutoxysulfopropyl acrylate

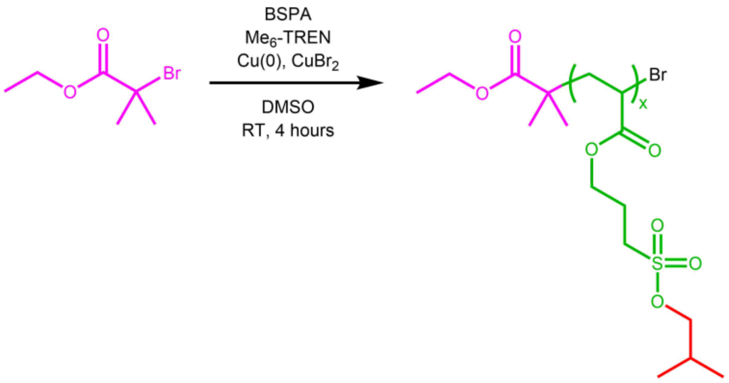

EB*i*B (1 eq, 5.35 mg, 27.4 µmol, 49.6 µL of stock solution of 321 mg EB*i*B in 4.07 mL DMSO), BSPA (94 eq, 640 mg, 2.56 mmol), Me_6-TREN (0.09 eq, 0.535 mg, 1.54 µmol, 41.5 µL of Me_6-TREN stock solution of 52.4 mg in 4.48 g DMSO), $CuBr_2$ (0.01 eq, 0.0572 mg, 0.257 µmol, 41.5 µL of $CuBr_2$ stock solution of 5.61 mg in 4.48 g DMSO), and DMSO (274 µL, total solvent volume: 200 eq, 363 µL) were charged in a Schlenk tube. A freshly-etched copper wire-winded stirring bar was introduced into the tube and kept above the liquid with a strong magnet before the tube was sealed with a rubber septum. The reaction mixture was deoxygenated with argon for 10 min before an aliquot was withdrawn under argon protection for ^{1}H-NMR sampling. The tube was left under slight positive pressure and the stirring bar was dropped to start the polymerization. Aliquots were withdrawn with a degassed syringe at preset time intervals under argon protection. ^{1}H-NMR samples (~ 2 drops) were directly diluted in $CDCl_3$ while SEC samples (~ 2 drops) were precipitated into cold 6:1 *n*-hexane:ethanol, dried in air and dissolved in eluent. After 4 hours at room temperature, the copper wire-winded stirring bar was lifted above the liquid and the tube was opened to air. The remainder of the reaction mixture was precipitated into cold 6:1 *n*-hexane:ethanol, redissolved in excess THF, passed through a short AlOx column to remove excess copper, concentrated in vacuo and precipitated once more in cold 6:1 *n*-hexane:ethanol. The polymer was redissolved in minimal 1,4-dioxane and freeze-dried overnight to yield a transparent highly viscous liquid. Yield: 323 mg. ^{1}H-NMR: conversion after 4 hours = 92 %, DP_{NMR} = 86, $M_{n\ NMR}$ = 21 700 Da. SEC: $M_{n\ SEC,\ purified}$ = 45 200 Da, $Đ_{SEC,\ purified}$ = 1.10.

Synthesis of poly(methyl acrylate) macroinitiator (PMA_{92})

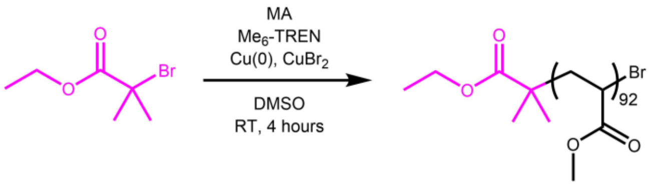

EB*i*B (1 eq, 45.2 mg, 232 µmol), MA (100 eq, 1.99 g, 23.1 mmol), Me_6-TREN (0.09 eq, 4.92 mg, 21.4 µmol, 420 µL of Me_6-TREN stock solution of 59.6 mg Me_6-TREN in 5.60 g DMSO), $CuBr_2$ (0.01 eq, 0.528 mg, 2.37 µmol, 420 µL of $CuBr_2$ stock solution of 6.4 mg $CuBr_2$ in 5.60 g DMSO), and DMSO (2.86 mL, total solvent volume: 200 eq, 3.27 mL) were charged in a Schlenk tube. A freshly-etched copper wire-winded stirring bar was introduced into the tube and kept above the liquid with a strong magnet before the tube was sealed with a rubber septum. The reaction mixture was deoxygenated via three freeze-pump-thaw cycles and backfilled with argon before an aliquot was withdrawn under argon protection for ^{1}H-NMR sampling. The tube was left under a slightly positive pressure and the stirring bar was dropped to start the polymerization. After reacting for 4 hours at room temperature, the copper wire-winded stirring bar was lifted above the liquid and the tube was opened to air. After withdrawal of an aliquot for ^{1}H-NMR sampling, the reaction mixture was precipitated into cold 6:1 *n*-hexane:ethanol. The polymer was redissolved in excess THF, passed through a short AlOx column to remove excess copper, concentrated in vacuo and precipitated once more in cold 6:1 *n*-hexane:ethanol. The polymer was redissolved in minimal 1,4-dioxane and freeze-dried overnight to yield a transparent highly viscous liquid. Yield: 1.75 g. ^{1}H-NMR: conversion = 92 %, DP_{NMR} = 92, $M_{n\,NMR}$ = 8 100 Da. SEC: $M_{n\,SEC}$ = 10 700 Da, Đ = 1.07.

Synthesis of poly(di[ethylene glycol] ethyl ether acrylate) macroinitiator (PDEGA$_{104}$)

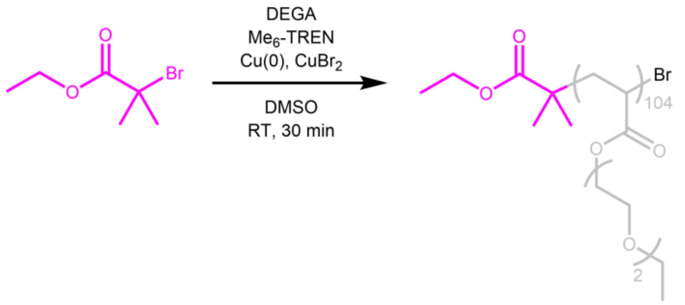

EB*i*B (1 eq, 10.4 mg, 53.3 µmol, 100 µL of stock solution), DEGA (193 eq, 1.94 g, 10.3 mmol), Me_6-TREN (0.09 eq, 1.07 mg, 4.65 µmol, 136 µL of Me_6-TREN stock solution of 40.0 mg Me_6-TREN in 5.62 g DMSO), $CuBr_2$ (0.01 eq, 0.115 mg, 0.516 µmol, 420 µL of $CuBr_2$ stock solution of 4.27 mg $CuBr_2$ in 5.62 g DMSO), and DMSO (2.67 mL, total solvent volume: 800 eq, 2.90 mL) were charged in a Schlenk tube. A freshly-etched copper wire-winded stirring bar was introduced into the tube and kept above the liquid with a strong magnet before the tube was sealed with a rubber septum. The reaction mixture was deoxygenated via three freeze-pump-thaw cycles and backfilled with argon before an aliquot was withdrawn under argon protection for ^{1}H-NMR sampling. The tube was left under a slightly positive pressure and the stirring bar was dropped to start the polymerization. After reacting for 30 min at room temperature, the copper wire-winded stirring bar was lifted above the liquid and the tube was opened to air. After withdrawal of an aliquot for ^{1}H-NMR analysis, the reaction mixture was precipitated into cold 6:1 *n*-hexane:ethanol. The polymer was redissolved in excess THF, passed through a short AlOx column to remove excess copper, concentrated *in vacuo* and precipitated once more in cold 6:1 *n*-hexane:ethanol. The polymer was redissolved in minimal 1,4-dioxane and freeze-dried overnight to yield a transparent highly viscous liquid. Yield: 929 mg. ^{1}H-NMR: conversion = 54 %, DP $_{NMR}$ = 104, $M_{n\ NMR}$ = 19 700 Da. SEC: $M_{n\ SEC}$ = 22 000 Da, Đ = 1.11.

Synthesis of poly(ethylene oxide) macroinitiator (PEO_{90}-Br)

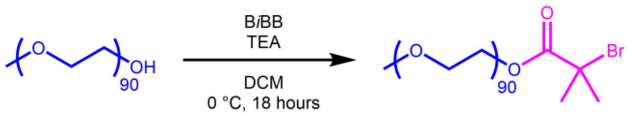

Poly(ethylene oxide) was modified via esterification of the hydroxy group by treating the polymer with an excess acid halide, a method adapted from an earlier reported procedure.[2] PEO_{90}-OH (1 eq, 25 g, 6.25 mmol) was charged into a three-neck 250 mL round bottom flask equipped with a stirring egg and subjected to several high-vacuum/argon cycles to remove residual moisture. Then, 100 mL dry DCM and TEA (1.5 eq, 947 mg, 1304 µL, 9.38 mmol) were added and the mixture was cooled over an ice bath before dropwise addition of B*i*BB (5 eq, 7188 mg, 3865 µL, 31.3 mmol) in 50 mL dry DCM over argon protection. The reaction mixture was allowed to warm up to room temperature and left to stir overnight before addition of 1 mL ethanol to quench unreacted B*i*BB. The solution was concentrated *in vacuo* and precipitated once in cold *n*-hexane. The polymer was recrystallised following a reported procedure,[3] where the yellowish precipitate was redissolved in 200 mL hot ethanol (50 °C), cooled down in the fridge at 4 °C and centrifuged. The yellow supernatant was removed and the operation was repeated several times until the supernatant remained completely colourless. The polymer was finally dried in a vacuum oven to obtain a white powder. Yield: 22.5 g. ^{1}H-NMR: $M_{n\ NMR}$ = 4 100 Da, functionalisation ≈ 100 %. SEC: $M_{n\ SEC}$ = 7 000 Da, Đ = 1.06.

‘Deprotection’ of macroinitiators (*i.e.* negative control experiments)

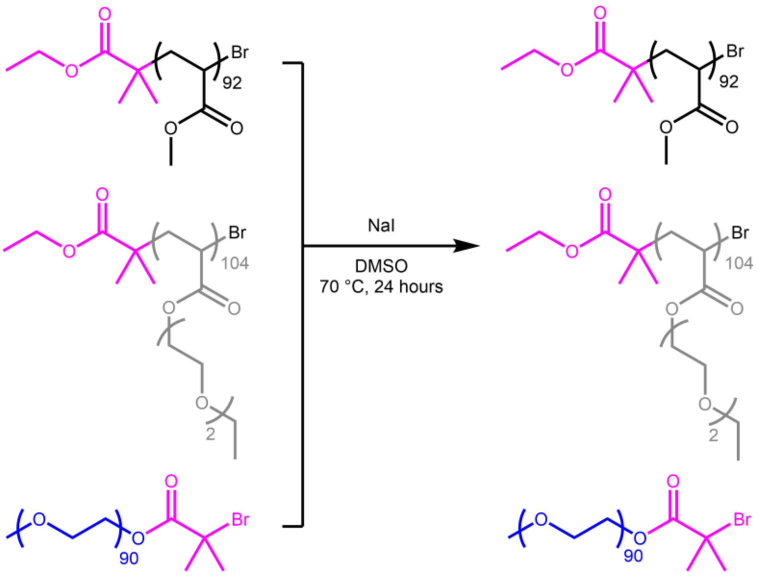

PMA_{92} (1 eq, 101 mg, 1.17 mmol MA) and NaI (3 eq per MA unit, 529 mg, 3.53 mmol) were dissolved in DMSO (5 mL, 20 mg per mL) and charged into a glass vial equipped with a stirring bar. The reaction mixture was left to stir at 70 °C for 24 hours. The resulting yellow solution was precipitated once in *n*-hexane:ethanol 6:1, redissolved in minimal THF and precipitated once more in *n*-hexane:ethanol 6:1. The polymer was redissolved in minimal 1,4-dioxane and freeze-dried overnight to yield a light yellow highly viscous liquid. Yield: 77 mg. SEC: $M_{n\ SEC}$ = 10 500 Da, Đ = 1.10.

PEO_{90}-Br (1 eq, 102 mg, 2.32 mmol ethylene oxide) and NaI (3 eq per MA unit, 1.04 g, 6.93 mmol) were dissolved in DMSO (5 mL) and charged into a glass vial equipped with a stirring bar. The reaction mixture was left to stir at 70 °C for 24 hours. The resulting yellow solution was precipitated in *n*-hexane:ethanol 6:1 and washed three times with *n*-hexane:ethanol 6:1. The polymer was redissolved in minimal 1,4-dioxane and freeze-dried overnight to yield a light yellow powder. Yield: 20 mg. SEC: $M_{n\ SEC}$ = 7 100 Da, Đ = 1.07.

$PDEGA_{104}$ (1 eq, 99.7 mg, 530 µmol di[ethylene glycol] ethyl ether acrylate) and NaI (3 eq per DEGA unit, 248 mg, 1.65 mmol) were dissolved in DMSO (5 mL) and charged into a glass vial equipped with a stirring bar. The reaction mixture was left to stir at 70 °C for 24 hours. The resulting yellow solution was dialysed against acetone and concentrated *in vacuo*. The polymer was then redissolved in minimal 1,4-dioxane and freeze-dried overnight to yield a light yellow viscous oil. Yield: 90.2 mg. SEC: $M_{n\ SEC}$ = 22 100 Da, Đ = 1.13.

Synthesis of poly(methyl acrylate)-*block*-poly(3-isobutoxysulfopropyl acrylate) (PMA_{92}-*b*-$PBSPA_x$, x = 103 or 231)

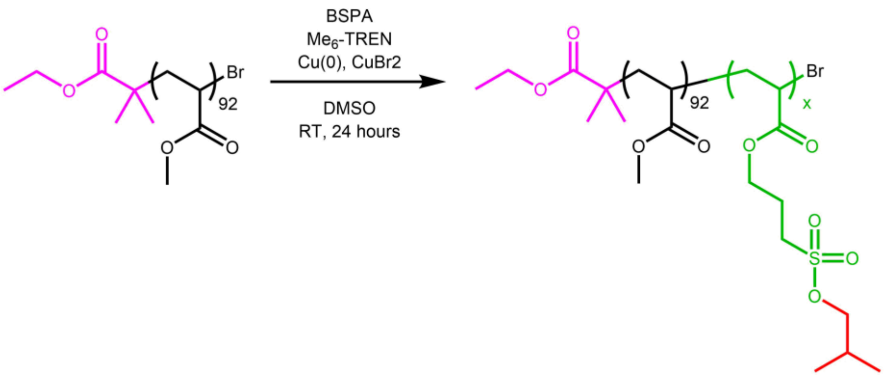

For PMA_{92}-*b*-$PBSPA_{103}$, PMA_{92} (1 eq, 101.7 mg, 12.6 µmol), BSPA (99 eq, 311 mg, 1.24 mmol), Me_6-TREN (0.09 eq, 0.264 mg, 1.15 µmol, 70.5 mg of a stock solution of 41.9 mg Me_6-TREN in 11.2 g DMSO) and $CuBr_2$ (0.01 eq, 0.0281 mg, 0.126 µmol, 70.5 mg of a stock solution of 4.45 mg $CuBr_2$ in 11.2 g DMSO) and DMSO (438 µL, total solvent volume of 500 µL) were charged in a Schlenk tube. A freshly-etched copper wire-winded stirring bar was introduced into the tube and kept above the liquid with a strong magnet before the tube was sealed with a rubber septum. The reaction mixture was deoxygenated with argon for 5 min before an aliquot was withdrawn under argon protection for ^{1}H-NMR sampling. The tube was left under slight positive pressure and the stirring bar was dropped to start the polymerization. After 24 hours at room temperature, the copper wire-winded stirring bar was lifted above the liquid and the tube was opened to air. After withdrawal of an aliquot for ^{1}H-NMR analysis, the reaction mixture was precipitated into cold 6:1 *n*-hexane:ethanol. The polymer was redissolved in THF, passed through a short AlOx column to remove excess copper, concentrated in vacuo and precipitated once more in cold 6:1 *n*-hexane:ethanol. The polymer was redissolved in minimal 1,4-dioxane and freeze-dried overnight to yield a transparent highly viscous liquid. Yield: 325 mg. ^{1}H-NMR: conversion = 94 %, $DP_{PBSPA, NMR}$ = 103, $M_{n\ NMR}$ = 33 900 Da. SEC: $M_{n\ SEC}$ = 52 500 Da, Đ = 1.13.

PMA_{92}-*b*-$PBSPA_{231}$ was prepared following the above-described procedure but using PMA_{92} (1 eq, 101.6 mg, 12.5 µmol), BSPA (250 eq, 784 mg, 3.13 mmol), Me_6-TREN (0.09 eq, 0.260 mg, 1.13 µmol, 69.3 mg of a stock solution of 41.9 mg Me_6-TREN in 11.2 g DMSO) and $CuBr_2$ (0.01 eq, 0.0276 mg, 0.124 µmol, 69.3 mg of a stock solution of 4.45 mg $CuBr_2$ in 11.2 g DMSO) and DMSO (438 µL, total solvent volume of 500 µL). Yield: 639 mg. ^{1}H-NMR: conversion = 82 %, $DP_{PBSPA, NMR}$ = 231, $M_{n\ NMR}$ = 65 600 Da. SEC: $M_{n\ SEC}$ = 89 200 Da, Đ =1.24.

Synthesis of poly(methyl acrylate)-*block*-poly(3-sulfopropyl acrylate) sodium salt block copolymers (PMA$_{92}$-*b*-PSPA-Na$_{X}$, x = 103 or 231)

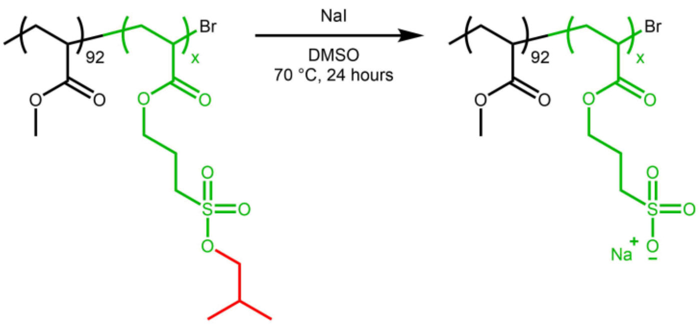

For PMA$_{92}$-*b*-PSPA-Na$_{103}$, PMA$_{92}$-*b*-PBSPA$_{103}$ (105 mg, 1 eq BSPA, 79.8 mg BSPA, 319 μmol BSPA), NaI (3 eq to BSPA, 144 mg, 958 μmol) and DMSO (2 mL) were charged into a glass vial equipped with a stirring bar. The polymer and the salt were dissolved at room temperature before the reaction mixture was heated at 70 °C for 24 hours. The polymer was isolated by precipitation into *n*-hexane:ethanol 1:1 once, washed 3 times with *n*-hexane:ethanol 1:1 before a final wash with pure *n*-hexane. The polymer was then dissolved in minimal mixture of 1,4-dioxane and DI water and freeze-dried to yield an off-white solid. Yield: 80 mg. ^{1}H-NMR: deprotection ≈ 100 %, $M_{n\ NMR}$ = 30 300 Da, $x_{SPA\text{-}Na}$ = 53 mol.%.

PMA$_{92}$-*b*-PSPA-Na$_{231}$ was prepared following the above-described procedure but using PMA$_{92}$-*b*-PBSPA$_{231}$ (111 mg, 1 eq BSPA, 97.7 mg BSPA, 391 μmol BSPA), NaI (3 eq to BSPA, 176 mg, 1.17 mmol) and DMSO (2 mL). Yield: 80 mg. ^{1}H-NMR: deprotection ≈ 100 %, $M_{n\ NMR}$ = 58 000 Da, $x_{SPA\text{-}Na}$ = 72 mol.%.

Synthesis of poly(methyl acrylate)-*block*-poly(3-sulfopropyl acrylate) 1-ethyl-3-methylimidazolium salt block copolymers (PMA$_{92}$-*b*-PSPA-EMIM$_{X}$, x = 103 or 231)

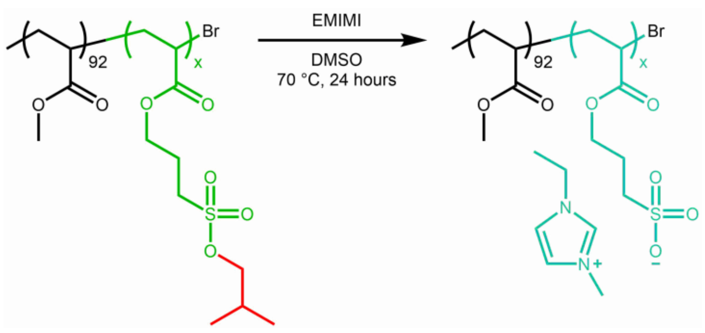

For PMA$_{92}$-*b*-PSPA-EMIM$_{103}$, PMA$_{92}$-*b*-PBSPA$_{103}$ (94.0 mg, 1 eq BSPA, 71.4 mg BSPA, 286 µmol BSPA), EMIMI (3 eq to BSPA, 236 mg, 992 µmol) and DMSO (2 mL) were charged into a glass vial equipped with a stirring bar. The polymer and the salt were dissolved at room temperature before the reaction mixture was heated at 70 °C for 24 hours. The polymer was isolated by precipitation into *n*-hexane:ethanol 6:1 once, washed 3 times with *n*-hexane:ethanol 1:1 before a final wash with pure *n*-hexane. The polymer was then dissolved in minimal mixture of 1,4-dioxane and DI water and freeze-dried to yield a light yellow highly viscous liquid. Yield: 117 mg. ^{1}H-NMR: deprotection ≈ 100 %, $M_{n\ NMR}$ = 39 400 Da, $x_{SPA\text{-}EMIM}$ = 53 mol.%.

PMA$_{92}$-*b*-PSPA-EMIM$_{231}$ was prepared following the above-described procedure but using PMA$_{92}$-*b*-PBSPA$_{231}$ (99.4 mg, 1 eq BSPA, 87.5 mg BSPA, 350 µmol BSPA), EMIMI (3 eq to BSPA, 273 mg, 1.15 mmol) and DMSO (2 mL). Yield: 128 mg. ^{1}H-NMR: deprotection ≈ 100 %, $M_{n\ NMR}$ = 78 300 Da, $x_{SPA\text{-}EMIM}$ = 72 mol.%.

Synthesis of poly(di[ethylene glycol] ethyl ether acrylate)-*block*-poly(3-isobutoxysulfopropyl acrylate) (PDEGA$_{104}$-*b*-PBSPA$_y$, y = 94 or 228)

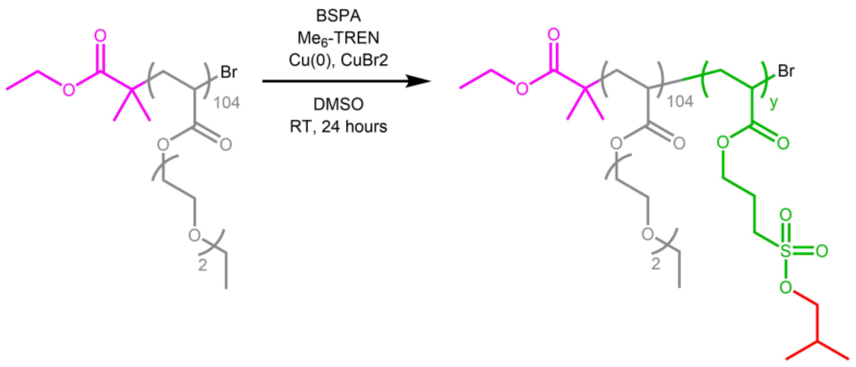

For PDEGA$_{104}$-*b*-PBSPA$_{94}$, PDEGA$_{104}$ (1 eq, 244 mg, 12.4 µmol), BSPA (99 eq, 306 mg, 1.22 mmol), Me$_6$-TREN (0.09 eq, 0.272 mg, 1.18 µmol, 124 mg of a stock solution of 24.4 mg Me$_6$-TREN in 11.2 g DMSO) and $CuBr_2$ (0.01 eq, 0.0293 mg, 0.131 µmol, 113 mg of a stock solution of 2.63 mg in 11.2 g DMSO) and DMSO (438 µL, total solvent volume of 500 µL) were charged in a Schlenk tube. A freshly-etched copper wire-winded stirring bar was introduced into the tube and kept above the liquid with a strong magnet before the tube was sealed with a rubber septum. The reaction mixture was deoxygenated with argon for 5 min before an aliquot was withdrawn under argon protection for ^{1}H-NMR sampling. The tube was left under slight positive pressure and the stirring bar was dropped into the solution to start the polymerization. After 24 hours at room temperature, the copper wire-winded stirring bar was lifted above the liquid and the tube opened to air. After withdrawal of an aliquot for ^{1}H-NMR sampling, the reaction mixture was precipitated in cold 6:1 *n*-hexane:ethanol. The polymer was redissolved in THF, passed through a short AlOx column to remove excess copper, concentrated in vacuo and precipitated once more in cold 6:1 *n*-hexane:ethanol. The polymer was redissolved in minimal 1,4-dioxane and freeze-dried overnight to yield a transparent highly viscous liquid. Yield: 422 mg. ^{1}H-NMR: conversion = 85 %, DP $_{\text{PBSPA, NMR}}$ = 94, $M_{n\ \text{NMR}}$ = 43 200 Da. SEC: $M_{n\ \text{SEC}}$ = 48 100 Da, Đ = 1.25.

PDEGA$_{104}$-*b*-PBSPA$_{228}$ was prepared following the above-detailed procedure but using PDEGA$_{104}$ (1 eq, 244 mg, 12.4 µmol), BSPA (249 eq, 774 mg, 3.10 mmol), Me$_6$-TREN (0.09 eq, 0.260 mg, 1.13 µmol, 119 mg of a stock solution of 24.4 mg Me$_6$-TREN in 11.2 g DMSO) and $CuBr_2$ (0.01 eq, 0.0280 mg, 0.126 µmol, 119 mg of a stock solution of 2.63 mg in 11.2 g DMSO) and DMSO (438 µL, total solvent volume of 500 µL). Yield: 766 mg. ^{1}H-NMR: conversion = 84 %, DP $_{\text{PBSPA, NMR}}$ = 228, $M_{n\ \text{NMR}}$ = 76 700 Da. SEC: $M_{n\ \text{SEC}}$ = 81 700 Da, Đ = 1.37.

Synthesis of poly(di[ethylene glycol] ethyl ether acrylate)-*block*-poly(3-sulfopropyl acrylate) sodium salt block copolymers (PDEGA$_{104}$-*b*-PSPA-Na$_{x}$, x = 94 or 228)

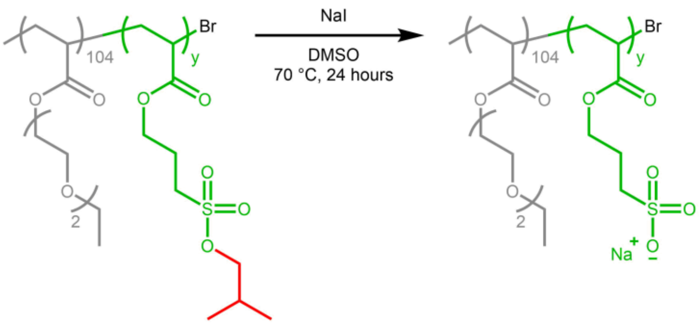

For PDEGA$_{104}$-*b*-PSPA-Na$_{94}$, PMA$_{104}$-*b*-PBSPA$_{94}$ (105 mg, 1 eq BSPA, 56.7 mg BSPA, 227 µmol BSPA), NaI (3 eq to BSPA, 113 mg, 753 µmol) and DMSO (2 mL) were charged into a glass vial equipped with a stirring bar. The polymer and the salt were dissolved at room temperature before the reaction mixture was heated at 70 °C for 24 hours. The polymer was isolated by precipitation into *n*-hexane:ethanol 1:1 once, washed 3 times with *n*-hexane:ethanol 1:1 before a final wash with pure *n*-hexane. The polymer was then dissolved in a minimal amount of a mixture of 1,4-dioxane and DI water and freeze-dried to yield an off-white solid. Yield: 102 mg. ^{1}H-NMR: deprotection ≈ 100 %, $M_{n\ NMR}$ = 40 000 Da, $x_{SPA\text{-}Na}$ = 48 mol.%.

PDEGA$_{104}$-*b*-PSPA-Na$_{228}$ was prepared following the above-detailed procedure but using PDEGA$_{104}$-*b*-PBSPA$_{228}$ (112 mg, 1 eq BSPA, 82.9 mg BSPA, 332 µmol BSPA), NaI (3 eq to BSPA, 164 mg, 1.09 mmol) and DMSO (2 mL). Yield: 101 mg. ^{1}H-NMR: deprotection ≈ 100 %, $M_{n\ NMR}$ = 68 900 Da, $x_{SPA\text{-}Na}$ = 69 mol.%.

Synthesis of poly(di[ethylene glycol] ethyl ether acrylate)-*block*-poly(3-sulfopropyl acrylate) 1-ethyl-3-methylimidazolium salt block copolymers (PDEGA$_{104}$-*b*-PSPA-EMIM$_x$, x = 94 or 228)

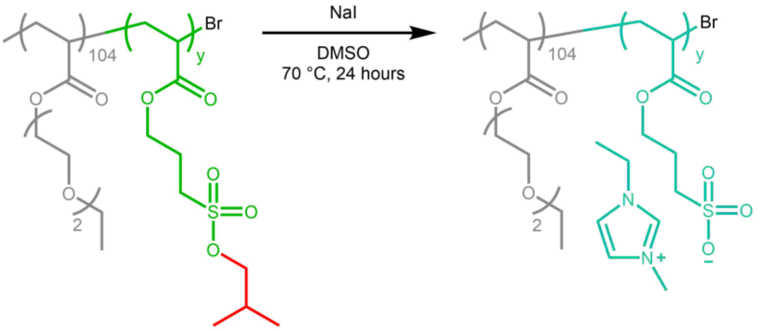

For PDEGA$_{104}$-*b*-PSPA-EMIM$_{94}$, PMA$_{104}$-*b*-PBSPA$_{94}$ (104 mg, 1 eq BSPA, 56.2 mg BSPA, 225 μmol BSPA), EMIMI (3 eq to BSPA, 201 mg, 704 μmol) and DMSO (2 mL) were charged into a glass vial equipped with a stirring bar. The polymer and the salt were dissolved at room temperature before the reaction mixture was heated at 70 °C for 24 hours. The polymer was isolated by precipitation into *n*-hexane:ethanol 1:1 once, washed 3 times with *n*-hexane:ethanol 1:1 before a final wash with pure *n*-hexane. The polymer was then dissolved in a minimal amount of a mixture of 1,4-dioxane and DI water and freeze-dried to yield a light yellow highly viscous liquid. Yield: 125 mg. ^{1}H-NMR: deprotection ≈ 100 %, $M_{n\ NMR}$ = 48 300 Da, $x_{SPA\text{-}Na}$ = 48 mol.%.

PDEGA$_{104}$-*b*-PSPA-EMIM$_{228}$ was prepared following the above-detailed procedure but using PDEGA$_{104}$-*b*-PBSPA$_{228}$ (106 mg, 1 eq BSPA, 78.4 mg BSPA, 314 μmol BSPA), EMIMI (3 eq to BSPA, 244 mg, 853 μmol) and DMSO (2 mL). Yield: 146 mg. ^{1}H-NMR: deprotection ≈ 100 %, $M_{n\ NMR}$ = 89 000 Da, $x_{SPA\text{-}Na}$ = 69 mol.%.

Synthesis of short poly(ethylene oxide)-*block*-poly(3-isobutoxysulfopropyl acrylate) (PEO_{90}-*b*-$PBSPA_y$, y = 110 or 237)

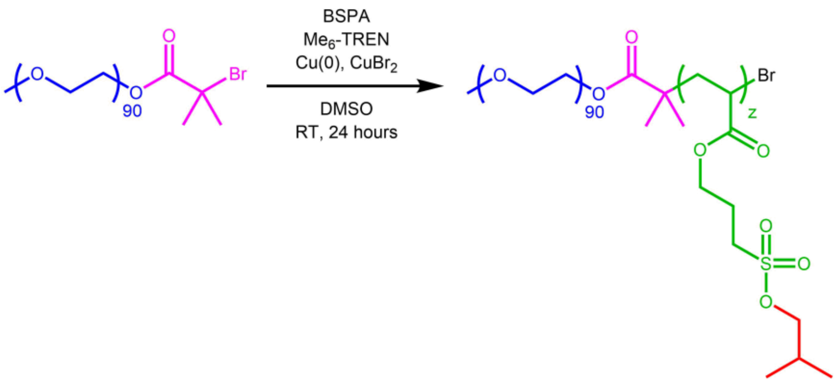

For PEO_{90}-*b*-$PBSPA_{110}$, PEO_{90}-Br (1 eq, 50.4 mg, 12.3 µmol), BSPA (104 eq, 320 mg, 1.28 mmol), Me_6-TREN (0.09 eq, 0.266 mg, 1.16 µmol, 89.6 mg of a stock solution 33.3 mg in 11.2 g DMSO) and $CuBr_2$ (0.01 eq, 0.0286 mg, 0.128 µmol, 89.6 mg of a stock solution 3.58 mg in 11.2 g DMSO) and DMSO (422 µL, total solvent volume of 500 µL) were charged in a Schlenk tube. A freshly-etched copper wire-winded stirring bar was introduced into the tube and kept above the liquid with a strong magnet before the tube was sealed with a rubber septum. The reaction mixture was deoxygenated with argon for 5 min before an aliquot was withdrawn under argon protection for ^{1}H-NMR sampling. The tube was left under slight positive pressure and the stirring bar was dropped to start the polymerization. After 24 hours at room temperature, the copper wire-winded stirring bar was lifted above the liquid and the tube opened to air. After withdrawal of an aliquot for ^{1}H-NMR sampling, the reaction mixture was precipitated in cold 6:1 *n*-hexane:ethanol. The polymer was redissolved in THF, passed through a short AlOx column to remove excess copper, concentrated *in vacuo* and precipitated once more in cold 6:1 *n*-hexane:ethanol. The polymer was redissolved in minimal 1,4-dioxane and freeze-dried overnight to yield a transparent highly viscous liquid. Yield: 289 mg. ^{1}H-NMR: conversion = 97 %, $DP_{PBSPA, NMR}$ = 110, $M_{n\ NMR}$ = 31 600 Da. SEC: $M_{n\ SEC}$ = 44 400 Da, Đ = 113.

PEO_{90}-*b*-$PBSPA_{237}$ was prepared following the above-detailed procedure but using PEO_{90}-Br (1 eq, 50.0 mg, 12.4 µmol), BSPA (102 eq, 316.7 mg, 1.27 mmol), Me_6-TREN (0.09 eq, 0.256 mg, 1.11 µmol, 86.2 mg of a stock solution 33.3 mg in 11.2 g DMSO) and $CuBr_2$ (0.01 eq, 0.0276 mg, 0.124 µmol, 86.2 mg of a stock solution 3.58 mg in 11.2 g DMSO) and DMSO (922 µL, total solvent volume of 1000 µL). Yield: 626 mg. ^{1}H-NMR: conversion = 85 %, $DP_{PBSPA, NMR}$ = 237, $M_{n\ NMR}$ = 63 400 Da. SEC: $M_{n\ SEC}$ = 76 700 Da, Đ = 1.21.

Synthesis of poly(ethylene oxide)-*block*-poly(3-sulfopropyl acrylate) sodium salt block copolymers (PEO_{90}-*b*-PSPA-Na_z, z = 110 or 237)

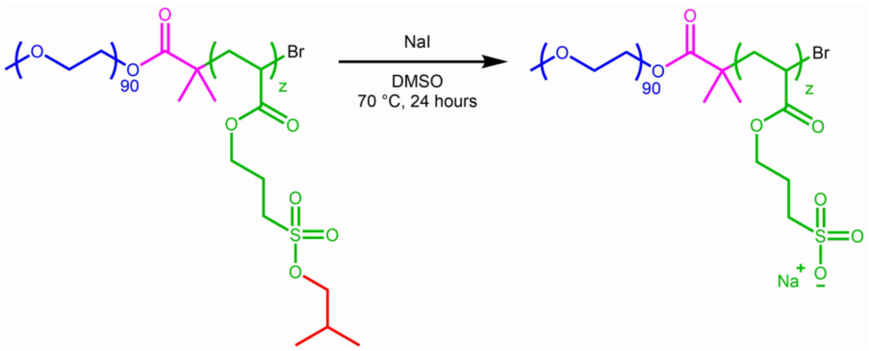

For PEO_{90}-*b*-PSPA-Na_{110}, PEO_{90}-*b*-$PBSPA_{110}$ (108 mg, 1 eq BSPA, 94.0 mg BSPA, 376 µmol BSPA), NaI (3 eq to BSPA, 169 mg, 1.13 mmol) and DMSO (2 mL) were charged into a glass vial equipped with a stirring bar. The polymer and the salt were dissolved at room temperature before the reaction mixture was heated at 70 °C for 24 hours. The polymer was isolated by precipitation into cold *n*-hexane:ethanol 1:1 once, washed 3 times with cold *n*-hexane:ethanol 1:1 before a final wash with pure *n*-hexane. The polymer was then redissolved in a minimal DI water and freeze-dried to yield an off-white solid. Yield: 99 mg. ^{1}H-NMR: deprotection ≈ 100 %, $M_{n\ NMR}$ = 27 900 Da, $x_{SPA\text{-}Na}$ = 55 mol.%.

PEO_{90}-*b*-PSPA-Na_{237} was prepared following the above-detailed procedure but using PEO_{90}-*b*-$PBSPA_{237}$ (101 mg, 1 eq BSPA, 94.4 mg BSPA, 380 µmol BSPA), NaI (3 eq to BSPA, 171 mg, 1.14 mmol) and DMSO (2 mL). Yield: 90 mg. ^{1}H-NMR: deprotection ≈ 100 %, $M_{n\ NMR}$ = 55 300 Da, $x_{SPA\text{-}Na}$ = 73 mol.%.

Synthesis of poly(ethylene oxide)-*block*-poly(3-sulfopropyl acrylate) 1-ethyl-3-methylimidazolium salt block copolymers (PEO$_{90}$-*b*-PSPA-EMIM$_{X}$, x = 110 or 237)

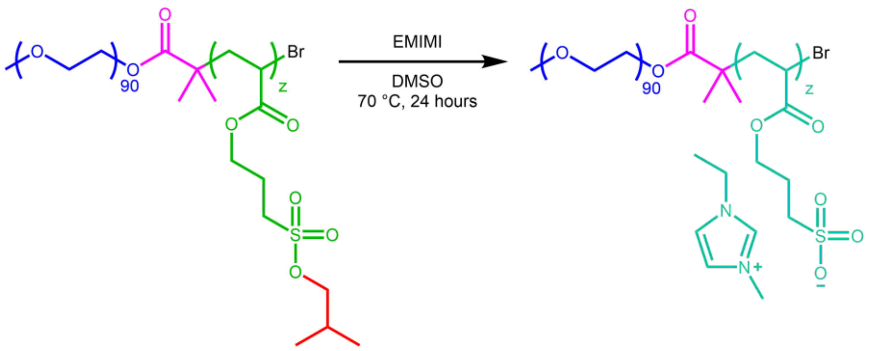

For PEO$_{90}$-*b*-PSPA-EMIM$_{110}$, PEO$_{90}$-*b*-PBSPA$_{110}$ (97.0 mg, 1 eq BSPA, 84.4 mg BSPA, 338 µmol BSPA), EMIMI (3 eq to BSPA, 257 mg, 1.08 mmol) and DMSO (2 mL) were charged into a glass vial equipped with a stirring bar. The polymer and the salt were dissolved at room temperature before the reaction mixture was heated at 70 °C for 24 hours. The polymer was isolated by precipitation into *n*-hexane:ethanol 6:1 once, washed 3 times with *n*-hexane:ethanol 1:1 before a final wash with pure *n*-hexane. The polymer was then dissolved in a minimal amount of a mixture of 1,4-dioxane and DI water and freeze-dried to yield a light yellow highly viscous liquid. Yield: 125 mg. ^{1}H-NMR: deprotection ≈ 100 %, $M_{n\ NMR}$ = 37 500 Da, $x_{SPA\text{-}EMIM}$ = 53 mol.%.

PEO$_{90}$-*b*-PSPA-EMIM$_{237}$ was prepared following the above-detailed procedure but using PEO$_{90}$-*b*-PBSPA$_{237}$ (99.7 mg, 1 eq BSPA, 93.7 mg BSPA, 3750 µmol BSPA), EMIMI (3 eq to BSPA, 273 mg, 1.15 mmol) and DMSO (2 mL). Yield: 121 mg. ^{1}H-NMR: deprotection ≈ 100 %, $M_{n\ NMR}$ = 76 100 Da, $x_{SPA\text{-}EMIM}$ = 53 mol.%.

Synthesis of quaternised poly(4-vinylpyridine) ($P4VPq_{119}$)

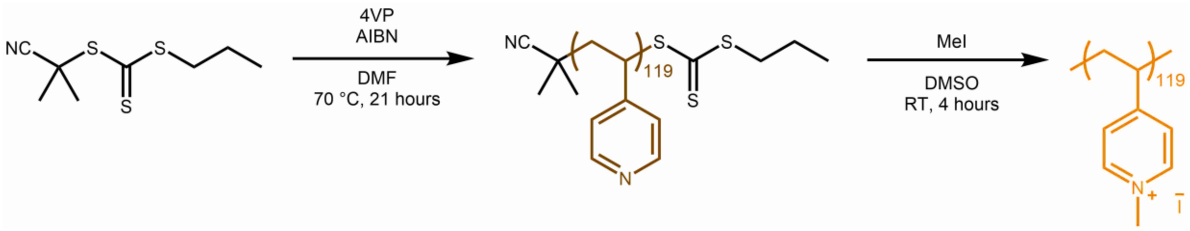

P4VP was produced following a previously reported procedure.[4] Here, CPP-TCC (1 eq, 108 mg, 493 µmol), 4VP (195 eq, 10.1 g, 96.2 mmol), AIBN (0.07 eq, 5.4 mg, 33 µmol) and DMF (13.8 mL) were charged into a round bottom flask equipped with a stirring egg. The reaction mixture was deoxygenated with argon for 45 min before being immersed in a pre-heated oil bath at 70 °C. After 21 hours, the vessel was removed from the oil bath and cooled down over ice and the rubber septum was opened to quench the reaction. The polymer was isolated by precipitation into toluene, redissolved in DMF and precipitated once more into toluene to yield a yellow solid. Yield: 5.51 g ^{1}H-NMR: conversion = 61 %, $DP_{P4VP, NMR}$ = 119, $M_{n\ NMR}$ = 12 700 Da. SEC: $M_{n\ SEC}$ = 10 400 Da, Đ = 1.17.

P4VP was quaternised according to an earlier-reported procedure.[5] Here, P4VP (2.06 g, 1 eq 4VP, 19.6 mmol 4VP) and 20 mL DMSO were charged in a round bottom flask equipped with a stirring egg before MeI (2 eq to 4VP, 5.56 g, 39.2 mmol) was added and the flask was left to stir for 4 hours at room temperature. The quaternised polymer was isolated by precipitation into acetone and dried *in vacuo* to yield a light orange solid. Yield: 4.62 g. ^{1}H-NMR: degree of quaternisation > 99 %, $M_{n\ NMR}$ = 29 600 Da.

One-pot synthesis of poly(sulfopropyl acrylate) sodium salt (PSPA-Na_{54})

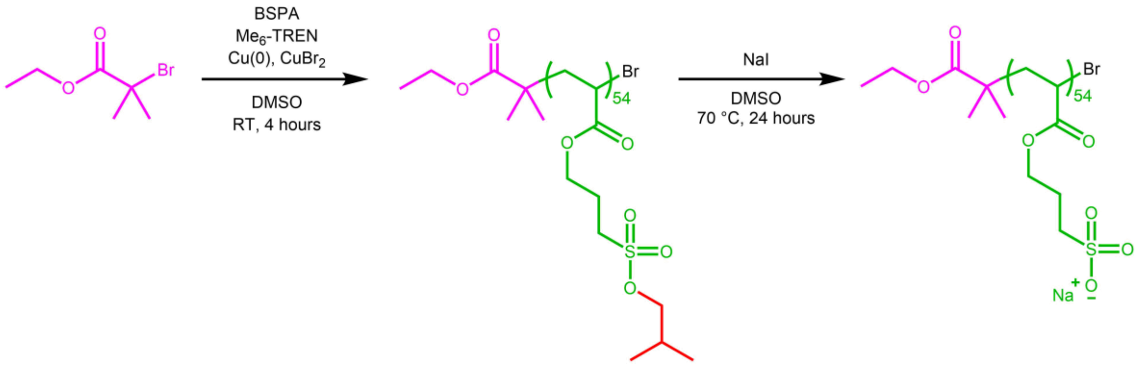

EB*i*B (1 eq, 5.81 mg, 29.8 µmol, 53.7 µL of stock solution at 321 mg EB*i*B in 4.07 mL DMSO), BSPA (88 eq, 653 mg, 2.61 mmol), Me_6-TREN (0.09 eq, 0.556 mg, 2.42 µmol, 164 µL of Me_6-TREN stock solution of 33.3 mg Me_6-TREN in 11.2 g DMSO), $CuBr_2$ (0.01 eq, 0.0598 mg, 0.268 µmol, 164 µL of $CuBr_2$ stock solution of 3.58 mg $CuBr_2$ in 11.2 g DMSO), and DMSO (153 µL, total solvent volume: 200 eq, 363 µL) were charged in a Schlenk tube. A freshly-etched copper wire-winded stirring bar was introduced into the tube and kept above the liquid with a strong magnet before the tube was sealed with a rubber septum. The reaction mixture was deoxygenated with argon for 10 min before an aliquot was withdrawn under argon protection for ^{1}H-NMR sampling. The tube was left under slight positive pressure and the stirring bar was dropped into the reaction mixture to start the polymerization. After 4 hours at room temperature, the copper wire-winded stirring bar was lifted above the liquid and the tube was opened to air. One drop of the mixture was withdrawn for ^{1}H-NMR sampling and two more drops were precipitated in cold 6:1 *n*-hexane:ethanol to isolate a PBSPA sample. Then, NaI (3 eq per BSPA unit, 1.17 g, 7.80 mmol) were dissolved in DMSO (10 mL) and charged into the Schlenk tube along with a new copper-free stirring bar. The vessel was immersed in a pre-heated oil bath and the reaction mixture was left to stir at 70 °C for 24 hours. The resulting dark brown solution was precipitated once in *n*-hexane:ethanol 1:2, redissolved in minimal DMSO and precipitated once more in *n*-hexane:ethanol 1:1 before a final wash with *n*-hexane. The polymer was redissolved in minimal DI water and freeze-dried overnight to yield an off-white brittle solid. Yield: 247 mg. ^{1}H-NMR: conversion = 61 %, DP_{NMR} = 54, $M_{n\ NMR\ PBSPA}$ = 13 700 Da, deprotection ≈ 100 %, $M_{n\ NMR\ PSPA}$ = 11 900 Da. SEC: $M_{n\ PBSPA}$ = 27 000 Da, $Đ_{PBSPA}$ = 1.16.

Table S4: Characteristics of the PSPA-Na based block copolymers and their respective precursors synthesized for this study.

	x_{SPA}‡ (mol. %)	$M_{n\ NMR}$‡ (Da)	$M_{n\ SEC}$≠ (Da)	Đ≠
PMA_{92}		8 100	10 700	1.07
PMA_{92}-*b*-$PBSPA_{103}$		33 900	52 500	1.13
PMA_{92}-*b*-PSPA-Na_{103}	53	30 300	n.a.	n.a.
PMA_{92}-*b*-$PBSPA_{231}$		65 600	89 200	1.24
PMA_{92}-*b*-PSPA-Na_{231}	72	58 000	n.a.	n.a.
PEO_{90}-Br		4 100	7 000	1.06
PEO_{90}-*b*-$PBSPA_{110}$		31 600	44 400	1.13
PEO_{90}-*b*-PSPA-Na_{110}	55	27 900	n.a.	n.a.
PEO_{90}-*b*-$PBSPA_{237}$		63 400	76 700	1.21
PEO_{90}-*b*-PSPA-Na_{237}	73	55 300	n.a.	n.a.
$PDEGA_{104}$		19 700	22 000	1.11
$PDEGA_{104}$-*b*-$PBSPA_{94}$		43 200	48 100	1.25
$PDEGA_{104}$-*b*-PSPA-Na_{94}	48	40 000	n.a.	n.a.
$PDEGA_{104}$-*b*-$PBSPA_{228}$		76 700	81 700	1.37
$PDEGA_{104}$-*b*-PSPA-Na_{228}	69	68 900	n.a.	n.a.

‡ determined from a combination of ^{1}H-NMR conversion samples and end-group analysis; ≠ determined from SEC data in DMF with 0.01 M LiBr and calibrated against near-monodisperse PMMA standards.

Supplementary Figures

S1: NMR spectroscopy of the BSPA monomer

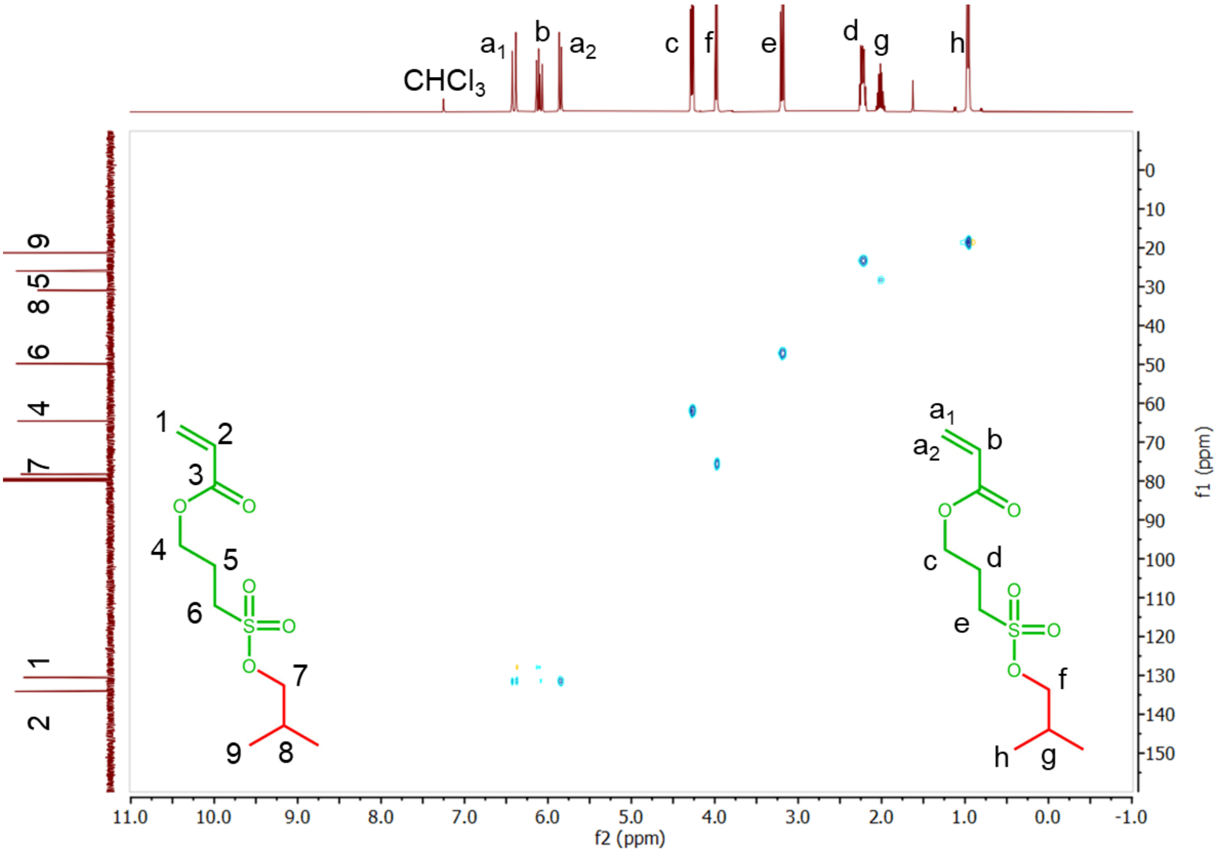

Figure S1: Heteronuclear single quantum coherence (HSQC) spectrum of 3-isobutoxysulfopropyl acrylate, recorded in $CDCl_3$.

S2: Kinetic analysis of the PBSPA homopolymerization

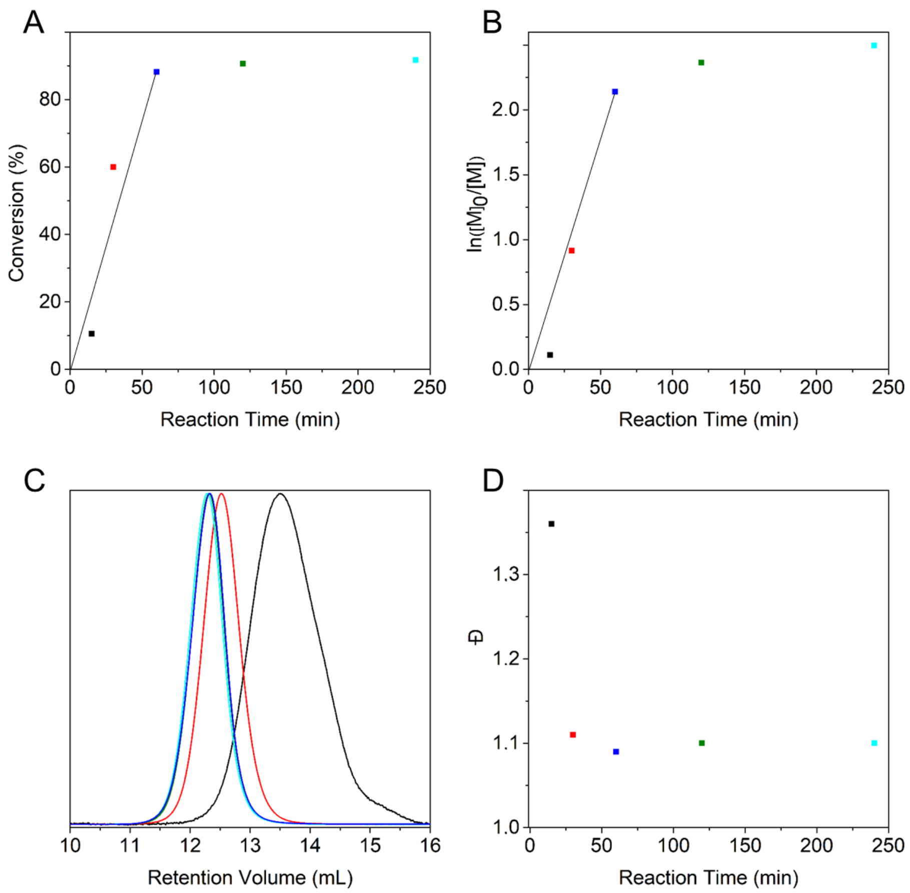

Figure S2: Polymerization kinetics of 3-isobutoxysulfopropyl acrylate by Cu(0)-RDRP. (A) Conversion and (B) ln($[M]_0$/[M]) kinetic plots extracted from ^{1}H-NMR analysis. (C) Elugrams and (D) dispersity plot obtained by SEC. Time points are 15 min (black), 30 min (red), 60 min (dark blue), 120 min (green) and 240 min (light blue). [EB*i*B]:[BSPA]:[DMSO]:[Me_6-TREN]:[$CuBr_2$] ratios used for the kinetic study were 1:94:200:0.09:0.01.

S3: ^{1}H-NMR analysis on deprotected homopolymers

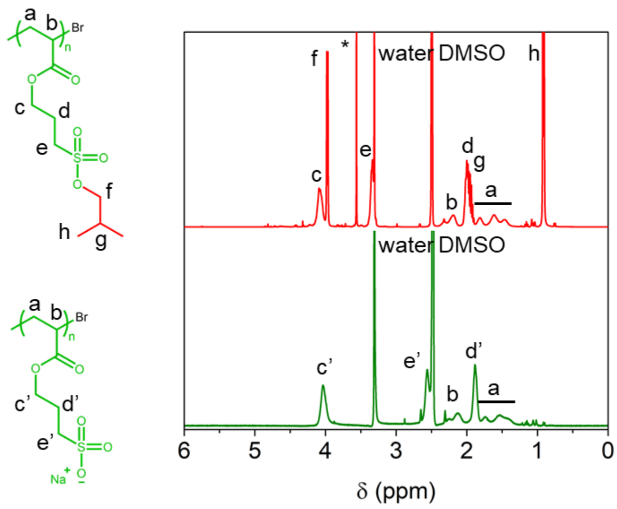

Figure S3-1: Comparative ^{1}H-NMR spectra of the $PBSPA_{45}$ (red, * = residual 1,4-dioxane) and corresponding $PSPA_{45}$ (green) in DMSO-d_6.

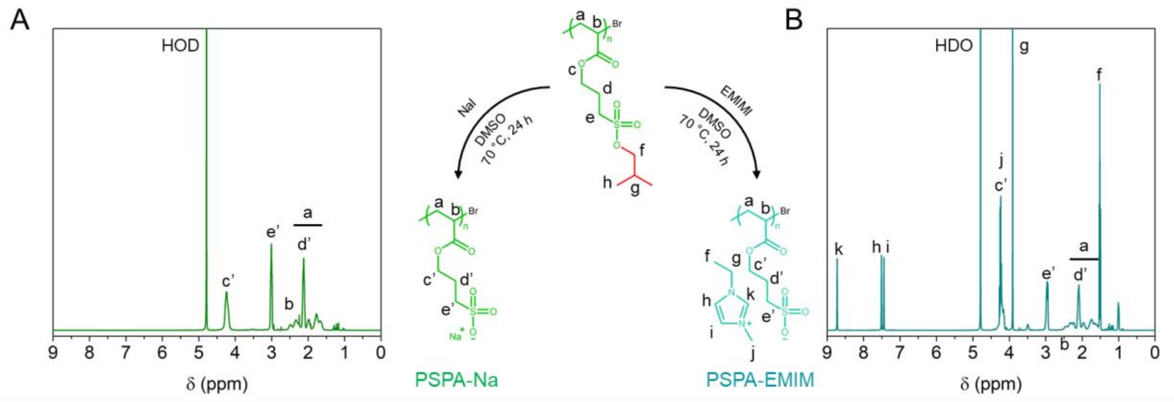

Figure S3-2: ^{1}H-NMR spectra (D_2O) of (A) PSPA-Na_{45} and (B) PSPA-$EMIM_{45}$ obtained after nucleophilic deprotection of $PBSPA_{45}$ using NaI or EMIMI respectively.

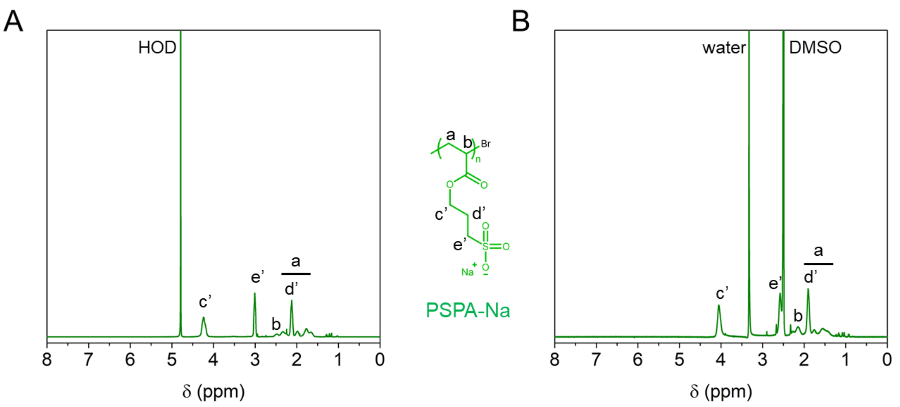

Figure S3-3: Comparative ^{1}H-NMR spectra of PSPA-Na_{45} in (A) D_2O and (B) DMSO-d_6.

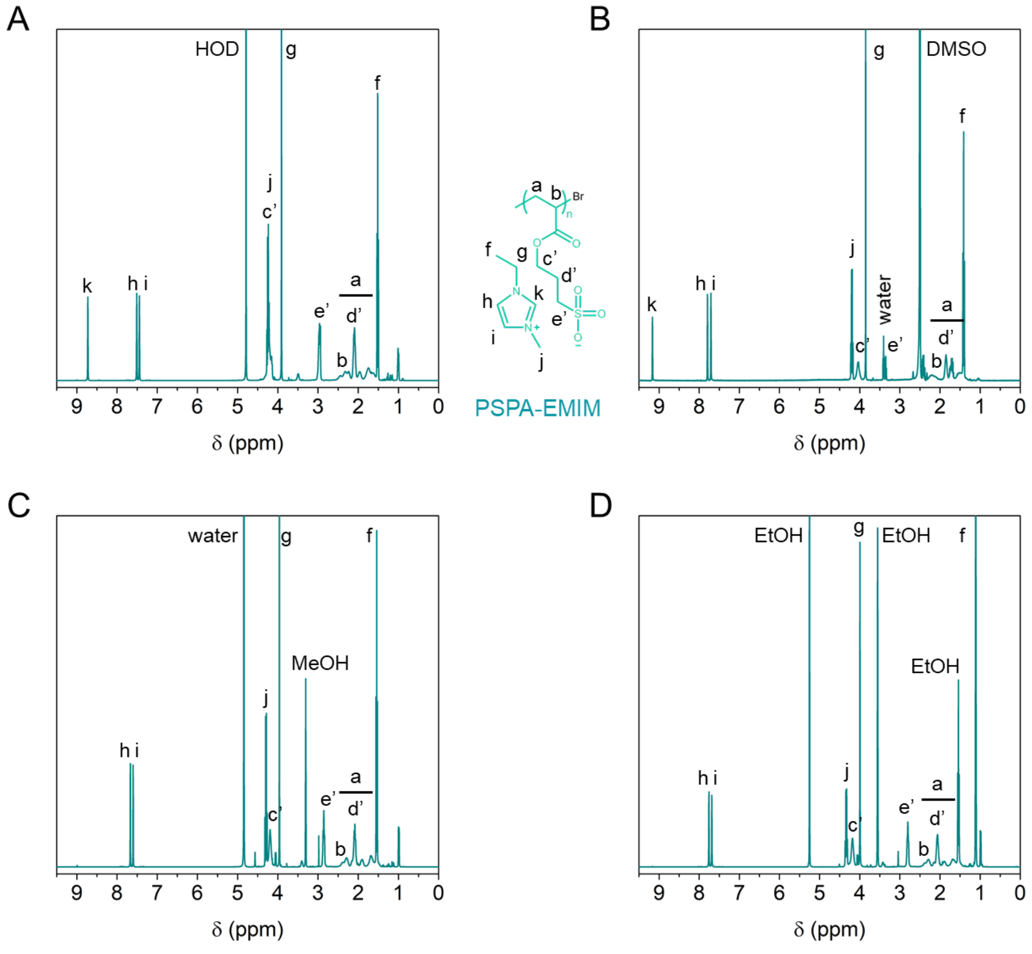

Figure S3-4: Comparative ^{1}H-NMR spectra of PSPA-$EMIM_{45}$ in (A) D_2O, (B) DMSO-d_6, (C) methanol-d_4 and (D) ethanol-d_6.

S4: Thermal analyses on the homopolymers

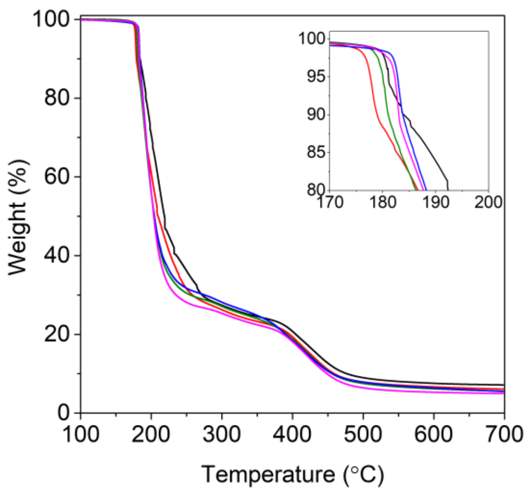

Figure S4-1: Comparative thermogravimetric analyses (TGA) on various protected homopolymers: $PBSPA_{45}$ (black), $PBSPA_{81}$ (red), $PBSPA_{210}$ (green), $PBSPA_{370}$ (blue) and $PBSPA_{530}$ (magenta).

Table S5: Degradation temperatures and associated weight losses obtained through thermogravimetric analyses on the various PBSPA homopolymers.

Sample	$T_{deg\ 1}$ ‡ (°C)	Weight loss at $T_{deg\ 1}$ (%)	$T_{deg\ 2}$ ‡ (°C)	Weight loss at $T_{deg\ 2}$ (%)
$PBSPA_{45}$	195	75.5	420	16.8
$PBSPA_{81}$	191	73.2	418	15.7
$PBSPA_{210}$	195	71.0	420	17.8
$PBSPA_{370}$	194	69.3	406	18.6
$PBSPA_{530}$	194	72.9	432	16.3

‡ temperature corresponding to the maximal rate at T_{deg} (*i.e.* decomposition temperature)

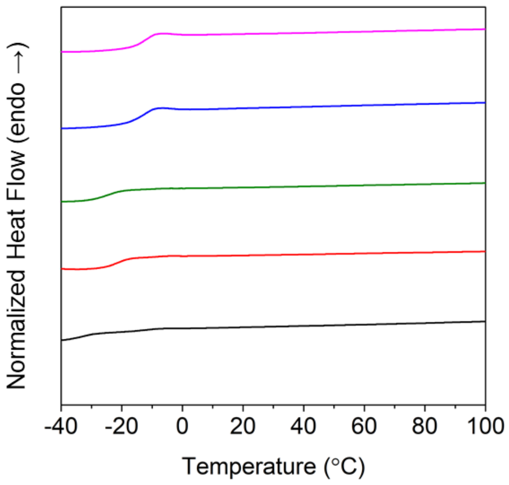

Figure S4-2: Comparative differential scanning calorimetry (DSC) analyses on various protected homopolymers: $PBSPA_{45}$ (black), $PBSPA_{81}$ (red), $PBSPA_{210}$ (green), $PBSPA_{370}$ (blue) and $PBSPA_{530}$ (magenta).

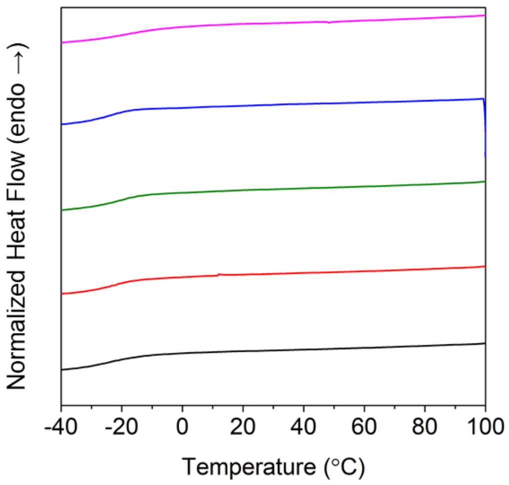

Figure S4-3: Differential scanning calorimetry analyses of various PSPA-EMIM homopolymers: PSPA-$EMIM_{45}$ (black, T_g = -23.5 °C), PSPA-$EMIM_{81}$ (red, T_g = -23.3 °C), PSPA-$EMIM_{210}$ (green, T_g = -20.4 °C), PSPA-$EMIM_{370}$ (blue, T_g = -24.5 °C) and PSPA-$EMIM_{530}$ (magenta, T_g = -16.8 °C).

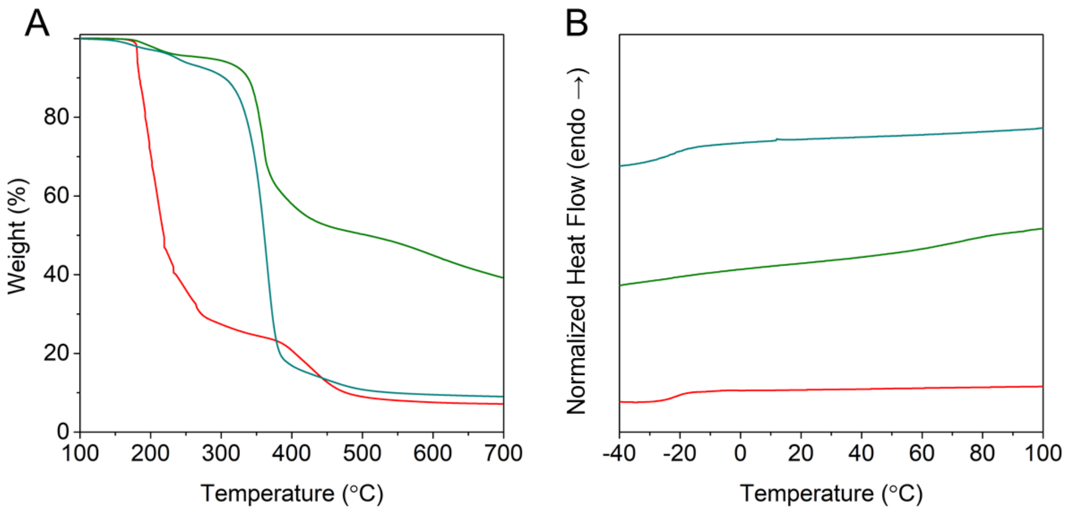

Figure S4-4: Comparative (A) thermogravimetric and (B) differential scanning calorimetry analyses on $PBSPA_{45}$ (red), PSPA-Na_{45} (green) and PSPA-$EMIM_{45}$ (dark cyan).

Table S6: Glass transition temperatures, degradation temperatures and associated weight losses observed through thermal analyses on a protected PBSPA homopolymer and various deprotected analogues.

Sample	T_g (°C)	$T_{deg\ 1}$‡ (°C)	Weight loss at $T_{deg\ 1}$ (%)	$T_{deg\ 2}$‡ (°C)	Weight loss at $T_{deg\ 2}$ (%)
$PBSPA_{45}$	-27.9	195	75.5	420	16.8
PSPA-Na_{45}	n.a.	186	5.28	355	42.3
PSPA-$EMIM_{45}$	-23.3	174/238*	2.67/4.01*	361	77.9

‡ temperature corresponding to the maximal rate at T_{deg} (*i.e.* decomposition temperature)

* two small degradation steps were observed *in-lieu* of the single one for other polymers, both of them are reported individually here.

S5: Evaporation tests on MA and BSPA monomers

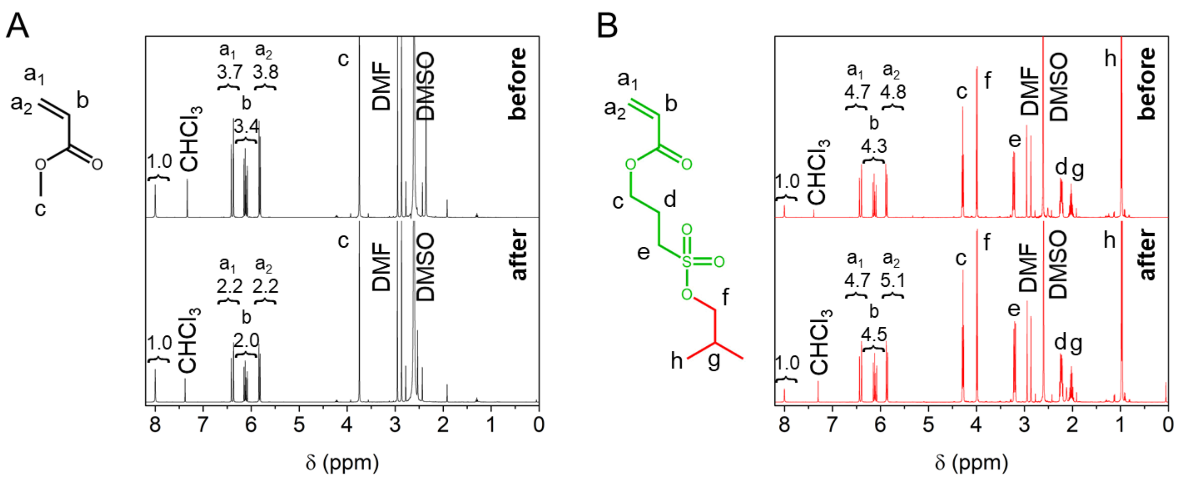

Figure S5: (A) ^{1}H-NMR spectra ($CDCl_3$) of methyl acrylate reaction mixtures before and after deoxygenation via 10 min bubbling with argon. Ratios between acrylic (CH 5.82, CH 6.11 and CH 6.38 ppm) and DMF (CH 8.00 ppm) signals significantly dropped after bubbling (~ 40 % loss). (B) ^{1}H-NMR spectra ($CDCl_3$) of 3-[isobutoxysulfonyl]propyl acrylate before and after deoxygenation via 10 min bubbling with argon. Ratios between acrylic (CH 5.87, CH 6.12 and CH 6.41 ppm) and DMF (CH 8.00 ppm) signals remained unaffected by bubbling.

S6: ^{1}H-NMR and SEC analyses of block copolymers

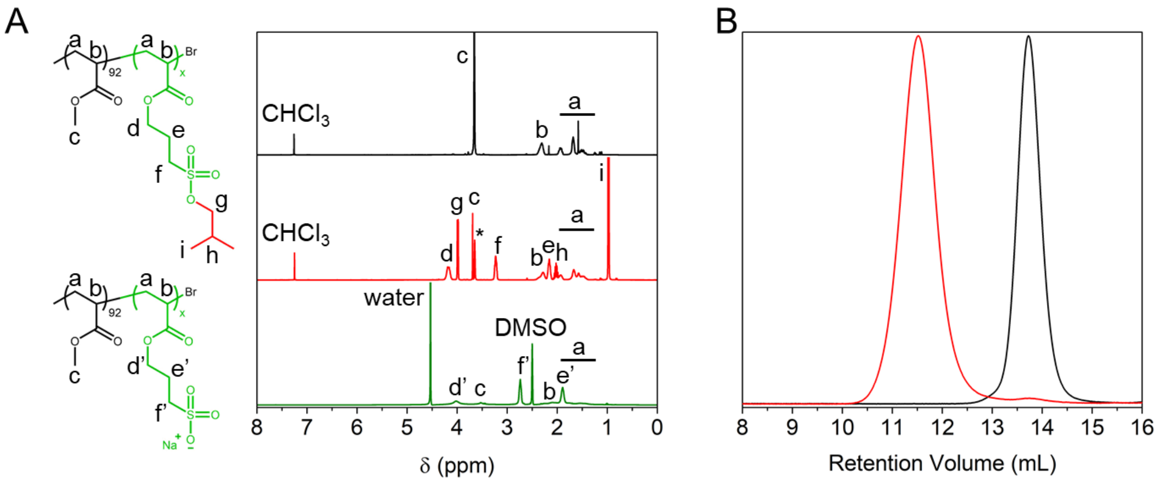

Figure S6-1: (A) ^{1}H-NMR spectra of the PMA_{92} macroinitiator (black, $CDCl_3$), PMA_{92}-*b*-$PBSPA_{231}$ protected BCP obtained by Cu(0)-RDRP (red, $CDCl_3$) and resulting amphiphilic PMA_{92}-*b*-PSPA-Na_{231} block copolymer (green, 40:60 vol.% DMSO-d_6:D_2O) obtained after nucleophilic deprotection. (B) SEC elugrams of PMA_{92} macroinitiator (black) and PMA_{92}-*b*-$PBSPA_{231}$ protected block copolymer (red).

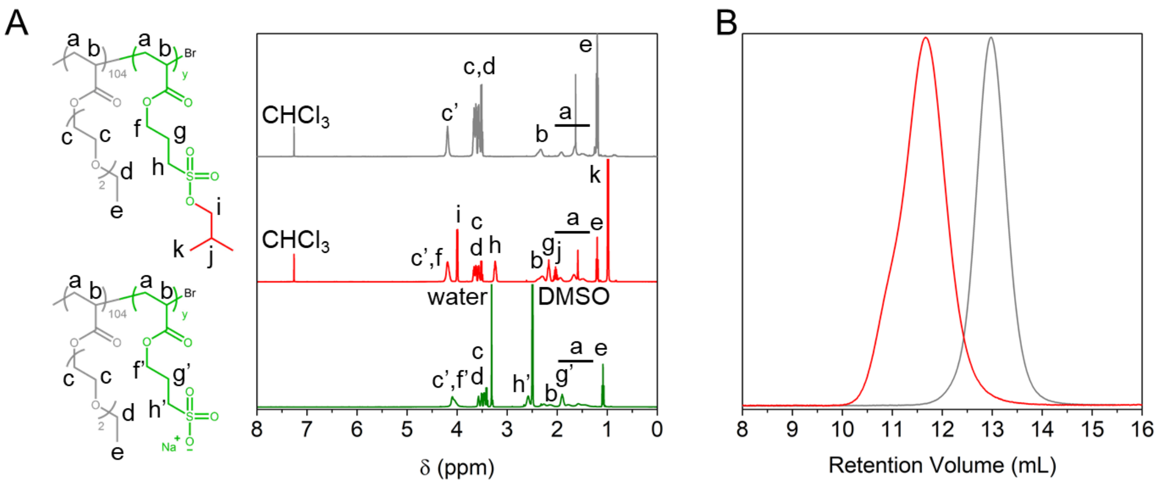

Figure S6-2: (A) ^{1}H-NMR spectra of the $PDEGA_{104}$ macroinitiator (grey, $CDCl_3$), $PDEGA_{104}$-*b*-$PBSPA_{228}$ protected BCP obtained by Cu(0)-RDRP (red, $CDCl_3$) and resulting $PDEGA_{104}$-*b*-PSPA-Na_{228} block copolymer (green, DMSO-d_6) obtained after nucleophilic deprotection. (B) SEC elugrams of $PDEGA_{104}$ macroinitiator (grey) and $PDEGA_{104}$-*b*-$PBSPA_{228}$ protected block copolymer (red).

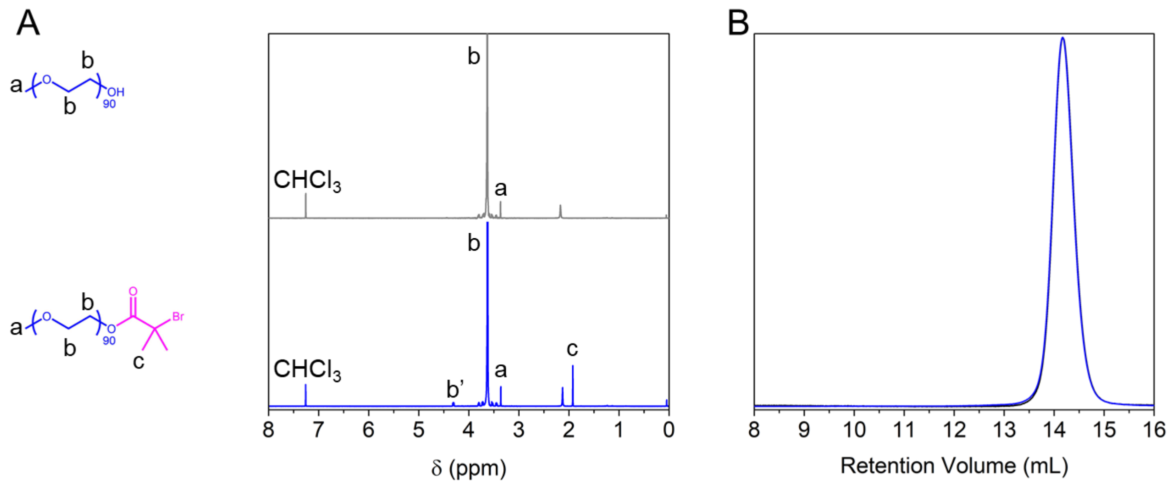

Figure S6-3: (A) ^{1}H-NMR spectra of the pristine PEO_{90}-OH (grey, $CDCl_3$) and PEO_{90}-Br macroinitiator (blue, $CDCl_3$) obtained after end-group modification. (B) SEC elugrams of the pristine PEO_{90}-OH (grey) and PEO_{90}-Br macroinitiator (blue).

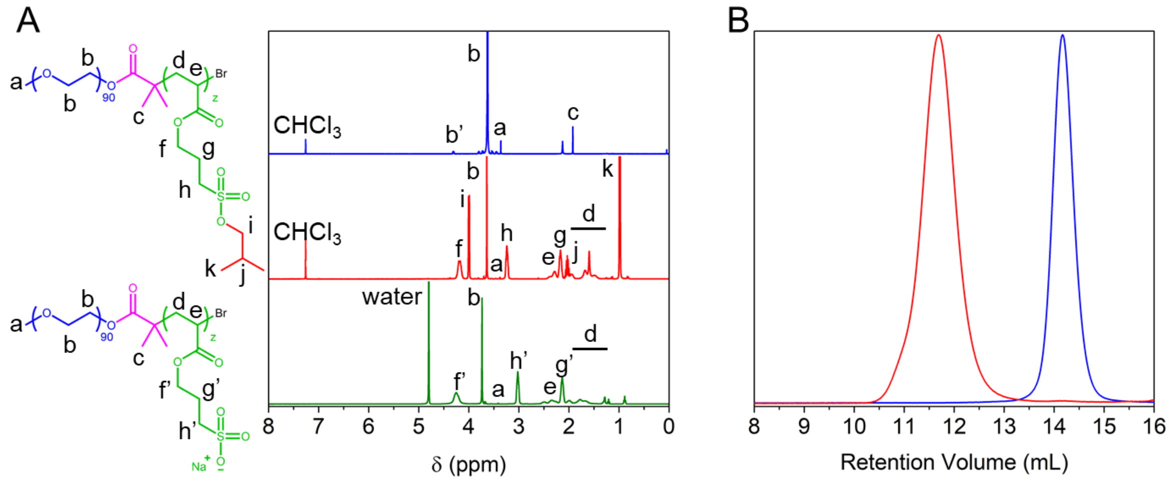

Figure S6-4: (A) ^{1}H-NMR spectra of the PEO_{90}-Br macroinitiator (blue, $CDCl_3$), PEO_{90}-*b*-$PBSPA_{237}$ protected BCP obtained by Cu(0)-RDRP (red, $CDCl_3$) and resulting amphiphilic PEO_{90}-*b*-PSPA-Na_{237} block copolymer (green, D_2O) obtained after nucleophilic deprotection. (B) SEC elugrams of PEO_{90}-Br macroinitiator (black) and PEO_{90}-*b*-$PBSPA_{237}$ protected block copolymer (red).

S7: Determination of the LCST of PDEGA homopolymer

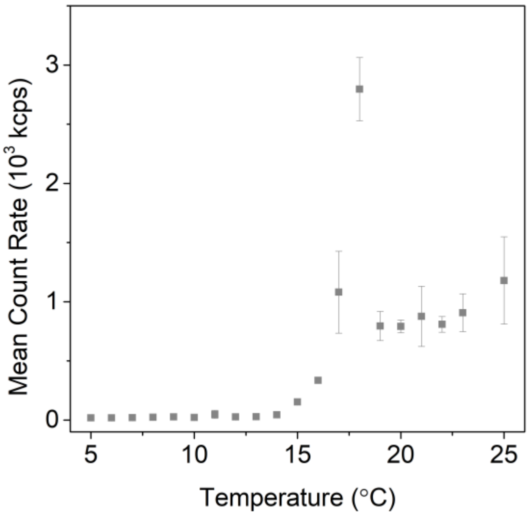

Figure S7-1: Plot of the mean count rate of a $PDEGA_{104}$ solution at 1 g L^{-1} in 10 mM KNO_3 as a function of temperature. All data points were measured in triplicate. The LCST can be approximated by the drop of the mean count rate when the temperature is decreased, here $LCST_{DLS}$ ≈ 14 °C.

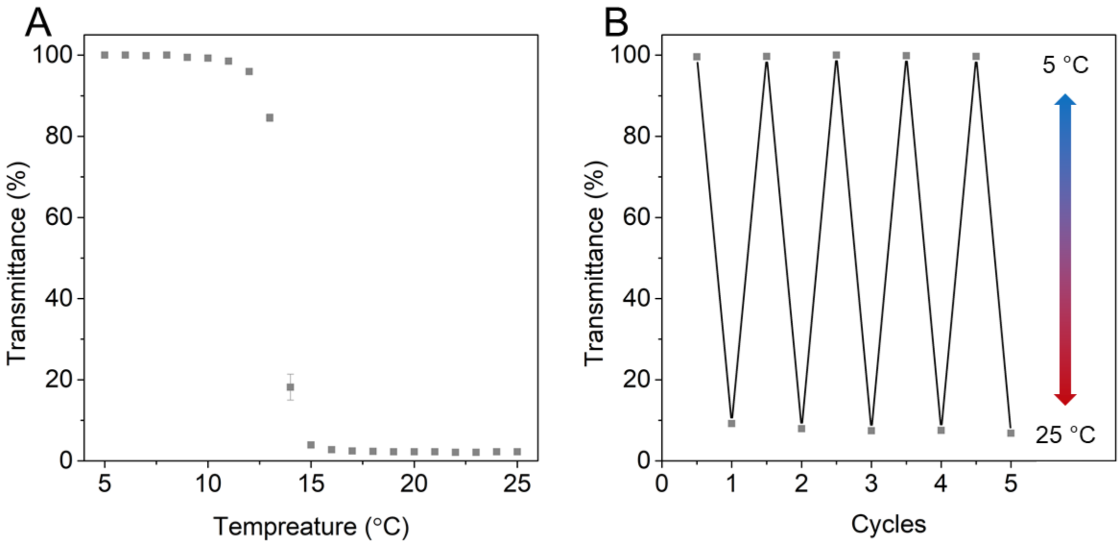

Figure S7-2: (A) Plot of the transmittance at 600 nm of a $PDEGA_{104}$ solution at 1 g L^{-1} in 10 mM KNO_3 as a function of the temperature and (B) cycles between low and high temperature and resulting transmittance values. The LCST can be more accurately measured in UV-Vis and the value was determined as the maximum of the inflection point, here $LCST_{UV\text{-}vis}$ = 14 °C. All data points were measured in triplicate.

S8: ‘Deprotection’ of macroinitiators (*i.e.* negative control)

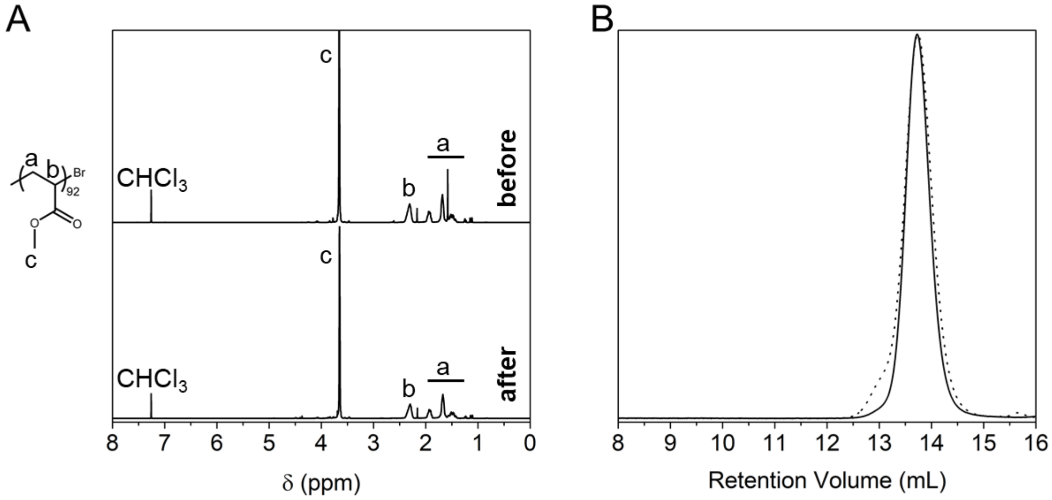

Figure S8-1: (A) ^{1}H-NMR spectra ($CDCl_3$) and (B) SEC elugrams of the PMA_{92} macroinitiator before (solid line) and after (dashed line) treatment with NaI.

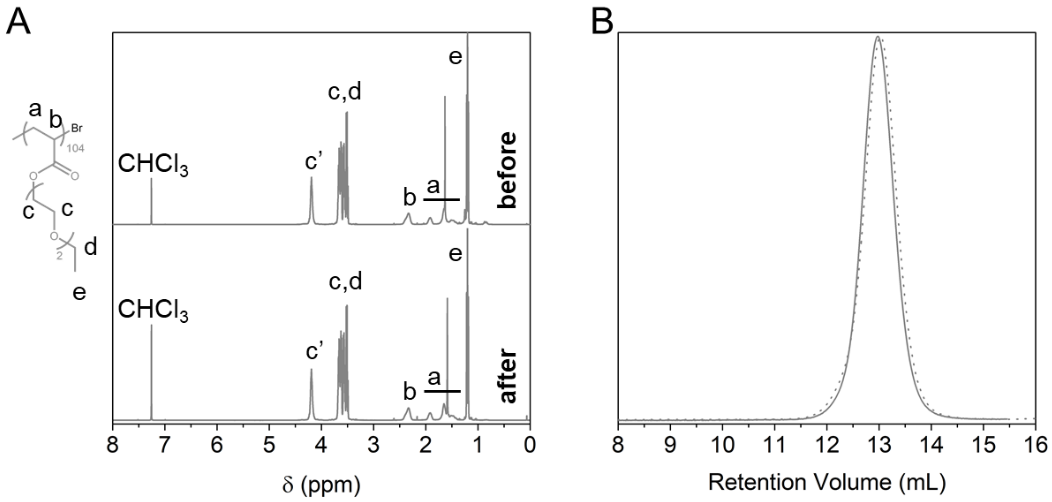

Figure S8-2: (A) ^{1}H-NMR spectra ($CDCl_3$) and (B) SEC elugrams of the $PDEGA_{104}$ macroinitiator before (solid line) and after (dashed line) treatment with NaI.

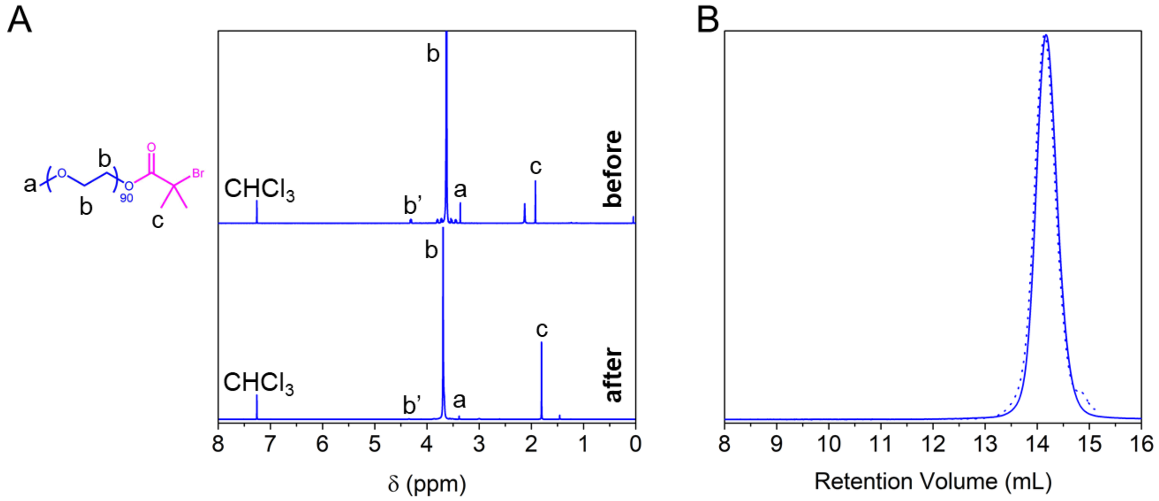

Figure S8-3: (A) ^{1}H-NMR spectra ($CDCl_3$) and (B) SEC elugrams of the PEO_{92}-Br macroinitiator before (solid line) and after (dashed line) treatment with NaI.

S9: ^{1}H-NMR analysis on EMIMI-deprotected block copolymers

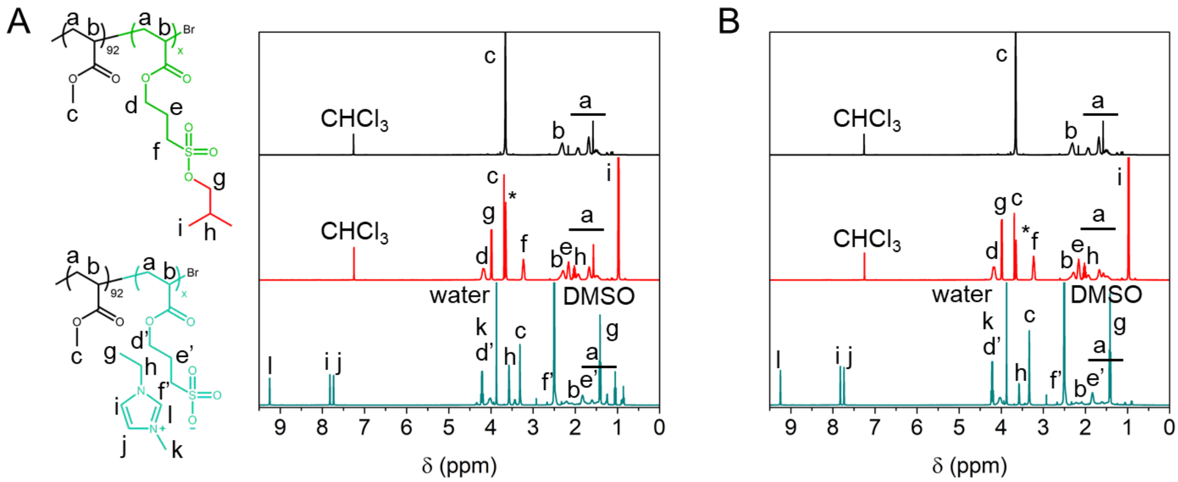

Figure S9-1: ^{1}H-NMR spectra of EMIMI-deprotected PMA-based block copolymers. (A) PMA_{92} (black, $CDCl_3$), PMA_{92}-*b*-$PBSPA_{103}$ (red, $CDCl_3$), PMA_{92}-*b*-PSPA-$EMIM_{103}$ (dark cyan, DMSO-d_6). (B) PMA_{92} (grey, $CDCl_3$), PMA_{92}-*b*-$PBSPA_{231}$ (red, $CDCl_3$), PMA_{92}-*b*-PSPA-$EMIM_{231}$ (dark cyan, DMSO-d_6).

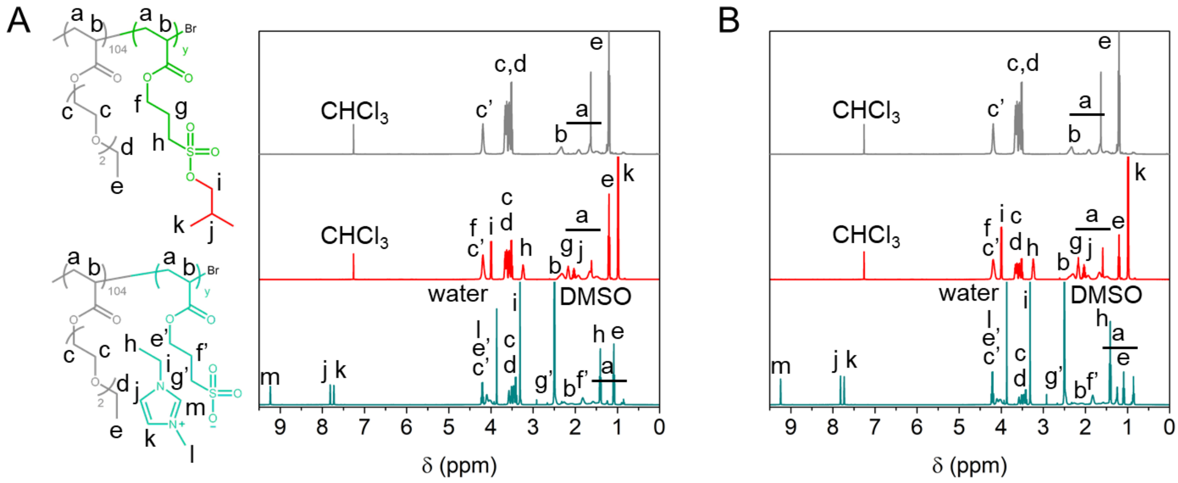

Figure S9-2: ^{1}H-NMR spectra of EMIMI-deprotected PDEGA-based block copolymers. (A) $PDEGA_{104}$ (grey, $CDCl_3$), $PDEGA_{104}$-*b*-$PBSPA_{94}$ (red, $CDCl_3$), $PDEGA_{104}$-*b*-PSPA-$EMIM_{94}$ (dark cyan, DMSO-d_6). (B) $PDEGA_{104}$ (grey, $CDCl_3$), $PDEGA_{104}$-*b*-$PBSPA_{228}$ (red, $CDCl_3$), $PDEGA_{104}$-*b*-PSPA-$EMIM_{228}$ (dark cyan, DMSO-d_6).

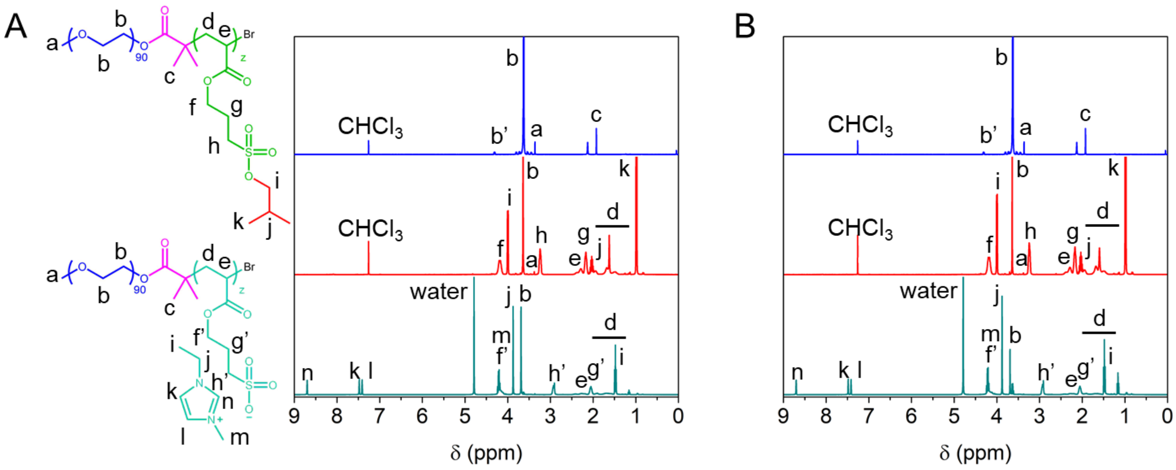

Figure S9-3: ^{1}H-NMR spectra of EMIMI-deprotected PEO-based block copolymers. (A) PEO_{90}-Br (blue, $CDCl_3$), PEO_{90}-*b*-$PBSPA_{110}$ (red, $CDCl_3$) and PEO_{90}-*b*-PSPA-$EMIM_{110}$ (dark cyan, D_2O). (B) PEO_{90}-Br (blue, $CDCl_3$), PEO_{90}-*b*-$PBSPA_{237}$ (red, $CDCl_3$) and PEO_{90}-*b*-PSPA-$EMIM_{237}$ (dark cyan, D_2O).

S10: Thermal analyses on the EMIMI-deprotected block copolymers

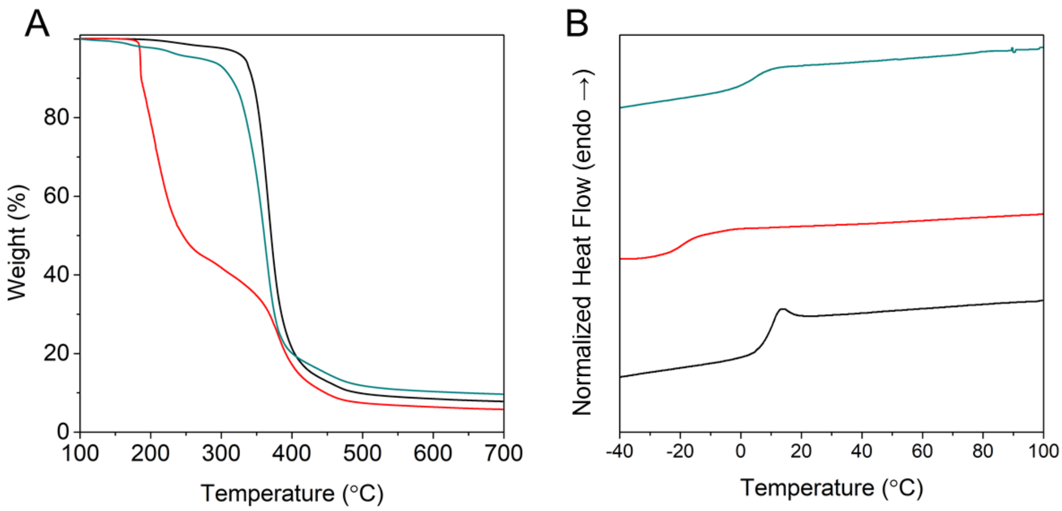

Figure S10-1: (A) TGA and (B) DSC thermograms of one EMIMI-deprotected PMA-based block copolymer: PMA_{92} (black), PMA_{92}-*b*-$PBSPA_{103}$ (red) and PMA_{92}-*b*-PSPA-$EMIM_{103}$ (dark cyan).

Table S7: Glass transition temperatures, degradation temperatures and associated weight losses obtained through thermal analyses on the PMA macroinitiator and PMA-based block copolymers.

Sample	T_g (°C)
PMA_{92}	8.3
PMA_{92}-*b*-$PBSPA_{103}$	-20.3
PMA_{92}-*b*-PSPA-Na_{103}	n.a.
PMA_{92}-*b*-PSPA-$EMIM_{103}$	3.4
PMA_{92}-*b*-$PBSPA_{231}$	-17.4
PMA_{92}-*b*-PSPA-Na_{231}	n.a.
PMA_{92}-*b*-PSPA-$EMIM_{231}$	-2.2

determined using a 10 °C min^{-1} heating rate

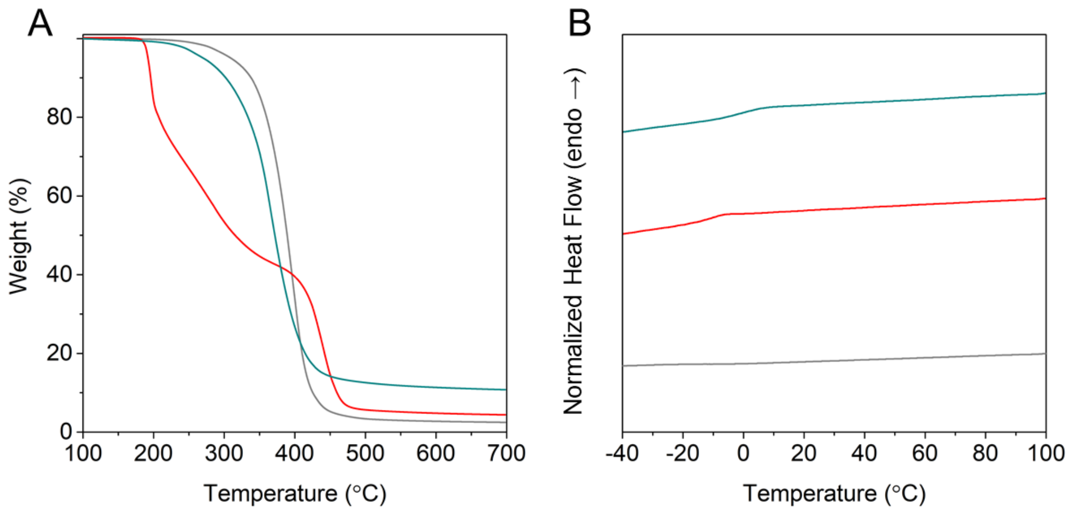

Figure S10-2: (A) TGA and (B) DSC thermograms of one EMIMI-deprotected PDEGA-based block copolymer: $PDEGA_{104}$ (grey), $PDEGA_{104}$-*b*-$PBSPA_{94}$ (red), $PDEGA_{104}$-*b*-PSPA-$EMIM_{94}$ (dark cyan).

Table S8: Glass transition temperatures, degradation temperatures and associated weight losses obtained through thermal analyses on the PMA macroinitiator and PMA-based block copolymers.

Sample	T_g (°C)
$PDEGA_{104}$	n.a.
$PDEGA_{104}$-*b*-$PBSPA_{94}$	-11.3
$PDEGA_{104}$-*b*-PSPA-Na_{94}	n.a.
$PDEGA_{104}$-*b*-PSPA-$EMIM_{94}$	-0.4
$PDEGA_{104}$-*b*-$PBSPA_{228}$	-9.6
$PDEGA_{104}$-*b*-PSPA-Na_{228}	n.a.
$PDEGA_{104}$-*b*-PSPA-$EMIM_{228}$	2.1

determined using a 10 °C min^{-1} heating rate

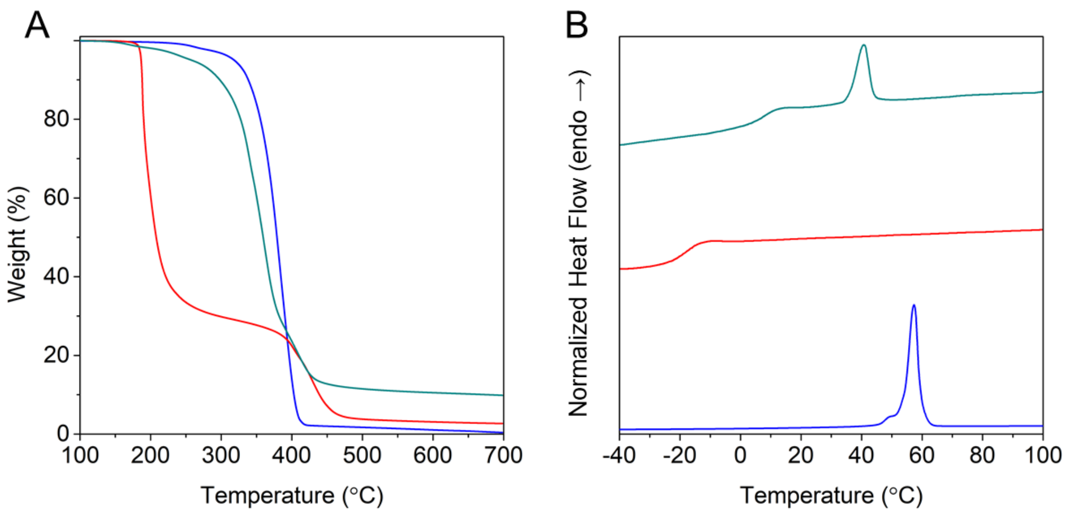

Figure S10-3: (A) TGA and (B) DSC thermograms of one EMIMI-deprotected PEO-based block copolymer: PEO_{90}-Br (blue), PEO_{90}-*b*-$PBSPA_{237}$ (red) and PEO_{90}-*b*-PSPA-$EMIM_{237}$ (dark cyan).

Table S9: Glass transition temperatures, degradation temperatures and associated weight losses obtained through thermal analyses on the PMA macroinitiator and PMA-based block copolymers.

Sample	T_g (°C)	T_m (°C)	Enthalpy T_m (J g^{-1})	T_c (°C)	Enthalpy T_c (J g^{-1})
PEO_{90}	n.a.	57.3	152	34.7	37.1
PEO_{90}-*b*-$PBSPA_{110}$	-24.1	48.8	1.00	n.a.	n.a.
PEO_{90}-*b*-PSPA-Na_{110}	n.a.	33.8	9.50	-40.1	4.50
PEO_{90}-*b*-PSPA-$EMIM_{110}$	5.4	n.a.	n.a.	n.a.	n.a.
PEO_{90}-*b*-$PBSPA_{237}$	-18.4	n.a.	n.a.	n.a.	n.a.
PEO_{90}-*b*-PSPA-Na_{237}	n.a.	35.6	4.90	-36.4	3.34
PEO_{90}-*b*-PSPA-$EMIM_{237}$	6.4	35.8	5.65	-35.4	1.85

determined using a 10 °C min^{-1} heating rate

S1: Electron microscopy on the self-assembled PMA-based nanoparticles

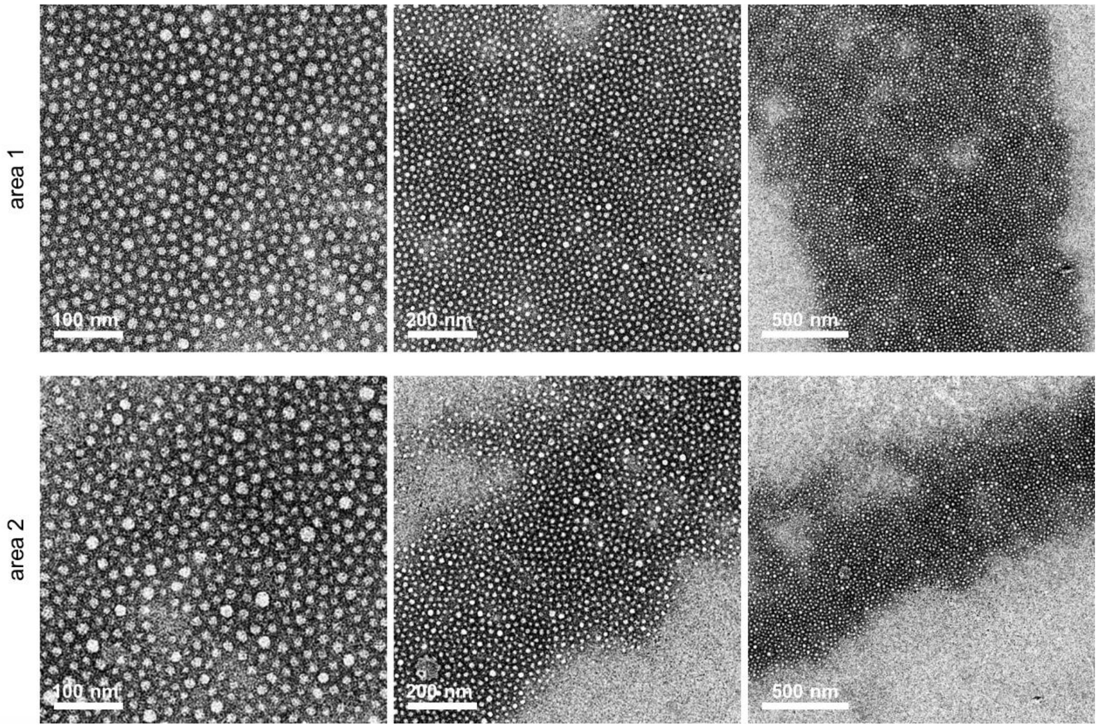

Figure S11-1: TEM images of uranyl acetate-stained nanoparticles self-assembled from a solution of PMA_{92}-*b*-$PSPA\text{-}Na_{103}$ amphiphilic block copolymer at 1 g L^{-1} in 10 mM KNO_3.

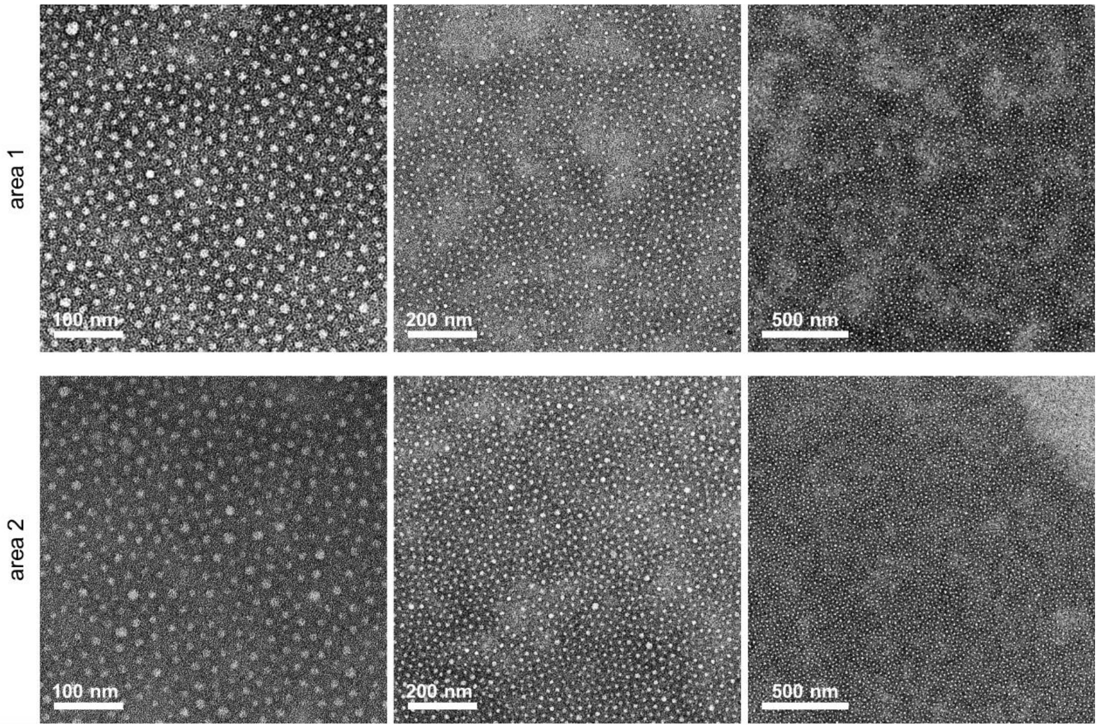

Figure S11-2: TEM images of uranyl acetate-stained nanoparticles self-assembled from a solution of PMA_{92}-*b*-$PSPA\text{-}Na_{231}$ amphiphilic block copolymer at 1 g L^{-1} in 10 mM KNO_3.

S12: Dynamic light scattering analysis on the PDEGA-based nanoparticles

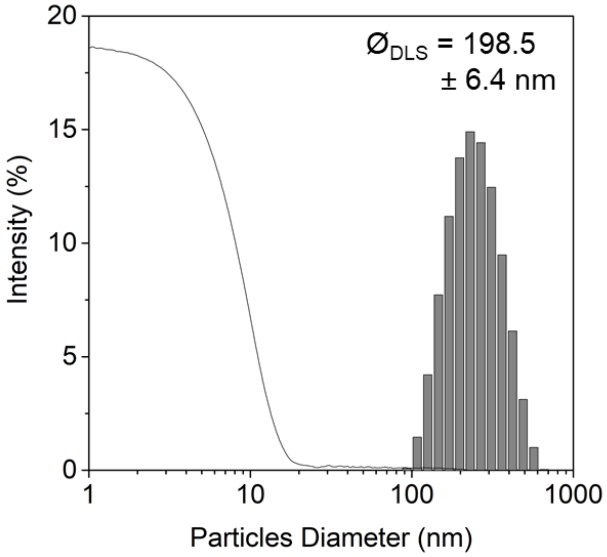

Figure S12-1: DLS intensity plot of the nanoparticles achieved from direct dissolution of $PDEGA_{104}$-*b*-$PSPA\text{-}Na_{228}$ at 1 g L^{-1} in 10 mM KNO_3.

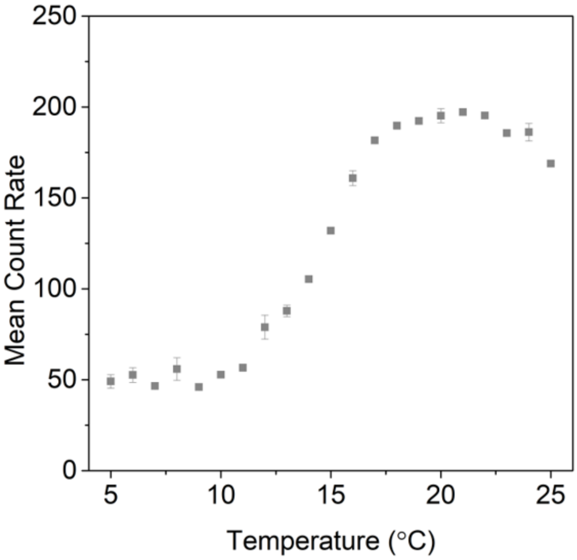

Figure S12-2: Plot of the mean count rate *vs.* the temperature enabled the determination of the LCST of the $PDEGA_{104}$ segment (*i.e*. hydrophobic domain) of the PDEGA-based nanoparticles, here $PDEGA_{104}$-*b*-$PSPA\text{-}Na_{94}$ was studied at a concentration of 1 g L^{-1} (10 mM KNO_3) .

S13: Characterisation of the pristine and quaternised poly(4-vinylpyridine)

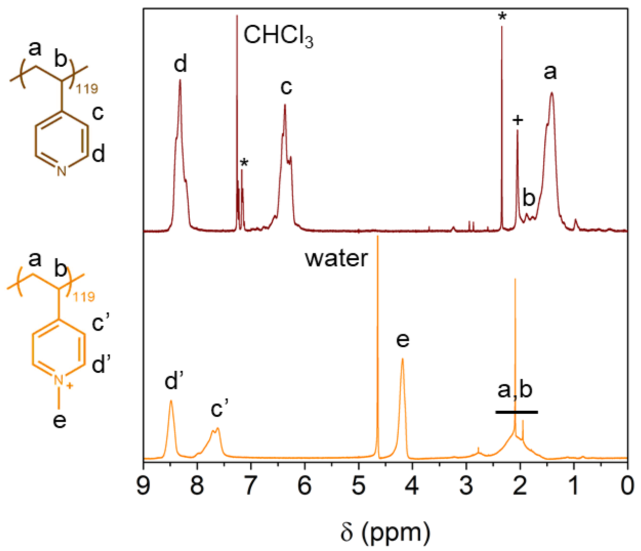

Figure S13-1: ^{1}H-NMR spectra of the $P4VP_{119}$ (wine red, $CDCl_3$) and $P4VPq_{119}$ (orange, D_2O). *: residual toluene, $^+$: residual water.

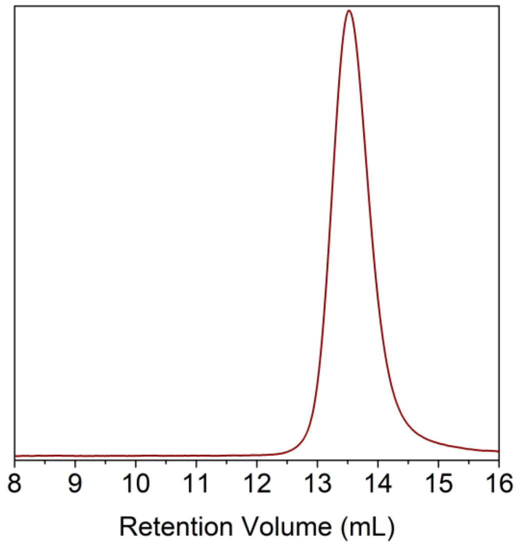

Figure S13-2: SEC elugram of P4VP homopolymer measured at 50 °C in DMF with 0.01 M LiBr.

S14: Dynamic light scattering analysis on the long PEO-based C3Ms

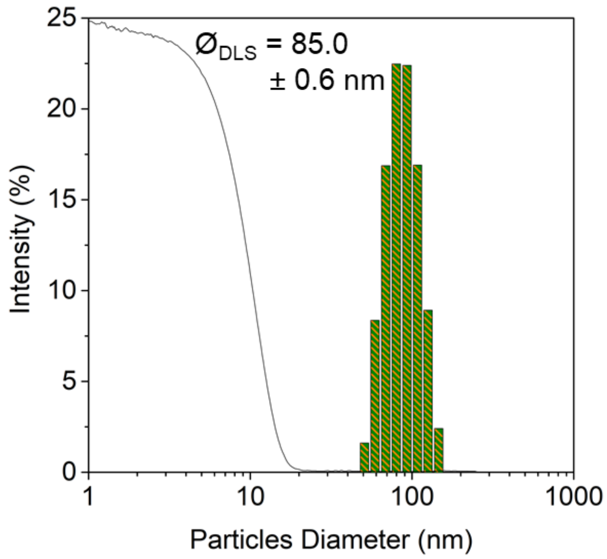

Figure S14: DLS intensity plot of the C3Ms obtained through electrostatic complexation between PEO_{90}-*b*-$PSPA\text{-}Na_{237}$ (1 g L^{-1} in 10 mM KNO_3 solution) and $P4VPq_{116}$ (1 g L^{-1} in 10 mM KNO_3 solution).

S15: Atomic force microscopy on the PEO-based C3Ms

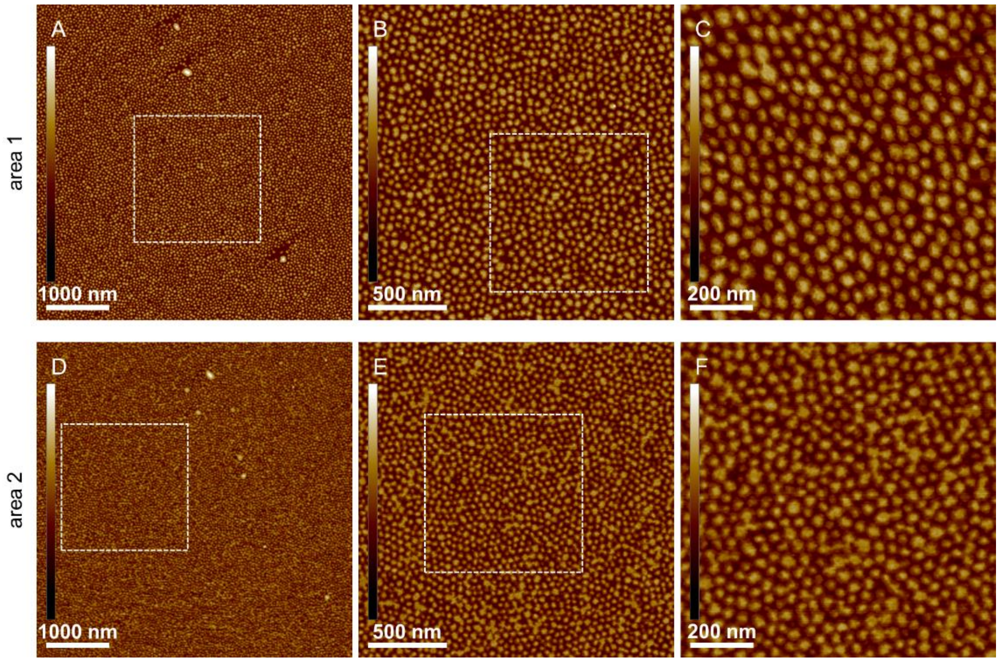

Figure S15-1: AFM height images of the C3Ms obtained through electrostatic complexation between PEO_{90}-*b*-PSPA-Na_{110} (1 g L^{-1} in 10 mM KNO_3 solution) and $P4VPq_{116}$ (1 g L^{-1} in 10 mM KNO_3 solution) deposited on mica discs (A-C and D-E). z-scale is ± 2.5 nm.

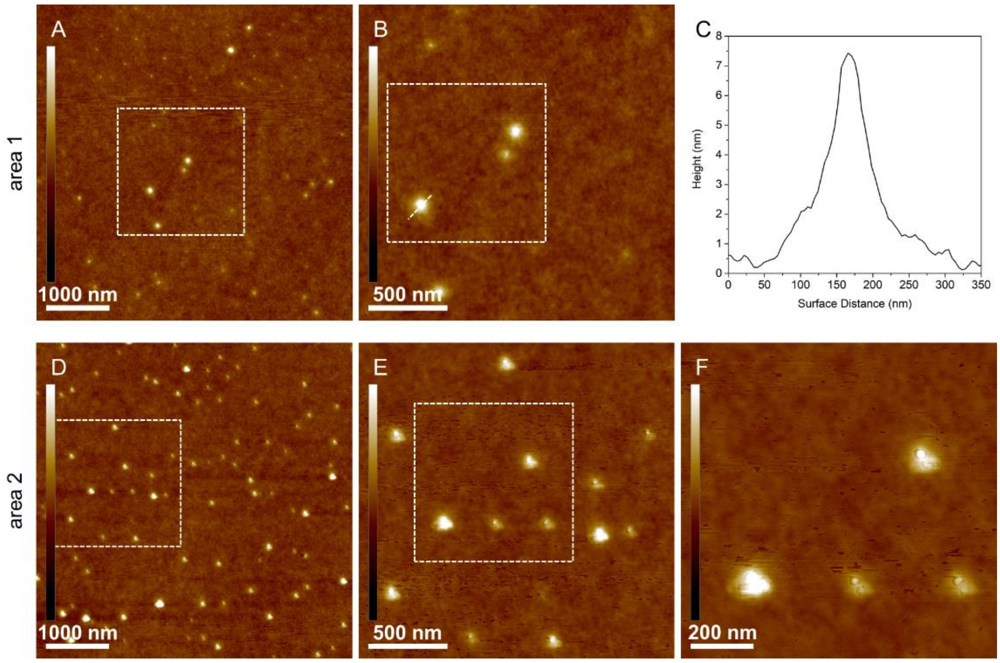

Figure S15-2: (A,B and D-F) AFM height images of the C3Ms obtained through electrostatic complexation between PEO_{90}-*b*-PSPA- Na_{237} (1 g L^{-1} in 10 mM KNO_3 solution) and $P4VPq_{116}$ (1 g L^{-1} in 10 mM KNO_3 solution) deposited on mica discs. (D) cross-sectional analysis across a nanoparticle (dashed line in image B). z-scale is ± 5 nm.

S16: Electron microscopy on the PEO-based C3Ms

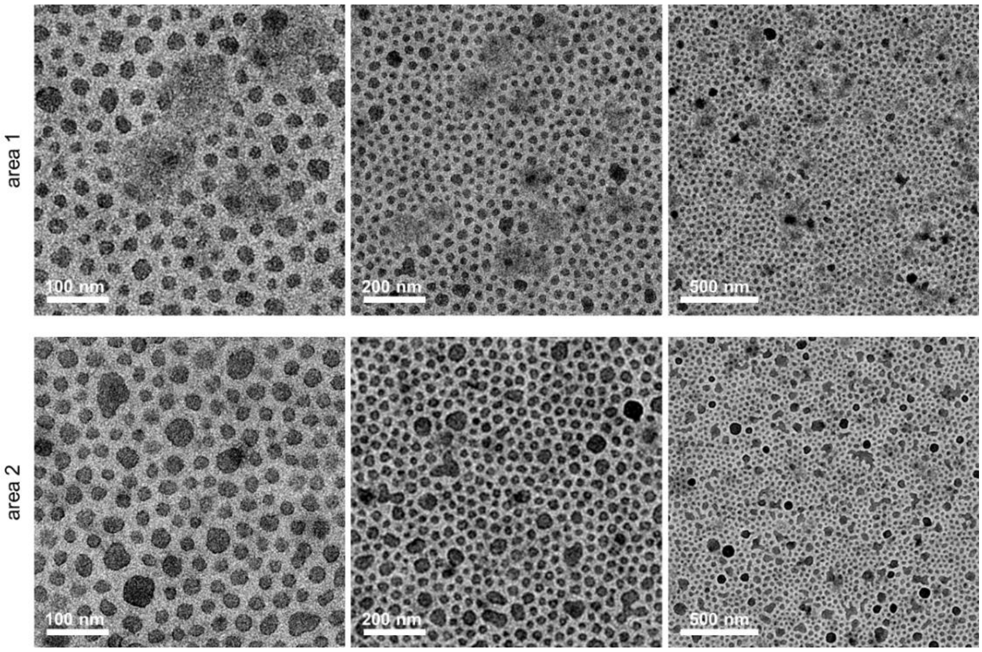

Figure S16-1: TEM images of uranyl acetate-stained C3M obtained through electrostatic complexation between PEO_{90}-*b*-PSPA-Na_{110} (1 g L^{-1} in 10 mM KNO_3 solution) and $P4VPq_{116}$ (1 g L^{-1} in 10 mM KNO_3 solution).

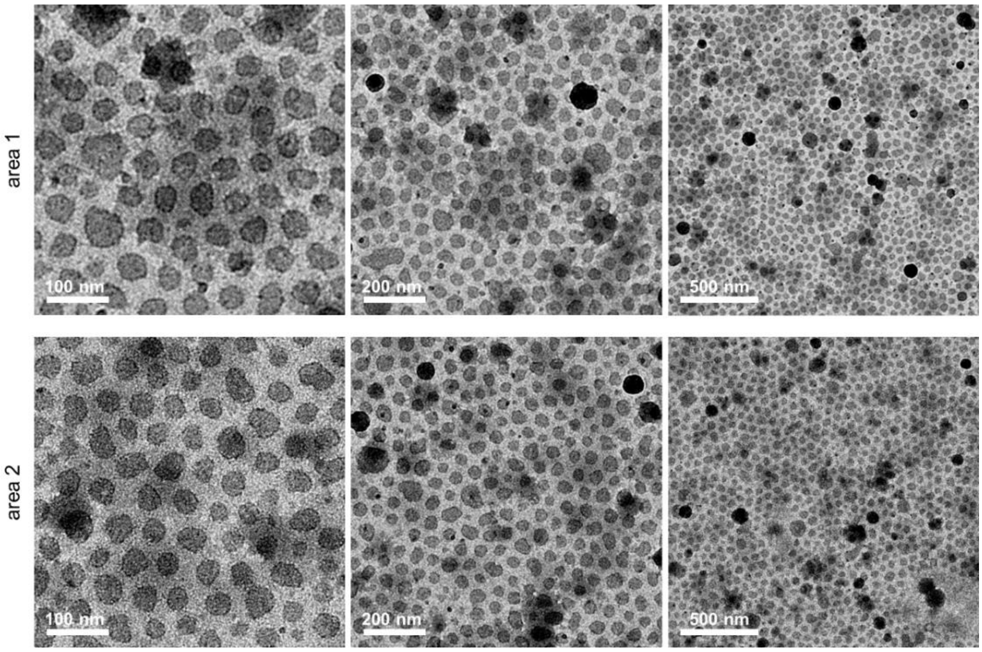

Figure S16-2: TEM images of uranyl acetate-stained C3Ms obtained through electrostatic complexation between PEO_{90}-*b*-PSPA- Na_{237} (1 g L^{-1} in 10 mM KNO_3 solution) and $P4VPq_{116}$ (1 g L^{-1} in 10 mM KNO_3 solution).

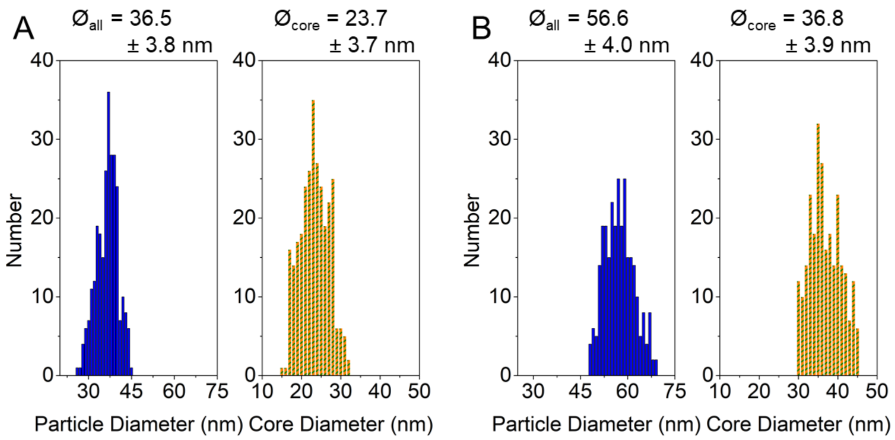

Figure S16-3: Statistical analyses on the core and overall diameters of the C3M particles obtained through electrostatic complexation between (A) PEO_{90}-*b*-PSPA-Na_{110} or (B) PEO_{90}-*b*-PSPA-Na_{237} (both at 1 g L^{-1} in 10 mM KNO_3 solution) and $P4VPq_{116}$ (1 g L^{-1} in 10 mM KNO_3 solution). Data extracted from 250 specimen across multiple TEM images.

S17: One-pot synthesis of poly(3-sulfopropyl acrylate) sodium salt

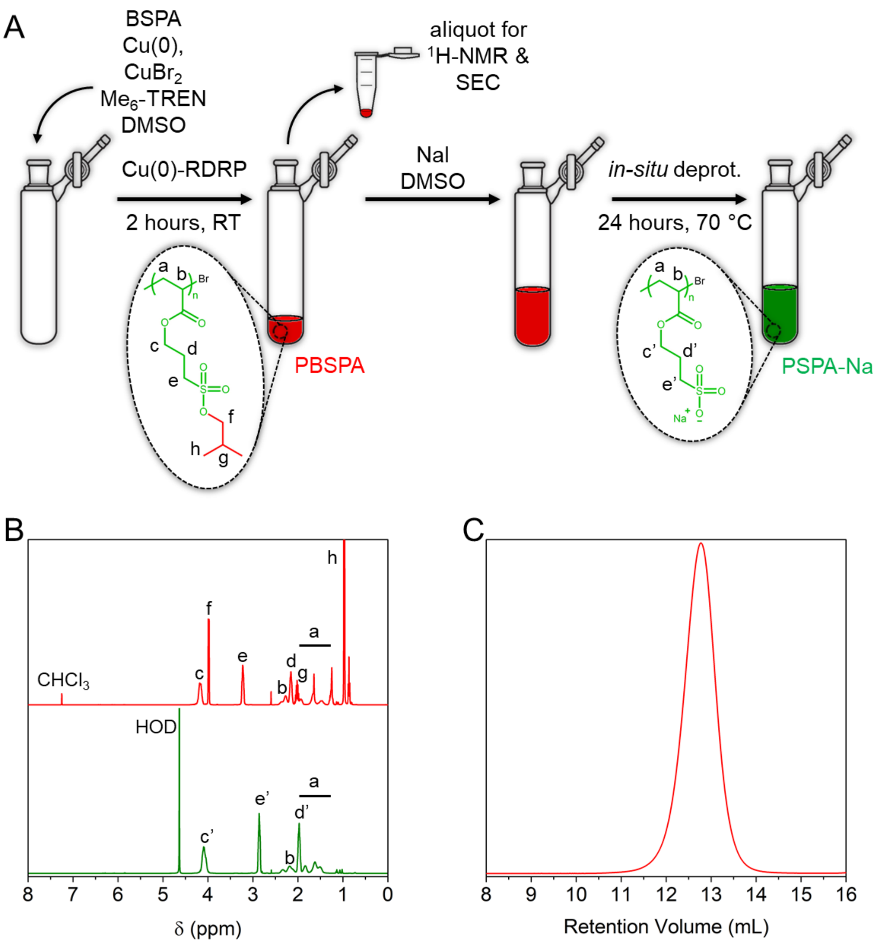

Figure S17: Strong polyanions can be produced in one-pot fashion through polymerization and *in-situ* nucleophilic deprotection. (A) Schematic depiction of the procedure and characterisation of the resulting polymer by (B) ^{1}H-NMR and (C) SEC.

Supporting References

1. Hofman, A. H.; Fokkink, R.; Kamperman, M., A mild and quantitative route towards well-defined strong anionic/hydrophobic diblock copolymers: synthesis and aqueous self-assembly. *Polym. Chem.* **2019,** *10* (45), 6109-6115.
2. Du, J.; Armes, S. P., pH-Responsive Vesicles Based on a Hydrolytically Self-Cross-Linkable Copolymer. *J. Am. Chem. Soc.* **2005,** *127* (37), 12800-12801.
3. Löbling, T. I.; Haataja, J. S.; Synatschke, C. V.; Schacher, F. H.; Müller, M.; Hanisch, A.; Gröschel, A. H.; Müller, A. H. E., Hidden Structural Features of Multicompartment Micelles Revealed by Cryogenic Transmission Electron Tomography. *ACS Nano* **2014,** *8* (11), 11330-11340.
4. Hofman, A. H.; Alberda van Ekenstein, G. O. R.; Woortman, A. J. J.; ten Brinke, G.; Loos, K., Poly(4-vinylpyridine)-block-poly(N-acryloylpiperidine) diblock copolymers: synthesis, self-assembly and interaction. *Polym. Chem.* **2015,** *6* (39), 7015-7026.
5. Sadman, K.; Wang, Q.; Chen, Y.; Keshavarz, B.; Jiang, Z.; Shull, K. R., Influence of Hydrophobicity on Polyelectrolyte Complexation. *Macromolecules* **2017,** *50* (23), 9417-9426.
6. Li, A.; Li, Z.; Zhang, S.; Sun, G.; Policarpio, D. M.; Wooley, K. L., Synthesis and Direct Visualization of Dumbbell-Shaped Molecular Brushes. *ACS Macro Lett.* **2012,** *1* (1), 241-245.
7. Sherck, N. J.; Kim, H. C.; Won, Y.-Y., Elucidating a Unified Mechanistic Scheme for the DBU-Catalyzed Ring-Opening Polymerization of Lactide to Poly(lactic acid). *Macromolecules* **2016,** *49* (13), 4699-4713.
